# Predictive shifts in free energy couple mutations to their phenotypic consequences

**DOI:** 10.1073/pnas.1907869116

**Published:** 2019-08-26

**Authors:** Griffin Chure, Manuel Razo-Mejia, Nathan M. Belliveau, Tal Einav, Zofii A. Kaczmarek, Stephanie L. Barnes, Mitchell Lewis, Rob Phillips

**Affiliations:** ^a^Department of Biology and Biological Engineering, California Institute of Technology, Pasadena, CA 91125;; ^b^Department of Physics, California Institute of Technology, Pasadena, CA 91125;; ^c^Department of Biochemistry and Molecular Biophysics, University of Pennsylvania School of Medicine, Philadelphia, PA 19104

**Keywords:** transcriptional regulation, allostery, statistical mechanics, biophysics, mutation

## Abstract

Mutation is a critical mechanism by which evolution explores the functional landscape of proteins. Despite our ability to experimentally inflict mutations at will, it remains difficult to link sequence-level perturbations to systems-level responses. Here, we present a framework centered on measuring changes in the free energy of the system to link individual mutations in an allosteric transcriptional repressor to the parameters which govern its response. We find that the energetic effects of the mutations can be categorized into several classes which have characteristic curves as a function of the inducer concentration. We experimentally test these diagnostic predictions using the well-characterized LacI repressor of *Escherichia coli*, probing several mutations in the DNA binding and inducer binding domains. We find that the change in gene expression due to a point mutation can be captured by modifying only the model parameters that describe the respective domain of the wild-type protein. These parameters appear to be insulated, with mutations in the DNA binding domain altering only the DNA affinity and those in the inducer binding domain altering only the allosteric parameters. Changing these subsets of parameters tunes the free energy of the system in a way that is concordant with theoretical expectations. Finally, we show that the induction profiles and resulting free energies associated with pairwise double mutants can be predicted with quantitative accuracy given knowledge of the single mutants, providing an avenue for identifying and quantifying epistatic interactions.

## Significance

We present a biophysical model of allosteric transcriptional regulation that directly links the location of a mutation within a repressor to the biophysical parameters that describe its behavior. We explore the phenotypic space of a repressor with mutations in either the inducer binding or DNA binding domains. Using the LacI repressor in *Escherichia coli*, we make sharp, falsifiable predictions and use this framework to generate a null hypothesis for how double mutants behave, given knowledge of the single mutants. Linking mutations to the parameters which govern the system allows for quantitative predictions of how the free energy of the system changes as a result, permitting coarse graining of high-dimensional data into a single-parameter description of the mutational consequences.

Thermodynamic treatments of transcriptional regulation have been fruitful in their ability to generate quantitative predictions of gene expression as a function of a minimal set of physically meaningful parameters (1–13). These models quantitatively describe numerous properties of input–output functions, such as the leakiness, saturation, dynamic range, steepness of response, and [$EC_{50}$]—the concentration of inducer at which the response is half-maximal. The mathematical forms of these phenotypic properties are couched in terms of a minimal set of experimentally accessible variables, such as the inducer concentration, transcription factor copy number, and the DNA sequence of the binding site (10). While the amino acid sequence of the transcription factor is another controllable variable, it is seldom implemented in quantitative terms, considering that mutations with subtle changes in chemistry frequently yield unpredictable physiological consequences. In this work, we examine how a series of mutations in either the DNA binding or inducer binding domains of a transcriptional repressor influence the values of the biophysical parameters which govern its regulatory behavior.

We first present a theoretical framework for understanding how mutations in the repressor affect different parameters and alter the free energy of the system. The multidimensional parameter space of the aforementioned thermodynamic models is highly degenerate, with multiple combinations of parameter values yielding the same phenotypic response. This degeneracy can be subsumed into the free energy of the system, transforming the input–output function into a 1-dimensional description with the form of a Fermi function (14, 15). We find that the parameters capturing the allosteric nature of the repressor, the repressor copy number, and the DNA binding specificity contribute independently to the free energy of the system with different degrees of sensitivity. Furthermore, changes restricted to 1 of these 3 groups of parameters result in characteristic changes in the free energy relative to the wild-type repressor, providing falsifiable predictions of how different classes of mutations should behave.

Next, we test these descriptions experimentally using the well-characterized transcriptional repressor of the *lac* operon LacI in *Escherichia coli* regulating expression of a fluorescent reporter. We introduce a series of point mutations in either the inducer binding or DNA binding domain. We then measure the full induction profile of each mutant, determine the minimal set of parameters that are affected by the mutation, and predict how each mutation tunes the free energy at different inducer concentrations, repressor copy numbers, and DNA binding strengths. We find in general that mutations in the DNA binding domain only influence DNA binding strength and that mutations within the inducer binding domain affect only the parameters which dictate the allosteric response. The degree to which these parameters are insulated is notable, as the very nature of allostery suggests that all parameters are intimately connected, thus enabling binding events at one domain to be “sensed” by another.

With knowledge of how a collection of DNA binding and inducer binding single mutants behave, we predict the induction profiles and the free-energy changes of pairwise double mutants with quantitative accuracy. We find that the energetic effects of each individual mutation are additive, indicating that epistatic interactions are absent between the mutations examined here. Our model provides a means for identifying and quantifying the extent of epistatic interactions in a more complex set of mutations and can shed light on how the protein sequence and general regulatory architecture coevolve.

## Results

**Fig. 1.**
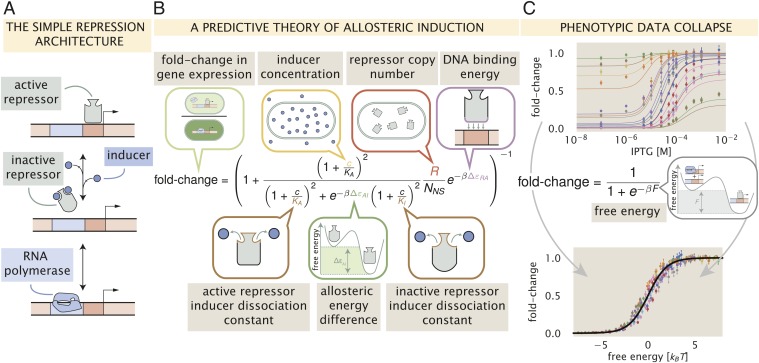
A predictive framework for phenotypic and energetic dissection of the simple repression motif. (*A*) The inducible simple repression architecture. When in the active state, the repressor (gray) binds the cognate operator sequence of the DNA (red box) with high specificity, preventing transcription by occluding binding of the RNA polymerase to the promoter (blue rectangle). Upon addition of an inducer molecule, the inactive state becomes energetically preferable, and the repressor no longer binds the operator sequence with appreciable specificity. Once unbound from the operator, binding of the RNA polymerase (blue) is no longer blocked, and transcription can occur. (*B*) The simple repression input–output function for an allosteric repressor with 2 inducer binding sites. The key parameters are identified in speech bubbles. (*C*) The fold change in gene expression collapses as a function of the free energy. *C*, *Top* shows measurements of the fold change in gene expression as a function of inducer concentration from Razo-Mejia et al. (2018) (10). Points and errors correspond to the mean and SEM of at least 10 biological replicates. The thin lines represent the line of best fit given the model shown in *B*. This model can be rewritten as a Fermi function with an energetic parameter F, which is the energetic difference between the repressor bound and unbound states of the promoter, schematized in *C*, *Middle*. The points in *C*, *Bottom* correspond to the data shown in *C*, *Top* collapsed onto a master curve defined by their calculated free energy F. The solid black line is the master curve defined by the Fermi function shown in *C*, *Middle*.

This work considers the inducible simple repression regulatory motif (depicted in Fig. 1*A*) from a thermodynamic perspective which has been thoroughly dissected and tested experimentally (4, 6, 10). While we direct the reader to *SI Appendix*, *SI Text* for a complete derivation, the result of this extensive theory–experiment dialogue is a succinct input–output function (schematized in Fig. 1*B*) that computes the fold change in gene expression relative to an unregulated promoter. This function is of the form

$$\text{fold}-\text{change}={\left(1+\frac{R_{A}}{N_{NS}}e^{-\beta \Delta \varepsilon_{RA}}\right)}^{-1},$$ [1]

where $R_{A}$ is the number of active repressors per cell, $N_{NS}$ is the number of nonspecific binding sites for the repressor, $\Delta \varepsilon_{RA}$ is the binding energy of the repressor to its specific binding site relative to the nonspecific background, and β is defined as $\frac{1}{k_{B}T}$, where $k_{B}$ is the Boltzmann constant and T is the temperature. While this theory requires knowledge of the number of active repressors, we often only know the total number R, which is the sum total of active and inactive repressors. We can define a prefactor $p_{\text{act}}\left(c\right)$ which captures the allosteric nature of the repressor and encodes the probability that a repressor is in the active (repressive) state rather than the inactive state for a given inducer concentration c—namely,

$$p_{\text{act}}\left(c\right)=\frac{{\left(1+\frac{c}{K_{A}}\right)}^{n}}{{\left(1+\frac{c}{K_{A}}\right)}^{n}+e^{-\beta \Delta \varepsilon_{AI}}{\left(1+\frac{c}{K_{I}}\right)}^{n}}.$$ [2]

Here, $K_{A}$ and $K_{I}$ are the dissociation constants of the inducer to the active and inactive repressor, $\Delta \varepsilon_{AI}$ is the energetic difference between the repressor active and inactive states, and n is the number of allosteric binding sites per repressor molecule (n=2 for LacI). With this in hand, we can define $R_{A}$ in Eq. **1** as $R_{A}=p_{\text{act}}\left(c\right)R$.

A key feature of Eqs. **1** and **2** is that the diverse phenomenology of the gene-expression induction profile can be collapsed onto a single master curve by rewriting the input–output function in terms of the free energy F [also called the Bohr parameter (16)],

$$\text{fold}-\text{change}={\left(1+e^{-\beta F}\right)}^{-1},$$ [3]

where

$$F=-k_{B}T\log p_{\text{act}}\left(c\right)-k_{B}T\log\left(\frac{R}{N_{NS}}\right)+\Delta \varepsilon_{RA}.$$ [4]

Hence, if different combinations of parameters yield the same free energy, they will give rise to the same fold change in gene expression, enabling us to collapse multiple regulatory scenarios onto a single curve. This can be seen in Fig. 1*C*, where 18 unique inducer titration profiles of a LacI simple repression architecture collected and analyzed in Razo-Mejia et al. (2018) (10) collapse onto a single master curve. The tight distribution about this curve reveals that the fold change across a variety of genetically distinct individuals can be adequately described by a small number of parameters. Beyond predicting the induction profiles of different strains, the method of data collapse inspired by Eqs. **3** and **4** can be used as a tool to identify mechanistic changes in the regulatory architecture (14). Similar data-collapse approaches have been used previously in such a manner and have proved vital for distinguishing between changes in parameter values and changes in the fundamental behavior of the system (14, 15).

Assuming that a given mutation does not result in a nonfunctional protein, it is reasonable to say that any or all of the parameters in Eq. **1** can be affected by the mutation, changing the observed induction profile and therefore the free energy. To examine how the free energy of a mutant *F*^(mut)^ differs from that of the wild-type *F*^(wt)^, we define $\Delta F=F^{\left(mut\right)}-F^{\left(wt\right)}$, which has the form

$$\begin{array}{ccc} \Delta F & =-k_{B}T\log\left(\frac{p_{\text{act}}^{\left(mut\right)}\left(c\right)}{p_{\text{act}}^{\left(wt\right)}\left(c\right)}\right) & -k_{B}T\log\left(\frac{R^{\left(mut\right)}}{R^{\left(wt\right)}}\right)\\ & \;+\left(\Delta \varepsilon_{RA}^{\left(mut\right)}-\Delta \varepsilon_{RA}^{\left(wt\right)}\right). \end{array}$$ [5]

**Fig. 2.**
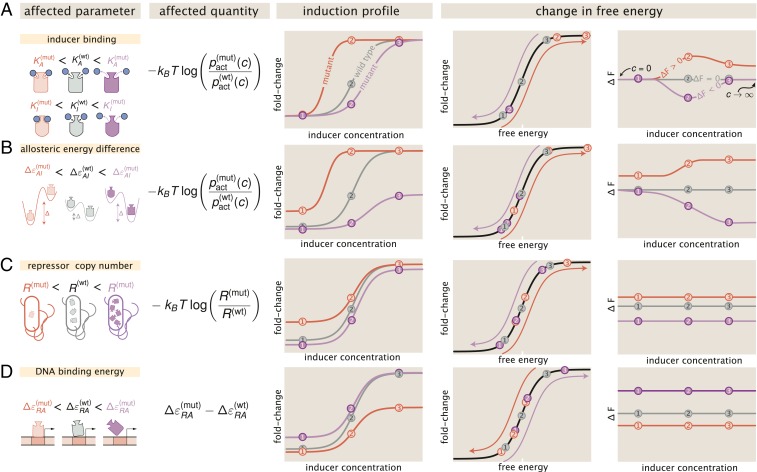
Parametric changes due to mutations and the corresponding free-energy changes for (*A*) perturbations to $K_{a}$ and $K_{i}$, (*B*) changes to the allosteric energy difference ${\Delta \varepsilon }_{AI}$, (*C*) changes to repressor copy number, and (*D*) changes in DNA binding affinity. The 1st column schematizes the changed parameters and the 2nd column reflects which quantity in Eq. **5** is affected. The 3rd column shows representative induction profiles from mutants which have smaller (red) and larger (purple) values for the parameters than the wild type (gray). The 4th and 5th columns illustrate how the free energy is changed as a result. Purple and red arrows indicate the direction in which the points are translated about the master curve. Three concentrations (points labeled 1, 2, and 3) are shown to illustrate how each point is moved in free-energy space. An interactive version of this figure can be found on the paper website (https://www.rpgroup.caltech.edu/mwc_mutants/) (17).

ΔF describes how a mutation translates a point across the master curve shown in Fig. 1*C*. As we will show in the coming paragraphs (illustrated in Fig. 2), this formulation coarse grains the myriad parameters shown in Eqs. **1** and **2** into 3 distinct quantities, each with different sensitivities to parametric changes. By examining how a mutation changes the ΔF as a function of the inducer concentration, one can draw conclusions as to which parameters have been modified based solely on the shape of the curve. To help the reader understand how various perturbations to the parameters tune the free energy, we have hosted an interactive figure on the dedicated paper website (https://www.rpgroup.caltech.edu/mwc_mutants/) which makes exploration of parameter space a simpler task (17).

The first term in Eq. **5** is the log ratio of the probability of a mutant repressor being active relative to the wild type at a given inducer concentration c. This quantity defines how changes to any of the allosteric parameters—such as inducer binding constants $K_{A}$ and $K_{I}$ or active/inactive state energetic difference $\Delta \varepsilon_{AI}$—alter the free energy F, which can be interpreted as the free-energy difference between the repressor-bound and -unbound states of the promoter. Fig. 2*A* illustrates how changes to the inducer binding constants $K_{A}$ and $K_{I}$ alone alter the induction profiles and resulting free energy as a function of the inducer concentration. In the limit where c=0, the values of $K_{A}$ and $K_{I}$ do not factor into the calculation of $p_{\text{act}}\left(c\right)$ given by Eq. **2**, meaning that $\Delta \varepsilon_{AI}$ is the lone parameter setting the residual activity of the repressor. Thus, if only $K_{A}$ and $K_{I}$ are altered by a mutation, then ΔF should be $0\;k_{B}T$ when c=0, illustrated by the overlapping red, purple, and gray curves in the right-hand plot of Fig. 2*A*. However, if $\Delta \varepsilon_{AI}$ is influenced by the mutation (either alone or in conjunction with $K_{A}$ and $K_{I}$), the leakiness will change, resulting in a nonzero ΔF when c=0. This is illustrated in Fig. 2*B*, where $\Delta \varepsilon_{AI}$ is the only parameter affected by the mutation.

It is important to note that for a mutation which perturbs only the inducer binding constants, the dependence of ΔF on the inducer concentration can be nonmonotonic. While the precise values of $K_{A}$ and $K_{I}$ control the sensitivity of the repressor to inducer concentration, it is the ratio $K_{A}/K_{I}$ that defines whether this nonmonotonic behavior is observed. This can be seen more clearly when we consider the limit of saturating inducer concentration,

$$\underset{c\to \infty }{\lim}\log\left(\frac{p_{\text{act}}^{\left(mut\right)}}{p_{\text{act}}^{\left(wt\right)}}\right)\approx \log\left[\frac{1+e^{-\beta \Delta \varepsilon_{AI}^{\left(wt\right)}}{\left(\frac{K_{A}^{\left(wt\right)}}{K_{I}^{\left(wt\right)}}\right)}^{n}}{1+e^{-\beta \Delta \varepsilon_{AI}^{\left(wt\right)}}{\left(\frac{K_{A}^{\left(mut\right)}}{K_{I}^{\left(mut\right)}}\right)}^{n}}\right],$$ [6]

which illustrates that ΔF returns to zero at saturating inducer concentration when the ratio $K_{A}/K_{I}$ is the same for both the mutant and wild-type repressors, so long as $\Delta \varepsilon_{AI}$ is unperturbed. Nonmonotonicity can only be achieved by changing $K_{A}$ and $K_{I}$ and therefore serves as a diagnostic for classifying mutational effects reliant solely on measuring the change in free energy. A rigorous proof of this nonmonotonic behavior given changing $K_{A}$ and $K_{I}$ can be found in *SI Appendix*, *SI Text*.

The second term in Eq. **5** captures how changes in the repressor copy number contribute to changes in free energy. It is important to note that this contribution to the free-energy change depends on the total number of repressors in the cell, not just those in the active state. This emphasizes that changes in the expression of the repressor are energetically divorced from changes to the allosteric nature of the repressor. As a consequence, the change in free energy is constant for all inducer concentrations, as is schematized in Fig. 2*C*. Because the magnitude of the change in free energy scales logarithmically with changing repressor copy number, a mutation which increases expression from 1 to 10 repressors per cell is more impactful from an energetic standpoint ($k_{B}T\log\left(10/1\right)\approx 2.3\;k_{B}T$) than an increase from 90 to 100 ($k_{B}T\log\left(100/90\right)\approx 0.1\;k_{B}T$). Appreciable changes in the free energy only arise when variations in the repressor copy number are larger than or comparable to an order of magnitude. Changes of this magnitude are certainly possible from a single point mutation, as it has been shown that even synonymous substitutions can drastically change translation efficiency (18).

The third and final term in Eq. **5** is the difference in the DNA binding energy between the mutant and wild-type repressors. All else being equal, if the mutated state binds more tightly to the DNA than the wild type ($\Delta \varepsilon_{RA}^{\left(wt\right)}>\Delta \varepsilon_{RA}^{\left(mut\right)}$), the net change in the free energy is negative, indicating that the repressor-bound states become more energetically favorable due to the mutation. Much like in the case of changing repressor copy number, this quantity is independent of inducer concentration and is therefore also constant (Fig. 2*D*). However, the magnitude of the change in free energy is linear with DNA binding affinity, while it is logarithmic with respect to changes in the repressor copy number. Thus, to change the free energy by $1\;k_{B}T$, the repressor copy number must change by a factor of ≈2.3, whereas the DNA binding energy must change by $1\;k_{B}T$.

The unique behavior of each quantity in Eq. **5** and its sensitivity with respect to the parameters makes ΔF useful as a diagnostic tool to classify mutations. Given a set of fold-change measurements, a simple rearrangement of Eq. **3** permits the direct calculation of the free energy, assuming that the underlying physics of the regulatory architecture has not changed. Thus, it becomes possible to experimentally test the general assertions made in Fig. 2.

### DNA Binding Domain Mutations.

With this arsenal of analytic diagnostics, we can begin to explore the mutational space of the repressor and map these mutations to the biophysical parameters they control. As one of the most thoroughly studied transcription factors, LacI has been subjected to numerous crystallographic and mutational studies (19–22). One such work generated a set of point mutations in the LacI repressor and examined the diversity of the phenotypic response to different allosteric effectors (5). However, several experimental variables were unknown, precluding precise calculation of ΔF as presented in the previous section. In ref. 5, the repressor variants and the fluorescence reporter were expressed from separate plasmids. As the copy numbers of these plasmids fluctuate in the population, both the population average repressor copy number and the number of regulated promoters were unknown. Both of these quantities have been shown to significantly alter the measured gene expression, and calculation of ΔF is dependent on knowledge of their values. While the approach presented in ref. 5 considers the Lac repressor as an Monod–Wyman–Changeux (MWC) molecule, the copy numbers of the repressor and the reporter gene were swept into an effective parameter $\frac{R}{K_{DNA}}$, hindering our ability to distinguish between changes in repressor copy number or in DNA binding energy. To test our hypothesis of free-energy differences resulting from various parameter perturbations, we used the dataset in ref. 5 as a guide and chose a subset of the mutations to quantitatively dissect. To control copy-number variation, the mutant repressors and the reporter gene were integrated into the *E. coli* chromosome, where the copy numbers are known and tightly controlled (4, 10). Furthermore, the mutations were paired with ribosomal binding sites where the level of translation of the wild-type repressor had been directly measured (4).

We made 3 amino acid substitutions (Y17I, Q18A, and Q18M) that are critical for the DNA–repressor interaction. These mutations were introduced into the *lacI* sequence used in Garcia and Phillips (2011) (4) with 4 different ribosomal binding site sequences that were shown (via quantitative Western blotting) to tune the wild-type repressor copy number across 3 orders of magnitude. These mutant constructs were integrated into the *E. coli* chromosome harboring a yellow fluorescent protein (YFP) reporter. The YFP promoter included the native O2 LacI operator sequence, which the wild-type LacI repressor binds with high specificity ($\Delta \varepsilon_{RA}=-13.9\;k_{B}T$). The fold change in gene expression for each mutant across 12 concentrations of isopropyl β-d-thiogalactopyranoside (IPTG) was measured via flow cytometry (23). As we mutated only a single amino acid with the minimum number of base-pair changes to the codons from the wild-type sequence, we find it unlikely that the repressor copy number was drastically altered from those reported in ref. 4 for the wild-type sequence paired with the same ribosomal binding site sequence. In characterizing the effects of these DNA binding mutations, we take the repressor copy number to be unchanged. Any error introduced by this assumption should be manifest as a larger-than-predicted systematic shift in the free-energy change when the repressor copy number is varied.

**Fig. 3.**
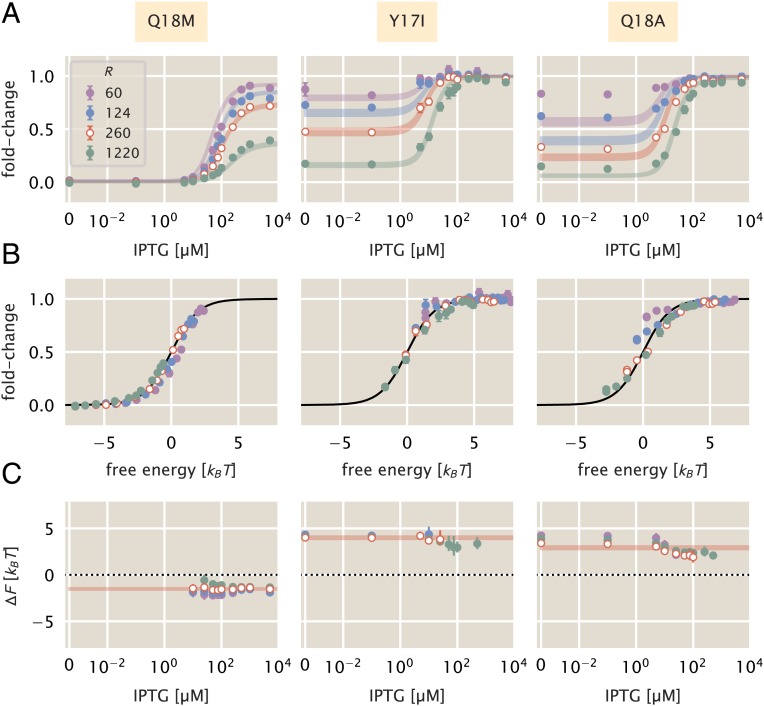
Induction profiles and free-energy differences of DNA binding domain mutations. Each column corresponds to the highlighted mutant at the top of the figure. Each strain was paired with the native O2 operator sequence. Open points correspond to the strain for each mutant from which the DNA binding energy was estimated. (*A*) Induction profiles of each mutant at 4 different repressor copy numbers as a function of the inducer concentration. Points correspond to the mean fold change in gene expression of 6–10 biological replicates. Error bars are the SEM. Shaded regions demarcate the 95% credible region of the induction profile generated by the estimated DNA binding energy. (*B*) Data collapse of all points for each mutant shown in *A* using only the DNA binding energy estimated from a single repressor copy number. Points correspond to the average fold change in gene expression of 6–10 biological replicates. Error bars are SEM. Where error bars are not visible, the relative error in measurement is smaller than the size of the marker. (*C*) The change in the free energy resulting from each mutation as a function of the inducer concentration. Points correspond to the median of the marginal posterior distribution for the free energy. Error bars represent the upper and lower bounds of the 95% credible region. Points in *A* at the detection limits of the flow cytometer (near fold-change values of 0 and 1) were neglected for calculation of the ΔF. The IPTG concentration is shown on a symmetric log scale with linear scaling ranging from 0 to $10^{-2}\;\mu$M and log scaling elsewhere. The shaded red lines in *C* correspond to the 95% credible region of our predictions for ΔF based solely on estimation of $\Delta \varepsilon_{RA}$ from the strain with R=260 repressors per cell.

A naïve hypothesis for the effect of a mutation in the DNA binding domain is that only the DNA binding energy is affected. This hypothesis appears to contradict the core principle of allostery in that ligand binding in one domain influences binding in another, suggesting that changing any parameter modifies them all. The characteristic curves summarized in Fig. 2 give a means to discriminate between these 2 hypotheses by examining the change in the free energy. Using a single induction profile (open points in Fig. 3), we estimated the DNA binding energy using Bayesian inferential methods, the details of which are thoroughly discussed in *Materials and Methods* as well as *SI Appendix*, *SI Text*. The shaded red region for each mutant in Fig. 3 represents the 95% credible region of this fit, whereas all other shaded regions are 95% credible regions of the predictions for other repressor copy numbers. We find that redetermining only the DNA binding energy accurately captures the majority of the induction profiles, indicating that other parameters are unaffected. One exception is for the lowest repressor copy numbers (R=60 and R=124 per cell) of mutant Q18A at low concentrations of IPTG. However, we note that this disagreement is comparable to that observed for the wild-type repressor binding to the weakest operator in Razo-Mejia et al. (2018) (10), illustrating that our model is imperfect in characterizing weakly repressing architectures. Including other parameters in the fit (such as $\Delta \varepsilon_{AI}$) does not significantly improve the accuracy of the predictions. Furthermore, the magnitude of this disagreement also depends on the choice of the fitting strain (*SI Appendix*, *SI Text*).

Mutations Y17I and Q18A both weaken the affinity of the repressor to the DNA relative to the wild-type strain with binding energies of $-9.9_{-0.1}^{+0.1}\;k_{B}T$ and $-11.0_{-0.1}^{+0.1}\;k_{B}T$, respectively. Here, we report the median of the inferred posterior probability distribution with the superscripts and subscripts corresponding to the upper and lower bounds of the 95% credible region. These binding energies are comparable to that of the wild-type repressor affinity to the native LacI operator sequence O3, with a DNA binding energy of $-9.7\;k_{B}T$. The mutation Q18M increases the strength of the DNA–repressor interaction relative to the wild-type repressor with a binding energy of $-15.43_{-0.06}^{+0.07}\;k_{B}T$, comparable to the affinity of the wild-type repressor to the native O1 operator sequence ($-15.3\;k_{B}T$). It is notable that a single amino acid substitution of the repressor is capable of changing the strength of the DNA binding interaction well beyond that of many single base-pair mutations in the operator sequence (4, 24).

Using the new DNA binding energies, we can collapse all measurements of fold change as a function of the free energy, as shown in Fig. 3*B*. This allows us to test the diagnostic power of the decomposition of the free energy described in Fig. 2. To compute the ΔF for each mutation, we inferred the observed mean free energy of the mutant strain for each inducer concentration and repressor copy number (see *Materials and Methods* as well as *SI Appendix*, *SI Text* for a detailed explanation of the inference). We note that in the limit of extremely low or high fold change, the inference of the free energy is either overestimated or underestimated, respectively, introducing a systematic error. Thus, points which are close to these limits are omitted in the calculation of ΔF. We direct the reader to *SI Appendix*, *SI Text* for a detailed discussion of this systematic error. With a measure of *F*^(mut)^ for each mutant at each repressor copy number, we compute the difference in free energy relative to the wild-type strain with the same repressor copy number and operator sequence, restricting all variability in ΔF solely to changes in $\Delta \varepsilon_{RA}$.

The change in free energy for each mutant is shown in Fig. 3*C*. It can be seen that the ΔF for each mutant is constant as a function of the inducer concentration and is concordant with the prediction generated from fitting $\Delta \varepsilon_{RA}$ to a single repressor copy number (red lines Fig. 3*C*). This is in line with the predictions outlined in Fig. 2 *C* and *D*, indicating that the allosteric parameters are “insulated,” meaning that they are not affected by the DNA binding domain mutations. As the ΔF for all repressor copy numbers collapses onto the prediction, we can say that the expression of the repressor itself is the same or comparable with that of the wild type. If the repressor copy number were perturbed in addition to $\Delta \varepsilon_{RA}$, one would expect a shift away from the prediction that scales logarithmically with the change in repressor copy number. However, as the ΔF is approximately the same for each repressor copy number, it can be surmised that the mutation does not significantly change the expression or folding efficiency of the repressor itself. These results allow us to state that the DNA binding energy $\Delta \varepsilon_{RA}$ is the only parameter modified by the DNA mutants examined.

### Inducer Binding Domain Mutations.

Much as in the case of the DNA binding mutants, we cannot safely assume a priori that a given mutation in the inducer binding domain affects only the inducer binding constants $K_{A}$ and $K_{I}$. While it is easy to associate the inducer binding constants with the inducer binding domain, the critical parameter in our allosteric model $\Delta \varepsilon_{AI}$ is harder to restrict to a single spatial region of the protein. As $K_{A}$, $K_{I}$, and $\Delta \varepsilon_{AI}$ are all parameters dictating the allosteric response, we consider 2 hypotheses in which inducer binding mutations alter either all 3 parameters or only $K_{A}$ and $K_{I}$.

We made 4 point mutations within the inducer binding domain of LacI (F161T, Q291V, Q291R, and Q291K) that have been shown to alter binding to multiple allosteric effectors (5). In contrast to the DNA binding domain mutants, we paired the inducer binding domain mutations with the 3 native LacI operator sequences (which have various affinities for the repressor) and a single ribosomal binding site sequence. This ribosomal binding site sequence, as reported in ref. 4, expresses the wild-type LacI repressor to an average copy number of ∼260 per cell. As the free-energy differences resulting from point mutations in the DNA binding domain can be described solely by changes to $\Delta \varepsilon_{RA}$, we continue under the assumption that the inducer binding domain mutations do not significantly alter the repressor copy number.

**Fig. 4.**
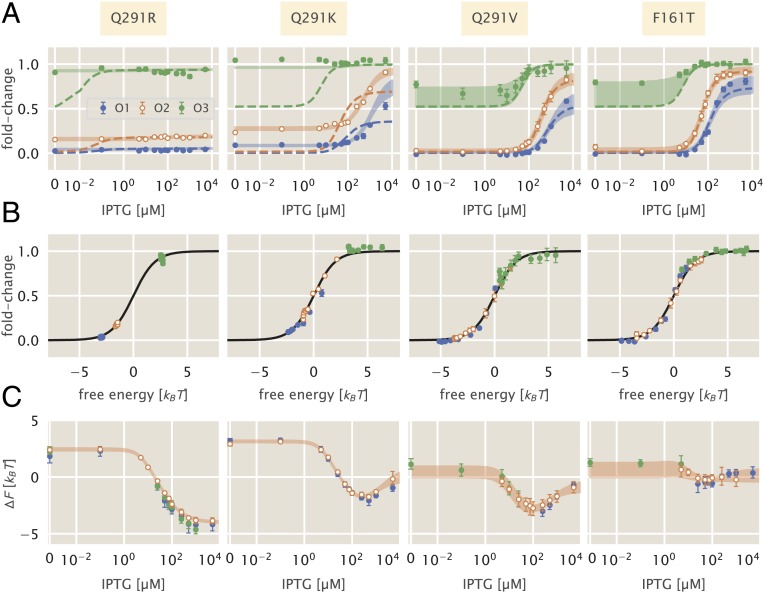
Induction profiles and free-energy differences of inducer binding domain mutants. Open points represent the strain to which the parameters were fit—namely, the O2 operator sequence. Each column corresponds to the mutant highlighted at the top of the figure. All strains have R=260 per cell. (*A*) The fold change in gene expression as a function of the inducer concentration for 3 operator sequences of varying strength. Dashed lines correspond to the curve of best fit resulting from fitting $K_{A}$ and $K_{I}$ alone. Shaded curves correspond to the 95% credible region of the induction profile determined from fitting $K_{A}$, $K_{I}$, and $\Delta \varepsilon_{AI}$. Points correspond to the mean measurement of 6–12 biological replicates. Error bars are the SEM. (*B*) Points in *A* collapsed as a function of the free energy calculated from redetermining $K_{A}$, $K_{I}$, and $\Delta \varepsilon_{AI}$. (*C*) Change in free energy resulting from each mutation as a function of the inducer concentration. Points correspond to the median of the posterior distribution for the free energy. Error bars represent the upper and lower bounds of the 95% credible region. Shaded curves are the predictions. IPTG concentration is shown on a symmetric log scaling axis with the linear region spanning from 0 to $10^{-2}\;\mu$M and log scaling elsewhere.

The induction profiles for these 4 mutants are shown in Fig. 4*A*. Of the mutations chosen, Q291R and Q291K appear to have the most significant impact, with Q291R abolishing the characteristic sigmoidal titration curve entirely. It is notable that both Q291R and Q291K have elevated expression in the absence of inducer compared to the other 2 mutants paired with the same operator sequence. Fig. 2*A* illustrates that if only $K_{A}$ and $K_{I}$ were being affected by the mutations, the fold change should be identical for all mutants in the absence of inducer. This discrepancy in the observed leakiness immediately suggests that more than $K_{A}$ and $K_{I}$ are affected for Q291K and Q291R.

Using a single induction profile for each mutant (shown in Fig. 4 as open circles), we inferred the parameter combinations for both hypotheses and drew predictions for the induction profiles with other operator sequences. We found that the simplest hypothesis (in which only $K_{A}$ and $K_{I}$ are altered) does not permit accurate prediction of most induction profiles. These curves, shown as dotted lines in Fig. 4*A*, failed spectacularly in the case of Q291R and Q291K and undershot the observed profiles for F161T and Q291V, especially when paired with the weak operator sequence O3. The change in the leakiness for Q291R and Q291K is particularly evident, as the expression at c=0 should be identical to the wild-type repressor under this hypothesis. Altering only $K_{A}$ and $K_{I}$ is not sufficient to accurately predict the induction profiles for F161T and Q291V, but not to the same degree as Q291K and Q291R. The disagreement is most evident for the weakest operator O3 (green lines in Fig. 4*A*), although we have discussed previously that the induction profiles for weak operators are difficult to accurately describe and can result in comparable disagreement for the wild-type repressor (10, 24).

Including $\Delta \varepsilon_{AI}$ as a perturbed parameter in addition to $K_{A}$ and $K_{I}$ improved the predicted profiles for all 4 mutants. By fitting these 3 parameters to a single strain, we were able to accurately predict the induction profiles of other operators, as shown by the shaded lines in Fig. 4*A*. With these modified parameters, all experimental measurements collapsed as a function of their free energy as prescribed by Eq. **3** (Fig. 4*B*). All 4 mutations significantly diminished the binding affinity of both states of the repressor to the inducer, as shown by the estimated parameter values reported in Table 1. As evident in the data alone, Q291R abrogated inducibility outright ($K_{A}\approx K_{I}$). For Q291K, the active state of the repressor can no longer bind inducer, whereas the inactive state binds with weak affinity. The remaining 2 mutants, Q291V and F161T, both showed diminished binding affinity of the inducer to both the active and inactive states of the repressor relative to the wild type.

**Table 1.** Inferred values of $K_{A}$, $K_{I}$, and $\Delta \varepsilon_{AI}$ for inducer binding mutants

Mutant	$K_{A}$	$K_{I}$	$\Delta \varepsilon_{AI}$ [$k_{B}T$]	Reference
Wild type	$139_{-22}^{+29}\;\mu$M	$0.53_{-0.04}^{+0.04}\;\mu$M	4.5	(10)
F161T	$165_{-65}^{+90}\;\mu$M	$3_{-3}^{+6}\;\mu$M	$1_{-2}^{+5}$	This study
Q291V	$650_{-250}^{+450}\;\mu$M	$8_{-8}^{+8}\;\mu$M	$3_{-3}^{+6}$	This study
Q291K	>1 mM	$310_{-60}^{+70}\;\mu$M	$-3.11_{-0.07}^{+0.07}$	This study
Q291R	$9_{-9}^{+20}\;\mu$M	$8_{-8}^{+20}\;\mu$M	$-2.35_{-0.09}^{+0.01}$	This study


Given the collection of fold-change measurements, we computed the ΔF relative to the wild-type strain with the same operator and repressor copy number. This leaves differences in $p_{act}\left(c\right)$ as the sole contributor to the free-energy difference, assuming our hypothesis that $K_{A}$, $K_{I}$, and $\Delta \varepsilon_{AI}$ are the only perturbed parameters is correct. The change in free energy can be seen in Fig. 4*C*. For all mutants, the free-energy difference inferred from the observed fold-change measurements falls within error of the predictions generated under the hypothesis that $K_{A}$, $K_{I}$, and $\Delta \varepsilon_{AI}$ are all affected by the mutation (shaded curves in Fig. 4*C*). The profile of the free-energy change exhibits some of the rich phenomenology illustrated in Fig. 2 *A* and *B*. Q291K, F161T, and Q291V exhibited a nonmonotonic dependence on the inducer concentration, a feature that can only appear when $K_{A}$ and $K_{I}$ are altered. The nonzero ΔF at c=0 for Q291R and Q291K coupled with an inducer concentration dependence is a telling sign that $\Delta \varepsilon_{AI}$ must be significantly modified. This shift in ΔF was positive in all cases, indicating that $\Delta \varepsilon_{AI}$ must have decreased and that the inactive state had become more energetically favorable for these mutants than for the wild-type protein. Indeed, the estimates for $\Delta \varepsilon_{AI}$ (Table 1) reveal that both mutations Q291R and Q291K make the inactive state more favorable than the active state. Thus, for these 2 mutations, only ≈10% of the repressors are active in the absence of inducer, whereas the basal active fraction is ≈99% for the wild-type repressor (10). We note that the parameter values reported here disagree with those reported in ref. 5. This disagreement stems from different assumptions regarding the residual activity of the repressor in the absence of inducer and the parametric degeneracy of the MWC model without a concrete independent measure of $\Delta \varepsilon_{AI}$. A detailed discussion of the difference in parameter values between our previous work (10), that of Daber et al. (2011) (5), and those of other seminal works (25, 26) can be found in *SI Appendix*, *SI Text*.

Taken together, these parametric changes diminish the response of the regulatory architecture as a whole to changing inducer concentrations. They furthermore reveal that the parameters which govern the allosteric response are interdependent, and no single parameter is insulated from the others. However, as only the allosteric parameters are changed, one can say that the allosteric parameters as a whole are insulated from the other components which define the regulatory response, such as repressor copy number and DNA binding affinity.

### Predicting Effects of Pairwise Double Mutations.

Given full knowledge of each individual mutation, we can draw predictions of the behavior of the pairwise double mutants with no free parameters based on the simplest null hypothesis of no epistasis. The formalism of ΔF defined by Eq. **5** explicitly states that the contribution to the free energy of the system from the difference in DNA binding energy and the allosteric parameters are strictly additive. Thus, deviations from the predicted change in free energy would suggest epistatic interactions between the 2 mutations.

**Fig. 5.**
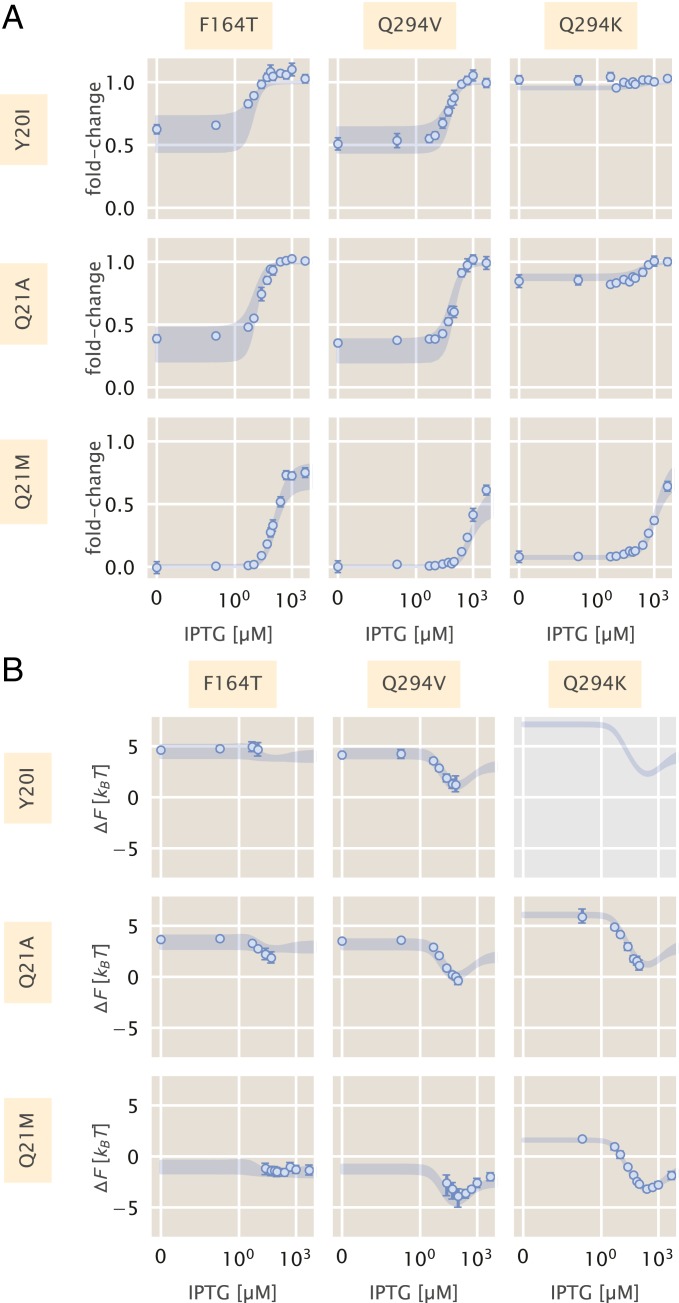
Induction and free-energy profiles of DNA binding and inducer binding double mutants. (*A*) Fold change in gene expression for each double mutant as a function of IPTG. Points and errors correspond to the mean and SE of 6–10 biological replicates. Where not visible, error bars are smaller than the corresponding marker. Shaded regions correspond to the 95% credible region of the prediction given knowledge of the single mutants. These were generated by drawing $10^{4}$ samples from the $\Delta \varepsilon_{RA}$ posterior distribution of the single DNA binding domain mutants and the joint probability distribution of $K_{A}$, $K_{I}$, and $\Delta \varepsilon_{AI}$ from the single inducer binding domain mutants. (*B*) The difference in free energy of each double mutant as a function of the reference free energy. Points and errors correspond to the median and bounds of the 95% credible region of the posterior distribution for the inferred ΔF. Shaded lines region are the predicted change in free energy, generated in the same manner as the shaded lines in *A*. All measurements were taken from a strain with 260 repressors per cell paired with a reporter with the native O2 LacI operator sequence. In all plots, the IPTG concentration is shown on a symmetric log axis with linear scaling between 0 and $10^{-2}\;\mu$M and log scaling elsewhere.

To test this additive model, we constructed 9 double-mutant strains, each having a unique inducer binding (F161T, Q291V, and Q291K) and DNA binding mutation (Y17I, Q18A, and Q18M). To make predictions with an appropriate representation of the uncertainty, we computed a large array of induction profiles given random draws from the posterior distribution for the DNA binding energy (determined from the single DNA binding mutants) as well as from the joint posterior for the allosteric parameters (determined from the single inducer binding mutants). These predictions, shown in Fig. 5 as shaded blue curves, capture all experimental measurements of the fold change (Fig. 5*A*) and the inferred difference in free energy (Fig. 5*B*). The latter indicates that there are no epistatic interactions between the mutations queried in this work, although if there were, systematic deviations from these predictions would shed light on how the epistasis is manifest.

The precise agreement between the predictions and measurements for Q291K paired with either Q18A or Q18M is striking, as Q291K drastically changed $\Delta \varepsilon_{AI}$ in addition to $K_{A}$ and $K_{I}$. Our ability to predict the induction profile and free-energy change underscores the extent to which the DNA binding energy and the allosteric parameters are insulated from one another. Despite this insulation, the repressor still functions as an allosteric molecule, emphasizing that the mutations we have inserted do not alter the pathway of communication between the 2 domains of the protein. As the double mutant Y17I–Q291K exhibits fold change of ∼1 across all IPTG concentrations (Fig. 5*A*), these mutations in tandem make repression so weak that it is beyond the limits which are detectable by our experiments. As a consequence, we were unable to estimate ΔF or experimentally verify the corresponding prediction (gray box in Fig. 5*B*). However, as the predicted fold change in gene expression is also ∼1 for all c, we believe that the prediction shown for ΔF is likely accurate. One would be able to infer the ΔF to confirm these predictions using a more sensitive method for measuring the fold change, such as single-cell microscopy or colorimetric assays.

## Discussion

Allosteric regulation is often couched as “biological action at a distance.” Despite extensive knowledge of protein structure and function, it remains difficult to translate the coordinates of the atomic constituents of a protein to the precise parameter values which define the functional response, making each mutant its own intellectual adventure. Bioinformatic approaches to understanding the sequence–structure relationship have permitted us to examine how the residues of allosteric proteins evolve, revealing conserved regions which hint to their function. Coevolving residues reveal sectors of conserved interactions which traverse the protein that act as the allosteric communication channel between domains (27–29). Elucidating these sectors has advanced our understanding of how distinct domains “talk” to one another and has permitted direct engineering of allosteric responses into nonallosteric enzymes (30–32). Even so, we are left without a quantitative understanding of how these admittedly complex networks set the energetic difference between active and inactive states or how a given mutation influences binding affinity. In this context, a biophysical model in which the various parameters are intimately connected to the molecular details can be of use and can lead to quantitative predictions of the interplay between amino acid identity and system-level response.

By considering how each parameter contributes to the observed change in free energy, we are able to tease out different classes of parameter perturbations which result in stereotyped responses to changing inducer concentration. These characteristic changes to the free energy can be used as a diagnostic tool to classify mutational effects. For example, we show in Fig. 2 that modulating the inducer binding constants $K_{A}$ and $K_{I}$ results in nonmonotonic free-energy changes that are dependent on the inducer concentration, a feature observed in the inducer binding mutants examined in this work. Simply looking at the inferred ΔF as a function of inducer concentration, which requires no fitting of the biophysical parameters, indicates that $K_{A}$ and $K_{I}$ must be modified, considering that those are the only parameters which can generate such a response.

Another key observation is that a perturbation to only $K_{A}$ and $K_{I}$ requires that the ΔF=0 at c=0. Deviations from this condition imply that more than the inducer binding constants must have changed. If this shift in ΔF off of 0 at c=0 is not constant across all inducer concentrations, we can surmise that the energy difference between the allosteric states $\Delta \varepsilon_{AI}$ must also be modified. We again see this effect for all of our inducer mutants. By examining the inferred ΔF, we can immediately say that, in addition to $K_{A}$ and $K_{I}$, $\Delta \varepsilon_{AI}$ must decrease relative to the wild-type value as ΔF>0 at c=0. When the allosteric parameters are fit to the induction profiles, we indeed see that this is the case, with all 4 mutations decreasing the energy gap between the active and inactive states. Two of these mutations, Q291R and Q291K, make the inactive state of the repressor more stable than the active state, which is not the case for the wild-type repressor (10).

Our formulation of ΔF indicates that shifts away from 0 that are independent of the inducer concentration can only arise from changes to the repressor copy number and/or DNA binding specificity, indicating that the allosteric parameters are untouched. We see that for 3 mutations in the DNA binding domain, ΔF is the same irrespective of the inducer concentration. Measurements of ΔF for these mutants with repressor copy numbers across 3 orders of magnitude yield approximately the same value, revealing that $\Delta \varepsilon_{RA}$ is the sole parameter altered via the mutations.

We note that the conclusions stated above can be qualitatively drawn without resorting to fitting various parameters and measuring the goodness of fit. Rather, the distinct behavior of ΔF is sufficient to determine which parameters are changing. Here, these conclusions are quantitatively confirmed by fitting these parameters to the induction profile, which results in accurate predictions of the fold change and ΔF for nearly every strain across different mutations, repressor copy numbers, and operator sequence, all at different inducer concentrations. With a collection of evidence as to what parameters are changing for single mutations, we put our model to the test and drew predictions of how double mutants would behave both in terms of the titration curve and free-energy profile.

A hypothesis that arises from our formulation of ΔF is that a simple summation of the energetic contribution of each mutation should be sufficient to predict the double mutants (so long as they are in separate domains). We find that such a calculation permits precise and accurate predictions of the double-mutant phenotypes, indicating that there are no epistatic interactions between the mutations examined in this work. With an expectation of what the free-energy differences should be, epistatic interactions could be understood by looking at how the measurements deviate from the prediction. For example, if epistatic interactions exist which appear as a systematic shift from the predicted ΔF independent of inducer concentration, one could conclude that DNA binding energy is not equal to that of the single mutation in the DNA binding domain alone. Similarly, systematic shifts that are dependent on the inducer concentration (i.e., not constant) indicate that the allosteric parameters must be influenced. If the expected difference in free energy is equal to 0 when c=0, one could surmise that the modified parameter must not be $\Delta \varepsilon_{AI}$ or $\Delta \varepsilon_{RA}$, as these would both result in a shift in leakiness, indicating that $K_{A}$ and $K_{I}$ are further modified.

**Fig. 6.**
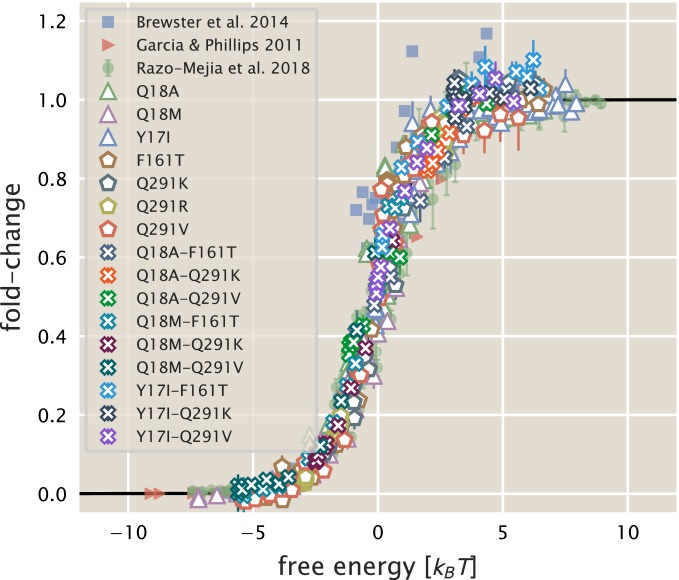
Data collapse of the simple repression regulatory architecture. All data are means of biological replicates. Where present, error bars correspond to the SEM of 5–15 biological replicates. Red triangles indicate data from Garcia and Phillips (4) obtained by colorimetric assays. Blue squares are data from Brewster et al. (6) acquired from video microscopy. Green circles are data from Razo-Mejia et al. (10) obtained via flow cytometry. All other symbols correspond to the work presented here. An interactive version of this figure can be found on the paper website (https://www.rpgroup.caltech.edu/mwc_mutants/), where the different datasets can be viewed in more detail (17).

Ultimately, we present this work as a proof-of-principle for using biophysical models to investigate how mutations influence the response of allosteric systems. We emphasize that such a treatment allows one to boil down the complex phenotypic responses of these systems to a single-parameter description which is easily interpretable as a free energy. The general utility of this approach is illustrated in Fig. 6, where gene-expression data from previous work (4, 6, 10) along with all of the measurements presented in this work collapse onto the master curve defined by Eq. **3**. While our model coarse grains many of the intricate details of transcriptional regulation into 2 states (1 in which the repressor is bound to the promoter and 1 where it is not), it is sufficient to describe a swath of regulatory scenarios. As discussed in *SI Appendix*, *SI Text*, any architecture in which the transcription-factor-bound and transcriptionally active states of the promoter can be separated into 2 distinct coarse-grained states can be subjected to such an analysis.

Given enough parametric knowledge of the system, it becomes possible to examine how modifications to the parameters move the physiological response along this reduced 1-dimensional parameter space. This approach offers a glimpse at how mutational effects can be described in terms of energy rather than Hill coefficients and arbitrary prefactors. While we have explored a very small region of sequence space in this work, coupling of this approach with high-throughput sequencing-based methods to query a library of mutations within the protein will shed light on the phenotypic landscape centered at the wild-type sequence. Furthermore, pairing libraries of protein and operator sequence mutants will provide insight as to how the protein and regulatory sequence coevolve, a topic rich with opportunity for a dialogue between theory and experiment.

## Materials and Methods

### Bacterial Strains and DNA Constructs.

All wild-type strains from which the mutants were derived were generated in work from the Phillips group (4, 10). Briefly, mutations were first introduced into the *lacI* gene of our pZS3*1-lacI plasmid (4) by using a combination of overhang PCR Gibson assembly as well as QuikChange mutagenesis (Agilent Technologies). The oligonucleotide sequences used to generate each mutant as well as the method are provided in *SI Appendix*, *SI Text*.

For mutants generated through overhang PCR and Gibson assembly, oligonucleotide primers were purchased containing an overhang with the desired mutation and used to amplify the entire plasmid. By using the homology of the primer overhang, Gibson assembly was performed to circularize the DNA prior to electroporation into MG1655 *E. coli* cells. Integration of LacI mutants was performed with λ Red recombineering (33) as described in refs. 4 and 33.

The mutants studied in this work were chosen from data reported in ref. 5. In selecting mutations, we looked for mutants which suggested moderate to strong deviations from the behavior of the wild-type repressor. We note that the variant of LacI used in this work has an additional 3 amino acids (Met–Val–Asn) added to the N terminus than the canonical LacI sequence reported in ref. 34. To remain consistent with the field, we have identified the mutations with respect to their positions in the canonical sequence and those in ref. 5. However, their positions in the raw data files correspond to that of our LacI variant and are noted in the README files associated with the data.

### Flow Cytometry.

All fold-change measurements were performed on a MACSQuant flow cytometer as described in Razo-Mejia et al. (10). Briefly, saturated overnight cultures 500 μL in volume were grown in deep-well 96-well plates covered with a breathable nylon cover (Laboratory Pak–Nitex Nylon, Sefar America, catalog no. 241205). After ∼12–15 h, the cultures reached saturation and were diluted 1,000-fold into a second 2-mL 96-deep-well plate where each well contained 500 μL of M9 minimal medium supplemented with 0.5% (wt/vol) glucose (anhydrous d-glucose, Macron Chemicals) and the appropriate concentration of IPTG (dioxane-free, Research Products International). These were sealed with a breathable cover and were allowed to grow for ∼8 h until the ${OD}_{600\text{nm}}\approx 0.3$. Cells were then diluted 10-fold into a round-bottom 96-well plate (Corning catalog no. 3365) containing 90 μL of M9 minimal medium supplemented with 0.5% (wt/vol) glucose along with the corresponding IPTG concentrations.

The flow cytometer was calibrated prior to use with MACSQuant Calibration Beads (catalog no. 130-093-607). During measurement, the cultures were held at ∼4 °C by placing the 96-well plate on a MACSQuant ice block. All fluorescence measurements were made by using a 488-nm excitation wavelength with a 525/50-nm emission filter. The photomultiplier tube voltage settings for the instrument were the same as those used in ref. 10.

The data were processed by using an automatic unsupervised gating procedure based on fitting a 2D Gaussian function to the $\log_{10}$ forward-scattering and the $\log_{10}$ side-scattering data, as described in ref. 10. We considered data points that fell within 40% of the highest density region of the 2D Gaussian function as single-cell measurements. We direct the reader to ref. 10 for further detail and comparison of flow cytometry with single-cell microscopy.

### Bayesian Parameter Estimation.

We used a Bayesian definition of probability in the statistical analysis of all mutants in this work. We direct the reader to *SI Appendix*, *SI Text* for a more detailed summary of the approach, outlining each statistical model in detail, as well as a variety of diagnostic tests. In short, we defined a Gaussian likelihood function for our parameter(s) of interest. Our prior choices varied depending on the parameter(s) of interest, and all choices were thoroughly tested, as is described in *SI Appendix*, *SI Text*. All statistical modeling and parameter inference was performed by using Markov chain Monte Carlo (MCMC). Specifically, Hamiltonian Monte Carlo sampling was used as was implemented in the Stan probabilistic programming language (35). All statistical models were saved as .stan models and can be accessed at the GitHub repository associated with this work (DOI: 10.5281/zenodo.3366376) or can be downloaded directly from the paper website (https://www.rpgroup.caltech.edu/mwc_mutants/) (17).

### Inference of Free Energy From Fold-Change Data.

A more detailed summary and thorough analysis of the free-energy inference can be found in *SI Appendix*, *SI Text*. While the fold change in gene expression was restricted to be between 0 and 1, experimental noise can generate fold-change measurements beyond these bounds. To determine the free energy for a given set of fold-change measurements (for 1 unique strain at a single inducer concentration), we modeled the observed fold-change measurements as being drawn from a Gaussian distribution with a mean μ and SD σ and sampled the posterior distribution of these parameters using MCMC. For each MCMC sample of μ, the free energy was calculated by rearranging Eq. **3**. Using simulated data, we determined that when μ<σ or (1−μ)<σ, the mean fold change in gene expression was overestimated or underestimated for the lower and upper limit, respectively. This resulted in a systematic error in the calculation of the free energy, making proper inference beyond these limits difficult. This bounds the range in which we can confidently infer this quantity with flow cytometry. We further discuss details of this limitation in *SI Appendix*, *SI Text*.

### Data and Code Availability.

All data were collected, stored, and preserved by using the Git version control software. Code for data processing, analysis, and figure generation is available on the GitHub repository (https://github.com/rpgroup-pboc/mwc_mutants; DOI:10.5281/zenodo.3366376) or can be accessed via the paper website (https://www.rpgroup.caltech.edu/mwc_mutants/) (17). Raw flow cytometry data are stored on the CaltechDATA data repository and can be accessed via DOI 10.22002/D1.1241.

## Supplementary Material

Supplementary File
