# Supplementary material for: Predictive shifts in free energy couple mutations to their phenotypic consequences
Source: Proc Natl Acad Sci U S A. 2019 Aug 26;116(37):18275–84. doi: 10.1073/pnas.1907869116 (PMC6744869; doi:10.1073/pnas.1907869116)
Supplement: Supplementary File [file pnas.1907869116.sapp.pdf]

PNAS
www.pnas.org

Supplementary Information for

Predictive Shifts in Free Energy Couple Mutations to Their Phenotypic Consequences

Griffin Chure, Manuel Razo-Mejia, Nathan M. Belliveau, Tal Einav, Zofii A. Kaczmarek, Stephanie L. Barnes, Mitchell Lewis, Rob Phillips

Rob Phillips
E-mail: phillips@pboc.caltech.edu

This PDF file includes:

Supplementary text
Figs. S1 to S24
Tables S1 to S8
References for SI reference citations

Contents

1 Derivation of the Simple Repression Input-Output Function 3

2 Non-Monotonic Behavior of ΔF Under Changing K_A and K_I 5

3 Bayesian Parameter Estimation For DNA Binding Mutants 7

4 Inferring the Free Energy From Fold-Change Measurements 14

5 Additional Characterization of DNA Binding Mutants 19

6 Bayesian Parameter Estimation for Inducer Binding Domain Mutants 23

7 Additional Characterization of Inducer Binding Domain Mutants 29

8 Comparing Parameter Values To The Literature 34

9 Parameter Estimation Using All Induction Profiles 38

10 Generalizability of Data Collapse To Other Regulatory Architectures 43

Supporting Information Text

1. Derivation of the Simple Repression Input-Output Function

In this section, we derive the input-output function for the inducible simple repression motif. This section summarizes the results from Garcia and Phillips 2011 (1) and Razo-Mejia et al. 2018 (2) and we direct the reader to these references for further detail.

We begin by defining the simple repression motif as a regulatory architecture in which binding of a repressor to its cognate binding site occludes binding of an RNA polymerase (RNAP) to the promoter, thereby hindering gene expression (3, 4). The repressor in this work is considered to be an allosteric molecule which fluctuates between an active and inactive state in thermal equilibrium. Binding of an allosteric effector molecule (i.e. an inducer) to a binding site in one domain of the repressor can stabilize the inactive state relative to the active state. The repressor can still bind the DNA in the inactive state but the sequence specificity is reduced and binding to the cognate sequence becomes comparable to nonspecific binding. Such regulatory motifs have been well characterized from a thermodynamic perspective in which the system is considered to be in equilibrium at the time scales relevant to molecular binding events. Under such a model, we can assume that the level of gene expression is proportional to the the probability of RNAP being bound to the promoter and this has frequently been applied in thermodynamic models of transcription (1–10).

The probability of the promoter being occupied by either a polymerase, repressor, or neither is dictated by the Boltzmann distribution,

$$P_\text{state} \propto e^{-\varepsilon_\text{state}/k_B T}, \tag{1}$$

where ε_state is the energy of the state of interest. This energy is scaled to the thermal energy of the system $k_B T$ where k_B is Boltzmann's constant and T is the temperature in units of K. The goal of this section is to translate generic proportionality in Eq. (1) into the relevant states of our system.

The occupancy states of the promoter and corresponding statistical weights can be seen in Fig. S1 (A). Here we use P to denote the number of RNAP per cell, R_A as the number of active repressors, and R_I as the number of inactive repressors. We assume there is a single specific binding site on the genome and N_{NS} nonspecific binding sites. The polymerase, active, and inactive repressor in this work are considered to bind the DNA with different strengths. The energy of binding for each species is given as $\Delta\varepsilon_P$, $\Delta\varepsilon_{RA}$, or $\Delta\varepsilon_{RI}$, which captures the energetic difference between nonspecific and specific binding for that species. Since we consider a single specific binding site for both the polymerase and repressor, we can say that $N_{NS} >> P$ and $N_{NS} >> R_A + R_I$, allowing the multiplicity of arranging P polymerases and R repressors to be approximately equal to P/N_{NS} and $(R_A + R_I)/N_{NS}$, respectively.

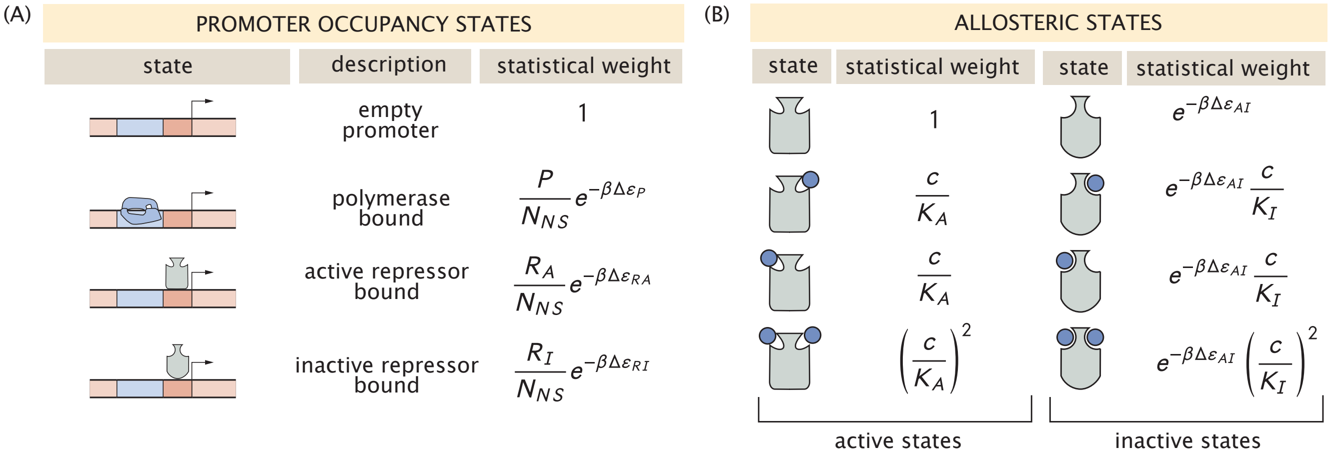

Fig. S1. States and statistical weights for a simple repression motif with an allosteric repressor. (A) Occupancy states of the promoter in which the statistical weights are relative to the unoccupied state. P, R_A, and R_I correspond to the average number of RNAP, active repressors, and inactive repressors per cell, respectively. The relative DNA binding energies are given as $\Delta\varepsilon_P$, $\Delta\varepsilon_{RA}$ and $\Delta\varepsilon_{RI}$. (B) Allosteric states of the repressor and the statistical weights relative to the active repressor with no inducer bound. The dissociation constant for the inducer to the active and inactive state of the repressor are denoted as K_A and K_I, respectively, c is the inducer concentration, and $\Delta\varepsilon_{AI}$ is the energetic difference between the active and inactive states of the repressor.

With these states and statistical weights enumerated, we can now define the probability of a polymerase being bound to the promoter as

$$P_\text{bound} = \frac{\frac{P}{N_{NS}} e^{-\beta\Delta\varepsilon_P}}{1 + \frac{P}{N_{NS}} e^{-\beta\Delta\varepsilon_P} + \frac{R_A}{N_{NS}} e^{-\beta\Delta\varepsilon_{RA}} + \frac{R_I}{N_{NS}} e^{-\beta\Delta\varepsilon_{RI}}}, \tag{2}$$

where we have defined β as $\frac{1}{k_B T}$.

It is experimentally difficult to measure P_bound directly as identifying the direct proportionality to gene expression is not straightforward. However, we can easily measure the fold-change in gene expression, defined as the probability of a polymerase

being bound to the promoter in the presence of repressor relative to constitutive expression,

$$\text{fold-change} = \frac{P_{\text{bound}}(R > 0)}{P_{\text{bound}}(R = 0)} = \frac{1 + \frac{P}{N_{NS}} e^{-\beta\Delta\varepsilon_P}}{1 + \frac{P}{N_{NS}} e^{-\beta\Delta\varepsilon_P} + \frac{R_A}{N_{NS}} e^{-\beta\Delta\varepsilon_{RA}} + \frac{R_I}{N_{NS}} e^{-\beta\Delta\varepsilon_{RI}}}. \quad [3]$$

This can be simplified by making two well justified approximations. We can assume that binding of the inactive repressor to the specific binding site is approximately equal to nonspecific binding, $\frac{R_I}{N_{NS}} e^{-\beta\Delta\varepsilon_{RI}} << 1 + \frac{R_A}{N_{NS}} e^{-\beta\Delta\varepsilon_{RA}}$. Secondly, we can state that binding of RNAP to the promoter is weak, $\frac{P}{N_{NS}} e^{-\beta\Delta\varepsilon_P} << 1$. Assuming $P \approx 10^3$ (11), $N_{NS} \approx 4.6 \times 10^6$ (the length of the *E. coli* genome in base pairs) and $\Delta\varepsilon_P \approx -2$ to $-5\,k_BT$ (12), the probability of this state comes to $\approx 1\%$ and can be neglected. Using these approximations, we can state that the fold-change in gene expression has the form

$$\text{fold-change} = \left(1 + \frac{R_A}{N_{NS}} e^{-\beta\Delta\varepsilon_{RA}}\right)^{-1}. \quad [4]$$

In order to make falsifiable predictions, we must have a precise knowledge of the number of active repressors in the cell R_A. While determining this quantity is fraught with experimental difficulties, it is relatively easy to determine the total number of repressors per cell R through quantitative Western blotting (1), fluorescence based methods (9), or proteomic studies (13). We can compute the number of active repressors at a given inducer concentration c by multiplying the total number of repressors by the probability of a repressor being active at that inducer concentration,

$$R_A(c) = p_{\text{act}}(c) R. \quad [5]$$

Similarly to computing P_{bound}, we can compute the possible states and statistical weights of the repressor activity, shown in Fig. S1 (B). Following the model of Monod, Wyman, and Changeux (14), we have defined all statistical weights relative to the active repressor with no bound inducer molecules. We have defined the dissociation constant of the inducer to the active and inactive repressor as K_A and K_I, respectively, and have assigned an energetic penalty $e^{-\beta\Delta\varepsilon_{AI}}$ to all inactive states of the repressor. The energetic term $\Delta\varepsilon_{AI}$ represents the relative energy difference between the active and inactive states, $\Delta\varepsilon_{AI} = \varepsilon_I - \varepsilon_A$. For the *lac* repressor used in this work, we have inferred the value of $\Delta\varepsilon_{AI}$ to be $4.5\,k_BT$, indicating that the active state is energetically preferred and with no inducer, approximately 99% of the repressors are in the active state. In Sec. 10 of this supplement, we compare this inferred value (along with those of K_A, K_I, and $\Delta\varepsilon_{RA}$) with previously reported values determined from *in vivo* and *in vitro* experiments.

Using these states and weights, we can compute $P_{\text{act}}(c)$ as

$$p_{\text{act}}(c) = \frac{\left(1 + \frac{c}{K_A}\right)^2}{\left(1 + \frac{c}{K_A}\right)^2 + e^{-\beta\Delta\varepsilon_{AI}} \left(1 + \frac{c}{K_I}\right)^2}. \quad [6]$$

Using Eq. (4) - Eq. (6), we can then state that at a given inducer concentration c, the fold-change in gene expression can be defined as

$$\text{fold-change} = \left(1 + \frac{\left(1 + \frac{c}{K_A}\right)^2}{\left(1 + \frac{c}{K_A}\right)^2 + e^{-\beta\Delta\varepsilon_{AI}} \left(1 + \frac{c}{K_I}\right)^2} \frac{R}{N_{NS}} e^{-\beta\Delta\varepsilon_{RA}}\right)^{-1}, \quad [7]$$

which is the result stated in Eq. 1 of the main text. We emphasize that equilibrium models as derived here have frequently been used to characterize the simple repression motif (1–5, 9) in addition to non-equilibrium approaches which have the same functional form as Eq. (7) (15).

2. Non-Monotonic Behavior of ΔF Under Changing K_A and K_I

In the main text, we illustrated that perturbations only to the allosteric parameters K_A and K_I relative to the wild-type values can result in a non-monotonic dependence of ΔF on the inducer concentration c. In this section, we prove that when the ratio of K_A to K_I is the same between the mutant and wild-type proteins, the function must be monotonic. This section is paired with an interactive figure available on the paper website which illustrates how scaling K_A and K_I relative to the wild-type value results in non-monotonic behavior.

We define a monotonic function as a continuous function whose derivative does not change sign across the domain upon which it is defined. To show that ΔF is non-monotonic when K_A and K_I are perturbed, we can compute the derivative of ΔF with respect to the inducer concentration c and evaluate the sign of the derivative at the limits of inducer concentration. If the sign of the derivative is different at the limits of $c = 0$ and $c \gg 0$, we can see that the function is non-monotonic. However, if the sign is the same in both limits, we can not say conclusively if it is non-monotonic and must consider other diagnostics.

The free energy difference between a mutant and wild-type repressor when all parameters other than K_A and K_I are unperturbed can be written as

$$\beta \Delta F(c) = -\log\left(\frac{\left[1 + e^{-\beta\Delta\varepsilon_{AI}}\left(\frac{1+\frac{c}{K_I^{(\text{mut})}}}{1+\frac{c}{K_A^{(\text{mut})}}}\right)^2\right]^{-1}}{\left[1 + e^{-\beta\Delta\varepsilon_{AI}}\left(\frac{1+\frac{c}{K_I^{(\text{wt})}}}{1+\frac{c}{K_A^{(\text{wt})}}}\right)^2\right]^{-1}}\right), \quad [8]$$

in which $\Delta\varepsilon_{AI}$ is the energy difference between the active and inactive states of the repressor, c is the inducer concentration, and $\beta = 1/k_BT$ where k_B is the Boltzmann constant and T is the temperature. The derivative with respect to c, which we determined using Mathematica's (Wolfram Research, version 11.2) symbolic computing ability, is given as

$$\frac{\partial \beta\Delta F(c)}{\partial c} = 2e^{-\beta\Delta\varepsilon_{AI}}\left(\frac{K_A^{(\text{mut})2}\left(K_A^{(\text{mut})} - K_I^{(\text{mut})}\right)\left(c + K_I^{(\text{mut})}\right)}{\left(c + K_A^{(\text{mut})}\right)\left[\left(c + K_A^{(\text{mut})}\right)^2 K_I^{(\text{mut})2} + e^{-\beta\Delta\varepsilon_{AI}} K_A^{(\text{mut})2}\left(c + K_I^{(\text{mut})}\right)^2\right]} - \frac{K_A^{(\text{wt})2}\left(K_A^{(\text{wt})} - K_I^{(\text{wt})}\right)\left(c + K_I^{(\text{wt})}\right)}{\left(c + K_A^{(\text{wt})}\right)\left[\left(c + K_A^{(\text{wt})}\right)^2 K_I^{(\text{wt})2} + e^{-\beta\Delta\varepsilon_{AI}} K_A^{(\text{wt})2}\left(c + K_I^{(\text{wt})}\right)^2\right]}\right). \quad [9]$$

This unwieldy expression can be simplified by defining the values of $K_A^{(\text{mut})} = \theta K_A^{(\text{wt})}$ and $K_I^{(\text{mut})} = \theta K_I^{(\text{wt})}$ as relative changes to the wild-type values where θ is a scaling parameter. While we can permit $K_A^{(\text{mut})}$ and $K_I^{(\text{mut})}$ to vary by different degrees, we will consider the case in which they are equally perturbed such that the ratio of K_A to K_I is the same between the mutant and wild-type versions of the repressor. While the equations become more cumbersome when one permits the dissociation constants to vary by different amounts (i.e. $\theta_{K_A}, \theta_{K_I}$), one arrives at the same conclusion. This definition allows us to rewrite Eq. 9 in the form of

$$\frac{\partial \beta\Delta F(c)}{\partial c} = 2K_A^{(\text{wt})} e^{-\beta\Delta\varepsilon_{AI}}\left(\frac{\theta^3\left(K_A^{(\text{wt})} - K_I^{(\text{wt})}\right)\left(c + \theta K_I^{(\text{wt})}\right)}{\left(c + \theta K_A^{(\text{wt})}\right)\left[\theta^2 K_I^{(\text{wt})2}\left(c + \theta K_A^{(\text{wt})}\right)^2 + e^{-\beta\Delta\varepsilon_{AI}}\theta^2 K_A^{(\text{wt})2}\left(c + \theta K_I^{(\text{wt})}\right)^2\right]} - \frac{\left(K_A^{(\text{wt})} - K_I^{(\text{wt})}\right)\left(c + K_I^{(\text{wt})}\right)}{\left(c + K_A^{(\text{wt})}\right)\left[\left(c + K_A^{(\text{wt})}\right)^2 K_I^{(\text{wt})2} + e^{-\beta\Delta\varepsilon_{AI}} K_A^{(\text{wt})2}\left(c + K_I^{(\text{wt})}\right)^2\right]}\right). \quad [10]$$

With this derivative in hand, we can examine the limits of inducer concentration. As discussed in the main text, the free energy difference between the mutant and wild-type repressors when $c = 0$ should be equal to 0. However, the derivative at $c = 0$ will be different between the wild-type and the mutant. In this limit, Eq. 10 simplifies to

$$\left.\frac{\partial \beta\Delta F(c)}{\partial c}\right|_{c=0} = \frac{2e^{-\beta\Delta\varepsilon_{AI}}\left(K_A^{(\text{wt})} - K_I^{(\text{wt})}\right)}{K_A^{(\text{wt})} K_I^{(\text{wt})}\left(1 + e^{-\beta\Delta\varepsilon_{AI}}\right)}\left(\frac{1}{\theta} - 1\right). \quad [11]$$

When $\theta < 1$, meaning that the affinity of the acitve and inactive states of the repressor to the inducer is increased relative to wild-type, the derivative is positive. Thus, the repressor bound state of the promoter becomes less energetically favorable than the repressor bound state. Similarly, if $\theta > 1$, binding of the inducer to the mutant repressor is weaker than the wild-type repressor, making $\partial\beta\Delta F(c)/\partial c < 0$, meaning the repressor bound state becomes more energetically favorable than the repressor unbound state of the promoter.

With an intuition for the sign of the derivative when $c = 0$, we can compute the derivative at another extreme where $c \gg 0$. Here, Eq. 10 reduces to

$$\left.\frac{\partial \beta\Delta F(c)}{\partial c}\right|_{c\gg 0} \approx \frac{2e^{-\beta\Delta\varepsilon_{AI}} K_A^{(\text{wt})2}\left(K_A^{(\text{wt})} - K_I^{(\text{wt})}\right)}{c^2\left(K_I^{(\text{wt})2} + e^{-\beta\Delta\varepsilon_{AI}}K_A^{(\text{wt})2}\right)}(\theta - 1)\,. \qquad [12]$$

when $\theta > 1$, Eq. 12 is positive. This is the opposite sign of the derivative when $c = 0$ when $\theta > 1$. When $\theta < 1$, Eq. 12 becomes negative whereas Eq. 11 is positive. As the derivative of ΔF with respect to c changes signs across the defined range of inducer concentrations, we can say the function is non-monotonic.

Fig. S2 shows the non-monotonic behavior of ΔF when K_A and K_I change by the same factor θ [maintaining the wild-type ratio, Fig. S2(A)] and when K_A and K_I change by different factors [Fig. S2(B)]. In both cases, non-monotonic behavior is observed with the peak difference in the free energy covering several k_BT. We have hosted an interactive figure similar to Fig. S2 on the paper website where the reader can modify how K_A and K_I are affected by a mutation and examine how the active probability, free energy difference, and $\partial\beta\Delta F/\partial c$ are tuned.

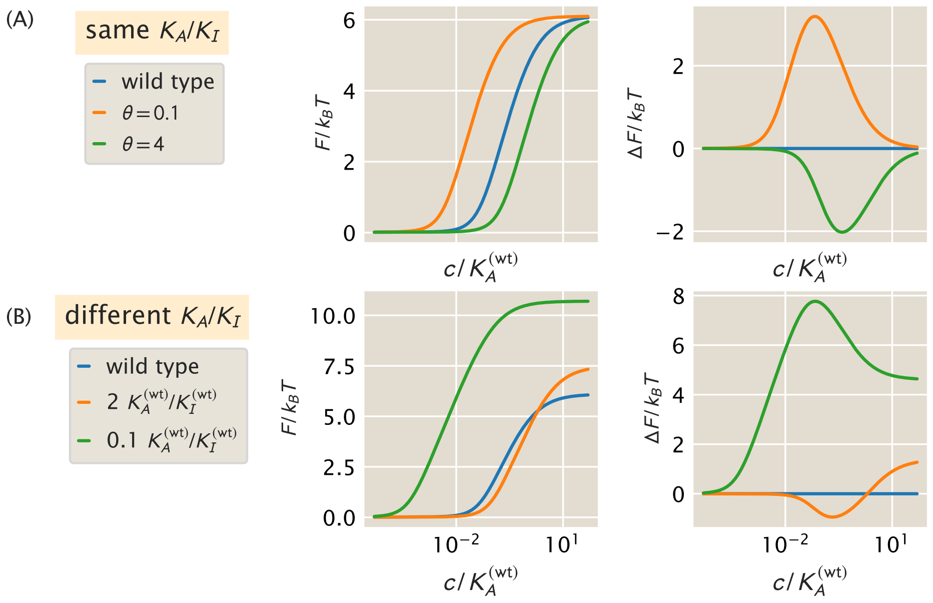

Fig. S2. Non-monotonic behavior of ΔF with change in K_A and K_I. Middle column shows the allosteric contribution of free energy F plotted as a function of the inducer concentration. Right column shows the free energy difference ΔF as a function of inducer concentration, revealing non-monotonicity (A) Behavior of F and ΔF when the values of K_A and K_I change relative to wild-type, but maintain the same ratio. θ is the scaling factor for both inducer dissociation constants. (B) Behavior of F and ΔF when the values of K_A and K_I change relative to the wild-type, but by different factors. In both panels, the wild-type parameter values were taken to be $K_A = 200\,\mu$M, $K_I = 1\,\mu$M, and $\Delta\varepsilon_{AI} = 4.5\,k_BT$. An interactive version of this figure is available on the paper website.

3. Bayesian Parameter Estimation For DNA Binding Mutants

In this section, we outline the statistical model used in this work to estimate the DNA binding energy for a given mutation in the DNA binding domain. We begin with a derivation of our statistical model using Bayes' theorem and then perform a series of principled steps to validate our choices of priors, ensure computational feasibility, and assess the validity of the model given the collected data. This work follows the analysis pipeline outlined by Michael Betancourt in his case-study entitled "Towards A Principled Bayesian Workflow."

The second subsection *"Building a Generative Statistical Model"* lays out the statistical model used in this work to estimate the DNA binding energy and the error term σ. The subsequent subsections – *"Prior Predictive Checks"*, *"Simulation Based Calibration"*, and *"Posterior Predictive Checks"* – define and summarize a series of tests that ensure that the parameters of the statistical model can be identified and are computationally tractable. To understand how we defined our statistical model, only the second subsection is needed.

Calculation of the Fold-Change in Gene Expression. We appreciate the subtleties of the efficiency of photon detection in the flow cytometer, fluorophore maturation and folding, and autofluorescence correction, and we understand the importance in modeling the effects that these processes have on the reported value of the fold-change. However, in order to be consistent with the methods used in the literature, we took a more simplistic approach to calculate the fold-change. Given a set of fluorescence measurements of the constitutive expression control ($R = 0$), an autofluorescence control (no YFP), and the experimental strain ($R > 0$), we calculate the fold-change as

$$\text{fold-change} = \frac{\langle I_{\text{cell}}(R > 0)\rangle - \langle I_{\text{autofluorescence}}\rangle}{\langle I_{\text{cell}}(R = 0)\rangle - \langle I_{\text{autofluorescence}}\rangle}. \qquad [13]$$

It is important to note here that for a given biological replicate, we consider only a point estimate of the mean fluorescence for each sample and perform a simple subtraction to adjust for background fluorescence. For the analysis going forward, all mentions of measured fold-change are determined by this calculation.

Building a Generative Statistical Model. To identify the minimal parameter set affected by a mutation, we assume that mutations in the DNA binding domain of the repressor alters only the DNA binding energy $\Delta\varepsilon_{RA}$, while the other parameters of the repressor are left unperturbed from their wild-type values. As a first approach, we can assume that all of the other parameters are known without error and can be taken as constants in our physical model. Ultimately, we want to know how probable a particular value of $\Delta\varepsilon_{RA}$ is given a set of experimental measurements y. Bayes' theorem computes this distribution, termed the *posterior distribution* as

$$g(\Delta\varepsilon_{RA}\,|\,y) = \frac{f(y\,|\,\Delta\varepsilon_{RA})g(\Delta\varepsilon_{RA})}{f(y)}, \qquad [14]$$

where we have used g and f to represent probability densities over parameters and data, respectively. The expression $f(y\,|\Delta\varepsilon_{RA})$ captures the likelihood of observing our data set y given a value for the DNA binding energy under our physical model. All knowledge we have of what the DNA binding energy *could* be, while remaining completely ignorant of the experimental measurements, is defined in $g(\Delta\varepsilon_{RA})$, referred to as the *prior distribution*. Finally, the likelihood that we would observe the data set y while being ignorant of our physical model is defined by the denominator $f(y)$. In this work, this term serves only as a normalization factor and as a result will be treated as a constant. We can therefore say that the posterior distribution of $\Delta\varepsilon_{RA}$ is proportional to the joint distribution between the likelihood and the prior,

$$g(\Delta\varepsilon_{RA}\,|\,y) \propto f(y\,|\,\Delta\varepsilon_{RA})g(\Delta\varepsilon_{RA}). \qquad [15]$$

We are now tasked with translating this generic notation into a concrete functional form. Our physical model given by Eq. (7) computes the average fold-change in gene expression. Speaking practically, we make several replicate measurements of the fold-change to reduce the effects of random errors. As each replicate is independent of the others, it is reasonable to expect that these measurements will be normally distributed about the theoretical value of the fold-change μ, computed for a given $\Delta\varepsilon_{RA}$. We can write this mathematically for each measurement as

$$f(y\,|\,\Delta\varepsilon_{RA}) = \frac{1}{(2\pi\sigma^2)^{N/2}}\prod_i^N \exp\left[\frac{-(y_i - \mu(\Delta\varepsilon_{RA}))^2}{2\sigma^2}\right], \qquad [16]$$

where N is the number of measurements in y and y_i is the ith experimental fold-change measurement. We can write this likelihood in shorthand as

$$f(y\,|\,\Delta\varepsilon_{RA}) = \text{Normal}\{\mu(\Delta\varepsilon_{RA}), \sigma\} \qquad [17]$$

which we will use for the remainder of this section.

Using a normal distribution for our likelihood has introduced a new parameter σ which describes the spread of our measurements about the true value. We must therefore include it in our parameter estimation and assign an appropriate prior distribution such that the posterior distribution becomes

$$g(\Delta\varepsilon_{RA}, \sigma\,|y) \propto f(y\,|\,\Delta\varepsilon_{RA}, \sigma)g(\Delta\varepsilon_{RA})g(\sigma). \qquad [18]$$

We are now tasked with assigning functional forms to the priors $g(\Delta\varepsilon_{RA})$ and $g(\sigma)$. Though one hopes that the result of the inference is not too dependent on the choice of prior, it is important to choose one that is in agreement with our physical and physiological intuition of the system.

We can impose physically reasonable bounds on the possible values of the DNA binding energy $\Delta\varepsilon_{RA}$. We can say that it is unlikely that any given mutation in the DNA binding domain will result in an affinity greater than that of biotin to streptavidin [1 fM $\approx -35\,k_BT$, BNID 107139 (16)], one of the strongest known non-covalent bonds. Similarly, it's unlikely that a given mutation will result in a large, positive binding energy, indicating non-specific binding is preferable to specific binding ($\sim$ 1 to 10 k_BT). While it is unlikely for the DNA binding energy to exceed these bounds, it's not impossible, meaning we should not impose these limits as hard boundaries. Rather, we can define a weakly informative prior as a normal distribution with a mean and standard deviation as the average of these bounds,

$$g(\Delta\varepsilon_{RA}) \sim \text{Normal}\{-12, 12\} \quad [19]$$

whose probability density function in shown in Fig. S3 (A).

By definition, fold-change is restricted to the bounds $[0, 1]$. Measurement noise and fluctuations in autofluorescence background subtraction means that experimental measurements of fold-change can extend beyond these bounds, though not substantially. By definition, the scale parameter σ must be positive and greater than zero. We also know that for the measurements to be of any use, the error should be less than the available range of fold-change, 1.0. We can choose such a prior as a half normal distribution

$$g(\sigma) = \frac{1}{\phi}\sqrt{\frac{2}{\pi}} \exp\left[-\frac{\sigma^2}{2\phi^2}\right]; \forall\, \sigma \geq 0, \quad [20]$$

where ϕ is the standard deviation. By choosing $\phi = 0.1$, it is unlikely that $\sigma \geq 1$ yet not impossible, permitting the occasional measurement significantly outside of the theoretical bounds. The probability density function for this prior is shown in Fig. S3(B).

While these choices for the priors seem reasonable, we can check their appropriateness by using them to simulate a data set and checking that the hypothetical fold-change measurements obey our physical and physiological intuition.

Prior Predictive Checks. If our choice of prior distribution for each parameter is appropriate, we should be able to simulate data sets using these priors that match our expectations. In essence, we would hope that these prior choices would generate some data sets with fold-change measurements above 1 or below zero, but they should be infrequent. If we end up getting primarily negative values for fold-change, for example, then we can surmise that there is something wrong in our definition of the prior distribution. This method, coined a *prior predictive check*, was first put forward by Isidore Good in 1950 (17) and has received newfound attention in computational statistics.

We perform the simulation in the following manner. We first draw a random value for $\Delta\varepsilon_{RA}$ out of its prior distribution stated in Eq. (19) and calculate what the mean fold-change should be given our theory described in Eq. (7). With this in hand, we draw a random value for σ from its prior distribution, specified in Eq. (20). We then generate a simulated dataset by drawing ≈ 70 fold-change values across twelve inducer concentrations from the likelihood distribution which we defined in Eq. (17). This roughly matches the number of measurements made for each mutant in this work. We repeat this procedure for 800 draws from the prior distributions, which is enough to observe the occasional extreme fold-change value from the likelihood. As the DNA binding energy is the only parameter of our physical model that we are estimating, we had to choose values for the others. We kept the values of the inducer binding constants K_A and K_I the same as the wild-type repressor (139 μM and 0.53 μM, respectively). We chose to use $R = 260$ repressors per cell as this is the repressor copy number we used in the main text to estimate the DNA binding energies of the three mutants.

The draws from the priors are shown in S3(A) and (B) as black points above the corresponding distribution. To display the results, we computed the percentiles of the simulated data sets at each inducer concentration. These percentiles are shown as red shaded regions in Fig. S3(C). The 5th percentile (dark red band) has the characteristic profile of an induction curve. Given that the prior distribution for $\Delta\varepsilon_{RA}$ is centered at $-12\,k_BT$ and we chose $R = 260$, we expect the generated data sets to cluster about the induction profile defined by these values. More importantly, approximately 95% of the generated data sets fall between fold-change values of -0.1 and 1.1, which is within the realm of possibility given the systematic and biological noise in our experiments. The 99th percentile maximum is approximately 1.3 and the minimum approximately -0.3. While we could tune our choice of prior further to minimize draws this far from the theoretical bounds, we err on the side of caution and accept these values as it is possible that fold-change measurements this high or low can be observed, albeit rarely.

Through these prior predictive checks, we feel confident that these choices of priors are appropriate for the parameters we wish to estimate. We can now move forward and make sure that the statistical model as a whole is valid and computationally tractable.

Sensitivity Analysis and Simulation Based Calibration. Satisfied with our choice of prior distributions, we can proceed to check other properties of the statistical model and root out any pathologies lurking in our model assumptions.

To build trust in our model, we could generate a data set $\tilde{y}$ with a *known* value for σ and $\Delta\varepsilon_{RA}$, estimate the posterior distribution $g(\Delta\varepsilon_{RA}, \sigma \mid \tilde{y})$, and determine how well we were able to retrieve the true value of the parameters. However, running this once or twice for handpicked values of σ and $\Delta\varepsilon_{RA}$ won't reveal edge-cases in which the inference fails, some of which may exist in our data. Rather than performing this operation once, we can run this process over a variety of data sets where the ground truth parameter value is drawn from the prior distribution (as we did for the prior predictive checks). For an arbitrary parameter θ, the joint distribution between the ground truth value $\tilde{\theta}$, the inferred value θ, and the simulated data set $\tilde{y}$ can be written as

$$\pi(\theta, \tilde{y}, \tilde{\theta}) = g(\theta \mid \tilde{y}) f(\tilde{y} \mid \tilde{\theta}) g(\tilde{\theta}). \quad [21]$$

 Griffin Chure, Manuel Razo-Mejia, Nathan M. Belliveau, Tal Einav, Zofii A. Kaczmarek, Stephanie L. Barnes, Mitchell Lewis, Rob Phillips

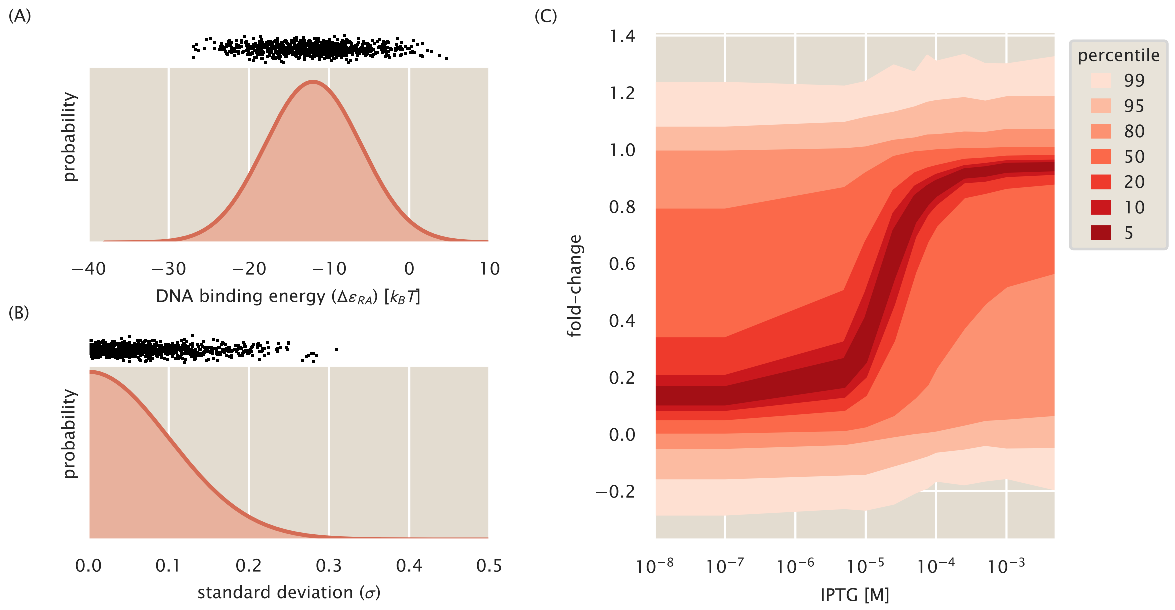

Fig. S3. Prior distributions and prior predictive check for estimation of the DNA binding energy. (A) Prior probability density function for DNA binding energy $\Delta\varepsilon_{RA}$ as $\sim$ Normal$(-12, 12)$. (B) Prior probability density function for the standard deviation in measurement noise σ as $\sim$ HalfNormal$(0, 0.1)$. (C) Percentiles of values drawn from the likelihood distribution given draws from prior distributions given $R = 260$, $K_A = 139 \times 10^{-6}$ M, $K_I = 0.53 \times 10^{-6}$ M, and $\Delta\varepsilon_{AI} = 4.5\, k_BT$, which match the parameters used for the predictions in Razo-Mejia et al. 2018 (2). Black points at top of (A) and (B) represent draws used to generate fold-change measurements from the likelihood distribution. Percentiles in (C) generated from 800 draws from the prior distributions. For each draw from the prior distributions, a data set of 70 measurements over 12 IPTG concentrations (ranging from 0 to 5000 μM) were generated from the likelihood.

If this process is run for a large number of simulations, Eq. (21) can be marginalized over all data sets $\tilde{y}$ and all ground truth values $\tilde{\theta}$ to yield the original prior distribution,

$$\int d\tilde{\theta} \int d\tilde{y} \pi(\theta, \tilde{y}, \tilde{\theta}) = g(\theta). \tag{22}$$

This result, described by Talts et al. 2018 (18), holds true for *any* statistical model and is a natural self consistency property of Bayesian inference. Any deviation between the distribution of our inferred values for θ and the original prior distribution $g(\theta)$ indicates that either our statistical model is malformed or the computational method is not behaving as expected. There are a variety of ways we can ensure that this condition is satisfied, which we outline below.

Using the data set generated for the prior predictive checks [shown in Fig. S3(C)], we sampled the posterior distribution and compute $\Delta\varepsilon_{RA}$ and σ for each simulation and checked that they matched the original prior distribution. To perform the inference, we use Markov chain Monte Carlo (MCMC) to sample the posterior distribution. Specifically, we use the Hamiltonian Monte Carlo algorithm implemented in the Stan probabilistic programming language (19). The specific code files can be accessed through the paper website or the associated GitHub repository. The original prior distribution and the distribution of inferred parameter values can be seen in Fig. S4 (A) and (B). For both $\Delta\varepsilon_{RA}$ and σ, we can accurately recover the ground truth distribution (blue) via sampling with MCMC (red). For $\Delta\varepsilon_{RA}$, there appears to be an upper and lower limit past which we are unable to accurately infer the binding energy. This can be seen in both the histogram [Fig. S4(A)] and the empirical cumulative distribution [Fig. S4(B)] as deviations from the ground truth when DNA binding is below $\approx -25k_BT$ or above $\approx -5k_BT$. These limits hinder our ability to comment on exceptionally strong or weak binding affinities. However, as all mutants queried in this work exhibited binding energies between these limits, we surmise that the inferential scheme permits us to draw conclusions about the inferred DNA binding strengths.

Rather than examining the agreement of the data-averaged posterior and the ground truth prior distribution solely by eye, we can compute summary statistics using the mean μ and standard deviation σ of the posterior and prior distributions which permit easier identification of pathologies in the inference. One such quantity is the posterior z-score, which is defined as

$$z = \frac{\mu_{\text{posterior}} - \tilde{\theta}}{\sigma_{\text{posterior}}}. \tag{23}$$

This statistic summarizes how accurately the posterior recovers the ground truth value beyond simply reporting the mean, median, or mode of the posterior distribution. Z-scores around 0 indicate that the posterior is concentrating tightly about the true value of the parameter whereas large values (either positive or negative) indicate that the posterior is concentrating elsewhere. A useful feature of this metric is that the width of the posterior is also considered. It is possible that the posterior could have a mean very close to the ground truth value, but have an incredibly narrow distribution/spread such that it does not overlap with the

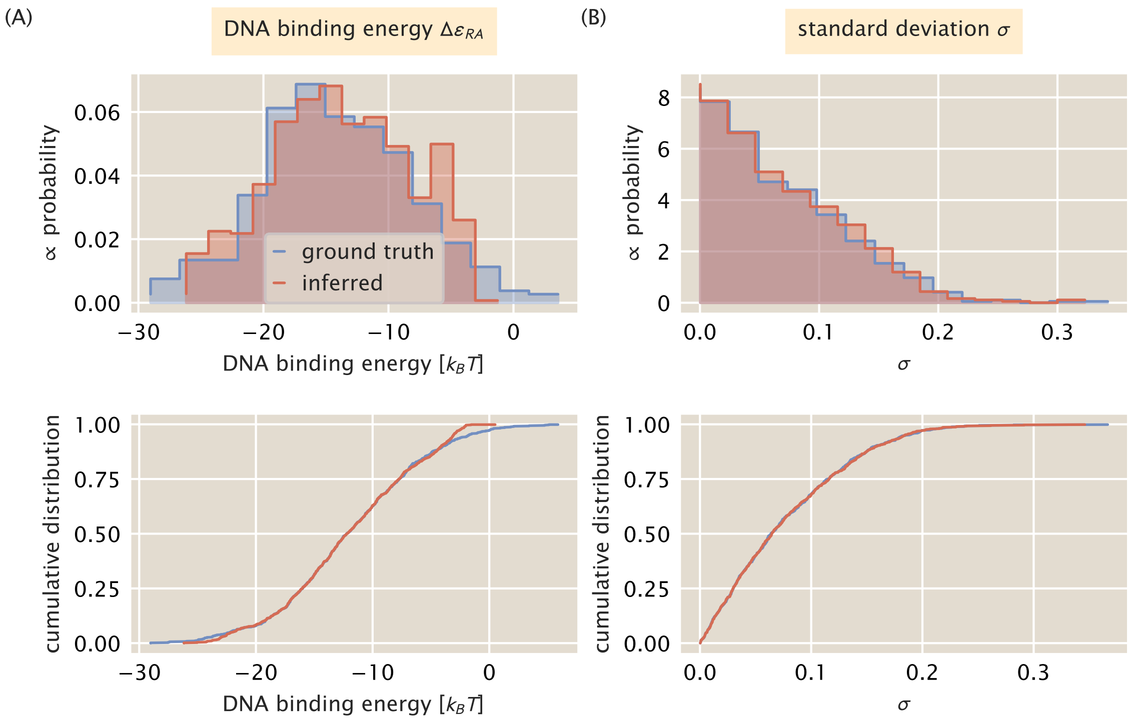

Fig. S4. Comparison of averaged posterior and prior distributions for $\Delta\varepsilon_{RA}$ and σ. (A) Distribution of the average values for the DNA binding energy $\Delta\varepsilon_{RA}$ (red) overlaid with the ground truth distribution (blue). (B) Data averaged posterior (red) for the standard deviation of fold-change measurements overlaid with the ground truth distribution (blue). Top and bottom show the same data with different visualizations.

ground-truth. Only comparing the mean value to the ground truth would suggest that the inference "worked". However with a small standard deviation generates a very large z-score, telling us that something has gone awry.

If our inferential model is behaving properly, the width of the posterior distribution should be significantly smaller than the width of the prior, meaning that the posterior is being informed by the data. The level to which the posterior is being informed by the data can be easily calculated given knowledge of both the prior and posterior distribution. This quantity, aptly named the shrinkage s, can be computed as

$$s = 1 - \frac{\sigma^2_{\text{posterior}}}{\sigma^2_{\text{prior}}}. \quad [24]$$

When the shrinkage is close to zero, the variance of the posterior is approximately the same as the variance of the prior, model is not being properly informed by the data. When $s \approx 1$, the variance of the posterior is much smaller than the variance of the prior, indicating that the it is being highly informed by the data. A shrinkage less than 0 indicates that the posterior is wider than the prior distribution, revealing a severe pathology in either the model itself or the implementation.

In Fig. S5, we compute these summary statistics for each parameter. For both $\Delta\varepsilon_{RA}$ and σ, we see clustering of the z-score about 0 with the extrema reaching $\approx \pm 3$. This suggests that for the vast majority of our simulated data sets, the posterior distribution concentrated about the ground truth value. We also see that for both parameters, the posterior shrinkage s is ≈ 1, indicating that the posterior is being highly informed by the data. There is a second distribution centered ≈ 0.8 for $\Delta\varepsilon_{RA}$, indicating that for a subset of the data sets, the posterior is only $\approx 80\%$ narrower than the prior distribution. These samples are those that were drawn outside of the limits of ≈ -25 to $-5\, k_BT$ where the inferential power is limited. Nevertheless, the posterior still significantly shrank, indicating that the data strongly informs the posterior.

The general self-consistency condition given by Eq. (22) provides another route to ensure that the model is computationally tractable. Say that we draw a value for the DNA binding energy from the prior distribution, simulate a data set, and sample the posterior using MCMC. The result of this sampling is a collection of N values of the parameter which may be above, below, or equal to the ground-truth value. From this set of values, we select L of them and rank order them by their value. Talts and colleagues (18) derived a general theorem which states that the number of samples less than the ground truth value of the parameter (termed the rank statistic) is uniformly distributed over the interval $[0, L]$. As Eq. (22) *must* hold true for any statistical model, deviations from uniformity signal that there is a problem in the implementation of the statistical model. How the distribution deviates is also informative as different types of failures result in different distributions. The nature of these deviations, along with a more formal proof of the uniform distribution of rank statistics can be found in Talts et al. 2018 (18) where it was originally derived.

Given the sampling statistics for each of the simulated data sets, we took 800 of the MCMC samples of the posterior distribution

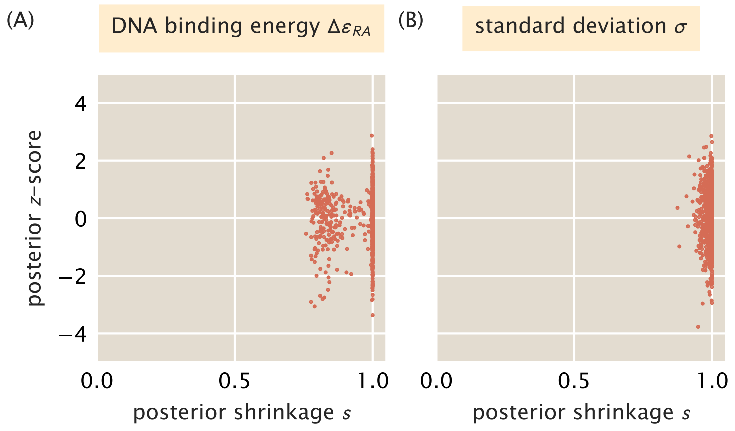

Fig. S5. Inferential sensitivity for estimation of $\Delta\varepsilon_{RA}$ and σ. The posterior z-score for each posterior distribution inferred from a simulated data set is plotted against the shrinkage for (A) the DNA binding energy $\Delta\varepsilon_{RA}$ and (B) the standard deviation of fold-change measurements σ

for each of the 800 simulated data sets and computed the rank statistic. The distributions are shown in Fig. S6 as both histograms and ECDFs for the DNA binding energy and standard deviation. The distribution of rank statistics for both parameters appears to be uniform. The gray band overlaying the histograms (top row) as well as the gray envelopes overlaying the ECDFs (bottom row) represent the 99th percentile expected from a true uniform distribution. The uniformity of this distribution, along with the well-behaved z-scores and shrinkage for each parameter, tells us that there are no underlying pathologies in our statistical model and that it is computationally tractable. However, this does not mean that it is correct. Whether this model is valid for the actual observed data is the topic of the next section.

Parameter Estimation and Posterior Predictive Checks. We now turn to applying our vetted statistical model to experimental measurements. While the same statistical model was applied to all three DNA binding mutants, here we only focus on the mutant Q18M for brevity.

Using a single induction profile, we sampled the posterior distribution over both the DNA binding energy $\Delta\varepsilon_{RA}$ and the standard deviation σ using MCMC implemented in the Stan programming language. The output of this process is a set of 4000 samples of both parameters along with the value of their log posterior probabilities, which serves as an approximate measure of the probability of each value. The individual samples are shown in Fig. S7. The joint distribution between $\Delta\varepsilon_{RA}$ and σ is shown in the lower left hand corner, and the marginal distributions for each parameter are shown above and to the right of the joint distribution, respectively. The joint distribution is color coded by the value of the log posterior, with yellow and blue corresponding to high and low probability, respectively. The symmetric shape of the joint distribution is a telling sign that there is no correlation between two parameters. The marginal distributions for each parameter are also relatively narrow, with the DNA binding energy covering a range of $\approx 0.6\, k_BT$ and σ spanning ≈ 0.02. To more precisely quantify the uncertainty, we computed the shortest interval of the marginal distribution for each parameter contains 95% of the probability. The bounds of this interval, coined the Bayesian credible region, can accommodate asymmetry in the marginal distribution since the upper and lower bounds of the estimate are reported. In the main text, we reported the DNA binding energy estimated from these data to be $15.43^{+0.06}_{-0.06}\, k_BT$, where the first value is the median of the distribution and the super- and subscripts correspond to the upper and lower bounds of the credible region, respectively.

While looking at the shape of the posterior distribution can be illuminating, it is not enough to tell us if the parameter values extracted make sense or accurately describe the data on which they were conditioned. To assess the validity of the statistical model in describing actual data, we again turn to simulation, this time using the posterior distributions for each parameter rather than the prior distributions. The likelihood of our statistical model assumes that across the entire induction profile, the observed fold-change is normally distributed about the theoretical prediction with a standard deviation σ. If this is an accurate depiction of the generative process, we should be able to draw values from the likelihood using the sampled values for $\Delta\varepsilon_{RA}$ and σ that are indistinguishable from the actual experimental measurements. This process is known as a *posterior predictive check* and is a Bayesian method of assessing goodness-of-fit.

For each sample from the posterior, we computed the theoretical mean fold-change given the sampled value for $\Delta\varepsilon_{RA}$ and Eq. (7). With this mean in hand, we used the corresponding sample for σ and drew a data set from the likelihood distribution the same size as the real data set used for the inference. As we did this for every sample of our MCMC output (a total of $\approx$ 4000), it is more instructive to compute the percentiles of the generated data than to show the entire output. In Fig. S7(B), the percentiles of the generated data sets are shown overlaid with the data used for the inference. We see that all of the data points fall within the 99th percentile of simulated data sets with the 5th percentile tracking the mean of the data at each inducer concentration. As there are no systematic deviations or experimental observations that fall far outside those generated from the statistical model, we can safely say that the statistical model derived here accurately describes the observed data.

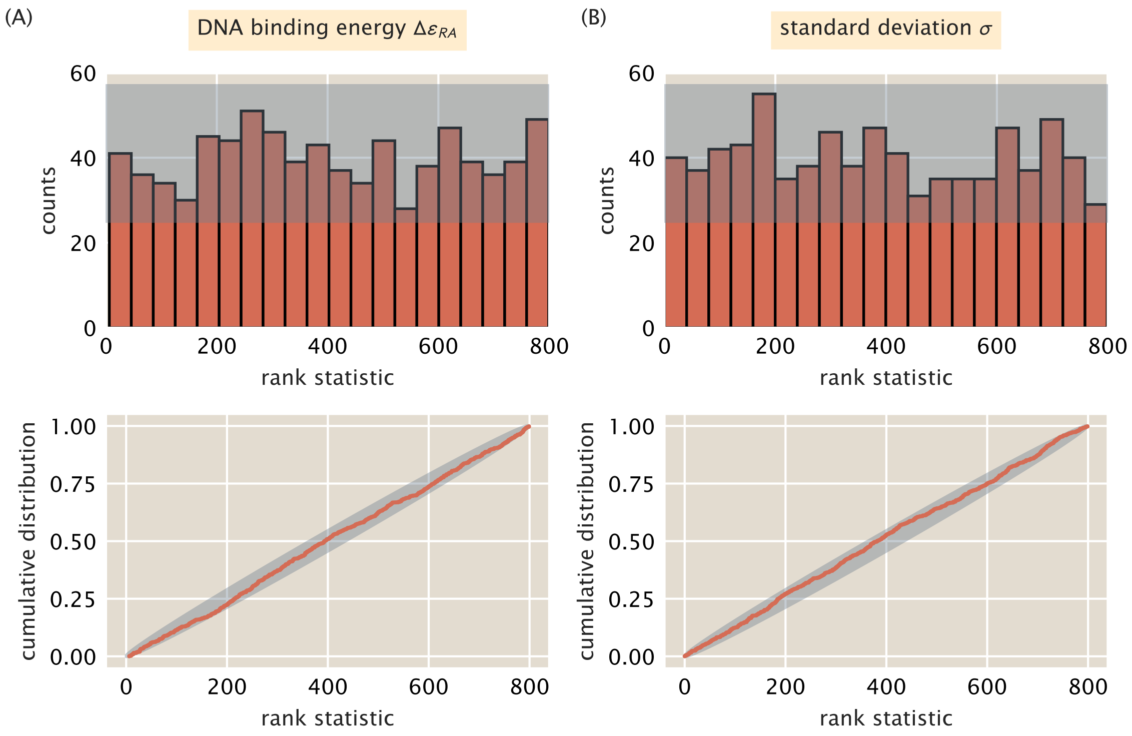

Fig. S6. Rank distribution of the posterior samples from simulated data. Top row shows a histogram of the rank distribution with $n = 20$ bins. Bottom row is the cumulative distribution for the same data. Gray bands correspond to the 99th percentile of expected variation from a uniform distribution. (A) Distribution for the DNA binding energy $\Delta\varepsilon_{RA}$ and (B) for the standard deviation σ.

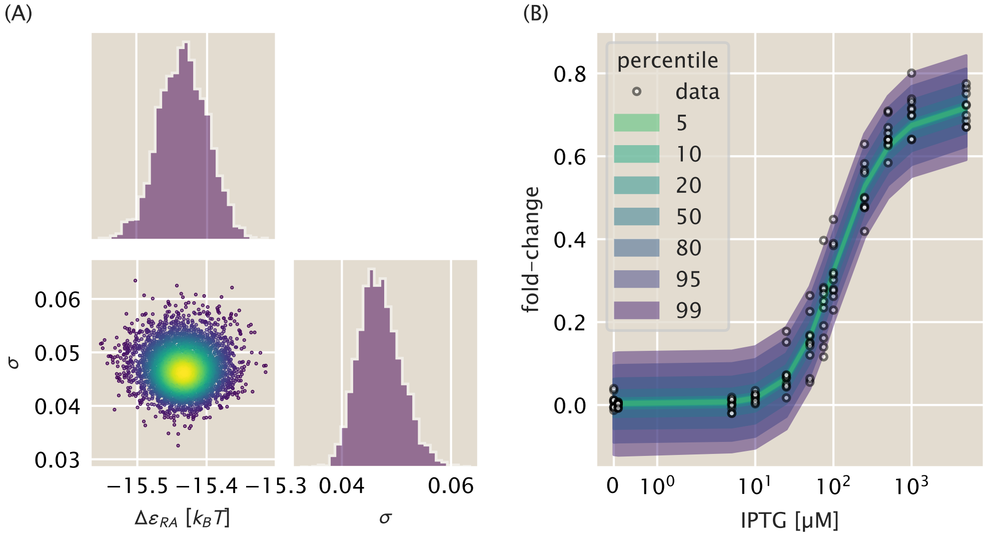

Fig. S7. Markov Chain Monte Carlo (MCMC) samples and posterior predictive check for DNA binding mutant Q18M. (A) Marginal and joint sampling distributions for DNA binding energy $\Delta\varepsilon_{RA}$ and σ. Each point in the joint distribution is a single sample. Marginal distributions for each parameter are shown adjacent to joint distribution. Color in the joint distribution corresponds to the value of the log posterior with the progression of blue to yellow corresponding to increasing probability. (B) The posterior predictive check of model. The measurements of the fold-change in gene expression are shown as black open-faced circles. The percentiles are shown as colored bands and indicate the fraction of simulated data drawn from the likelihood that fall within the shaded region.

4. Inferring the Free Energy From Fold-Change Measurements

In this section, we describe the statistical model to infer the free energy F from a set of fold-change measurements. We follow the same principled workflow as described previously for the DNA binding estimation, including declaration of the generative model, prior predictive checks, simulation based calibration, and posterior predictive checks. Finally, we determine an empirical limit in our ability to infer the free energy and define a heuristic which can be used to identify measurements that are likely inaccurate. To understand the statistical model and the empirical limits of detection, only the subsections *Building A Generative Model* and *Sensitivity Limits and Systematic Errors in Inference* are necessary.

Building A Generative Model. In the main text, we showed that the fold-change equation defined in Eq. (7) can be rewritten in the form of a Fermi function,

$$\text{fold-change} = \frac{1}{1 + e^{-F/k_B T}}, \quad [25]$$

where F corresponds to the free energy difference between the repressor bound and unbound states of the promoter. While the theory prescribes a way for us to calculate the free energy based on our knowledge of the biophysical parameters, we can directly calculate the free energy of a measurement of fold-change by simply rearranging Eq. (25) as

$$F = -k_B T \log\left(\frac{1}{\text{fold-change}} - 1\right). \quad [26]$$

With perfect measurement of the fold-change in gene expression (assuming no experimental or measurement noise), the free energy can be directly calculated. However, actual measurements of the fold-change in gene expression can extend beyond the theoretical bounds of 0 and 1, for which the free energy is mathematically undefined.

As the fold-change measurements between biological replicates are independent, it is reasonable to assume that they are normally distributed about a mean value μ with a standard deviation σ. While the mean value is restricted to the bounds of $[0, 1]$, fold-change measurements outside of these bounds are still possible given that they are distributed about the mean with a scale of σ. Thus, if we have knowledge of the mean fold-change in gene expression about which the observed fold-change is distributed, we can calculate the mean free energy as

$$F = -k_B T \log\left(\frac{1}{\mu} - 1\right). \quad [27]$$

For a given set of fold-change measurements y, we wish to infer the posterior probability distribution for μ and σ, given by Bayes' theorem as

$$g(\mu, \sigma \,|\, y) \propto f(y \,|\, \mu, \sigma) g(\mu) g(\sigma), \quad [28]$$

where we have dropped the normalization constant $f(y)$ and assigned a proportionality between the posterior and joint probability distribution. Given that the measurements are independent, we define the likelihood $f(y \,|\, \mu, \sigma)$ as a normal distribution,

$$f(y \,|\, \mu\, \sigma) \sim \text{Normal}\{\mu, \sigma\}. \quad [29]$$

While the mean μ is restricted to the interval $[0, 1]$, there is no reason *a priori* to think that it is more likely to be closer to either bound. To remain uninformative and be as permissive as possible, we define a prior distribution for μ as a Uniform distribution between 0 and 1,

$$g(\mu) = \begin{cases} \frac{1}{\mu_{\text{max}} - \mu_{\text{min}}} & \mu_{\text{min}} < \mu < \mu_{\text{max}} \\ 0 & \text{otherwise} \end{cases}. \quad [30]$$

Here, $\mu_{\text{min}} = 0$ and $\mu_{\text{max}} = 1$, reducing $g(\mu)$ to 1. For σ, we can again assume a half-normal distribution with a standard deviation of 0.1 as was used for estimating the DNA binding energy [Eq. (20)],

$$g(\sigma) = \text{HalfNormal}\{0, 0.1\}. \quad [31]$$

With a full generative model defined, we can now use prior predictive checks to ensure that our choices of prior are appropriate for the inference.

Prior Predictive Checks. To check the validity of the chosen priors, we pulled 1000 combinations of μ and σ from their respective distributions [Fig. S8(A)] and subsequently drew a set of 10 fold-change values (a number comparable to the number of biological replicates used in this work) from a normal distribution defined by μ and σ. To visualize the range of values generated from these checks, we computed the percentiles of the empirical cumulative distributions of the fold-change values, as can be seen in Fig. S8(C). Approximately 95% of the the generated fold-change measurements were between the theoretical bounds of $[0, 1]$ whereas 5% of the data sets fell outside with the maximum and minimum values extending to ≈ 1.2 and -0.2, respectively. Given our familiarity with these experimental strains and the detection sensitivity of the flow cytometer, these excursions beyond the theoretical bounds agree with our intuition. Satisfied with our choice of prior distributions, we can proceed to check the sensitivity and computational tractability of our model through simulation based calibration.

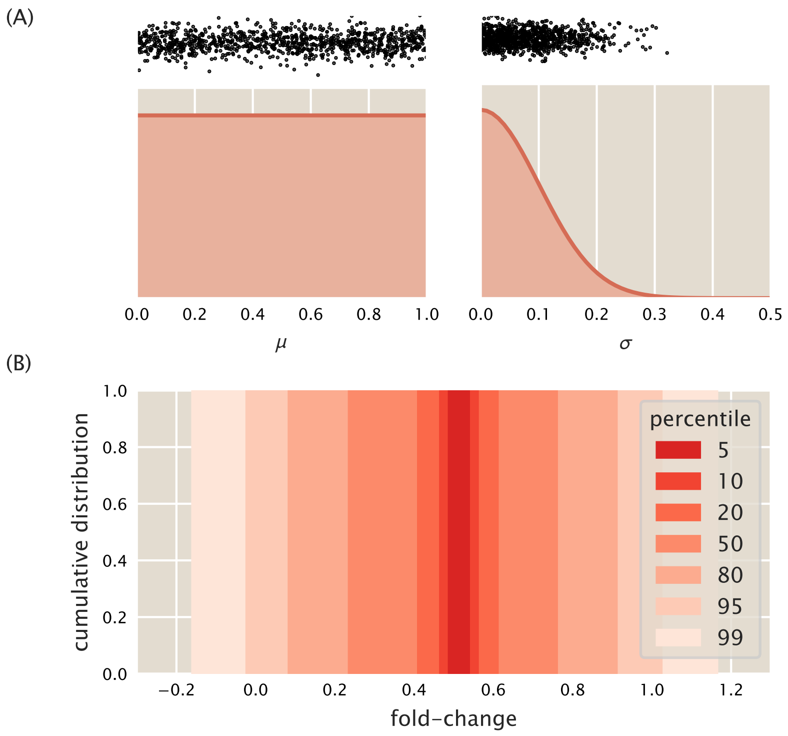

Fig. S8. Prior predictive checks for inference of the mean fold-change. (A) The prior distributions for μ (left) and σ (right). The vertical axis is proportional to the probability of the value. Black points above distributions correspond to the values used to perform the prior predictive checks. (B) Percentiles of the data generated for each draw from the prior distributions shown as a cumulative distribution. Percentiles were calculated for 1000 generated data sets, each with 10 fold-change measurements drawn from the likelihood given the drawn values of μ and σ.

Simulation Based Calibration. To ensure that the parameters can be estimated with confidence, we sampled the posterior distribution of μ and σ for each data set generated from the prior predictive checks. For each inference, we computed the z-score and shrinkage for each parameter, shown in Fig. S9(A). For both parameters, the z-scores are approximately centered about zero, indicating that the posteriors concentrate about the ground truth value of the parameter. The z-scores for σ [black points in Fig. S11(A)] appear to be slightly off centered with more negative values than positive. This suggests that σ is more likely to be slightly overestimated in some cases. The shrinkage parameter for μ (red points) is very tightly distributed about 1.0, indicating that the prior is being strongly informed by the data. The shrinkage is more broadly distributed for for σ with a minimum value of ≈ 0.5. However, the median shrinkage for σ is ≈ 0.9, indicating that half of the inferences shrank the prior distribution by at least 90%. While we could revisit the model to try and improve the shrinkage values, we are more concerned with μ which shows high shrinkage and zero-centered z-scores.

To ensure that the model is computationally tractable, we computed the rank statistic of each parameter for each inference. The empirical cumulative distributions for μ (black) and σ (red) can be seen in Fig. S9(B). Both distributions appear to be uniform, falling within the 99th percentile of the variation expected from a true uniform distribution. This indicates that the self-consistency relation defined by Eq. (22) holds for this statistical model. With a computationally tractable model in hand, we can now apply the statistical model to our data and verify that data sets drawn from the data-conditioned posterior are indistinguishable from the experimental measurements.

Posterior Predictive Checks. The same statistical model was applied to every unique set of fold-change measurements used in this work. Here, we focus only on the set of fold-change measurements for the double mutant Y17I-Q291V at 50 μM IPTG. The samples from the posterior distribution conditioned on this dataset can be seen in Fig. S10(A). The joint distribution, shown in the lower left-hand corner, appears fairly symmetric, indicating that μ and σ are independent. There is a slight asymmetry in the sampling of σ, which can be more clearly seen in the corresponding marginal distribution to the right of the joint distribution.

For each MCMC sample of μ and σ, we drew 10 samples from a normal distribution defined by these parameters. From this collection of data sets, we computed the percentiles of the empirical cumulative distribution and plotted them over the data, as can be seen in Fig. S10 (B). We find that the observed data falls within the 99th percentile of the generated data sets. This illustrates that the model can produce data which is identically distributed to the actual experimental measurements, validating our choice of statistical model.

Sensitivity Limits and Systematic Errors in Inference. Considering the results from the prior predictive checks, simulation based calibration, and posterior predictive checks, we can say that the statistical model for inferring μ and σ fold-change from a collection

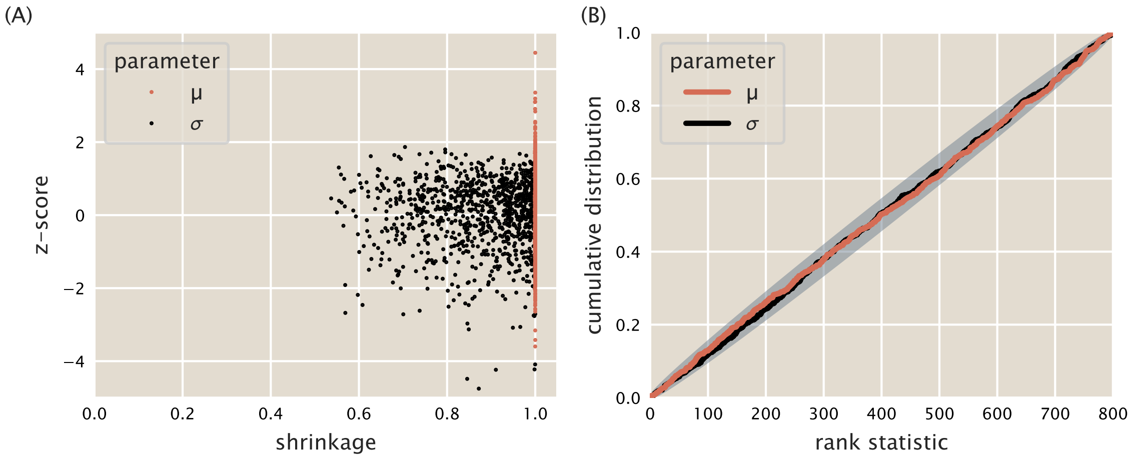

Fig. S9. Sensitivity measurements and rank statistic distribution of the statistical model estimating μ and σ. (A) Posterior z-score of each inference plotted against the posterior shrinkage factor for the parameters μ (red points) and σ (black points). (B) Distribution of rank statistics for μ (red) and σ (black). Gray envelope represents the 99th percentile of a true uniform distribution.

of noisy fold-change measurements is valid and computationally tractable. Upon applying this model to the experimental data of the wild-type strain (where the free energy is theoretically known), we observed that systematic errors arise when the fold-change is exceptionally high or low, making the resulting inference of the free energy inaccurate.

To elucidate the source of this systematic error, we return to a simulation based approach in which the true free energy is known [black points in Fig. S11(A)]. For a range of free energies, we computed the theoretical fold-change prescribed by Eq. (25). For each free energy value, we pulled a value for σ from the prior distribution defined in Eq. (20) and generated a data set of 10 measurements by drawing values from a normal distribution defined by the true fold-change and the drawn value of σ [red points in Fig. S11(A)]. We then sampled the statistical model over these data and inferred the mean fold-change μ [blue points in Fig. S11(A)]. By eye, the inferred points appear to collapse onto the master curve, in many cases overlapping the true values. However, the points with a free energy less than $\approx -2\,k_BT$ and greater than $\approx 2\,k_BT$ are slightly above or below the master curve, respectively. This becomes more obvious when the inferred free energy is plotted as a function of the true free energy, shown in Fig. S11(B). Points in which the difference between μ and the neearest boundary (0 or 1) is less than the value of σ are shown as purple or green. When this condition is met, the inferred mean free energy strays from the true value, introducing a systematic error. This suggests that the spread of the fold-change measurements sets the detection limit of fold-change close to either boundary. Thus, the narrower the spread in the fold-change the better the estimate of the fold-change near the boundaries.

These systematic errors can be seen in experimental measurements of the wild-type repressor. Data from Razo-Mejia et al. 2018(2) in which the IPTG titration profiles of seventeen different bacterial strains were measured is shown collapsed onto the master curve in Fig. S11(C) as red points. Here, each point corresponds to a single biological replicate. The inferred mean fold-change μ and 95% credible regions are shown as purple, blue, or green points. The color of these points correspond to the relative value of μ or $1-\mu$ to σ. The discrepancy between the predicted and inferred free energy of each measurement set can be seen in Fig. S11(D). The significant deviation from the predicted and inferred free energy occurs past the detection limit set by σ. In this work, we therefore opted to not display inferred free energies at the extrema where the inferred fold-change was closer to the boundaries than the correspoding standard deviation, as it reflects limitations in our measurement rather than a deviation from the theoretical predictions.

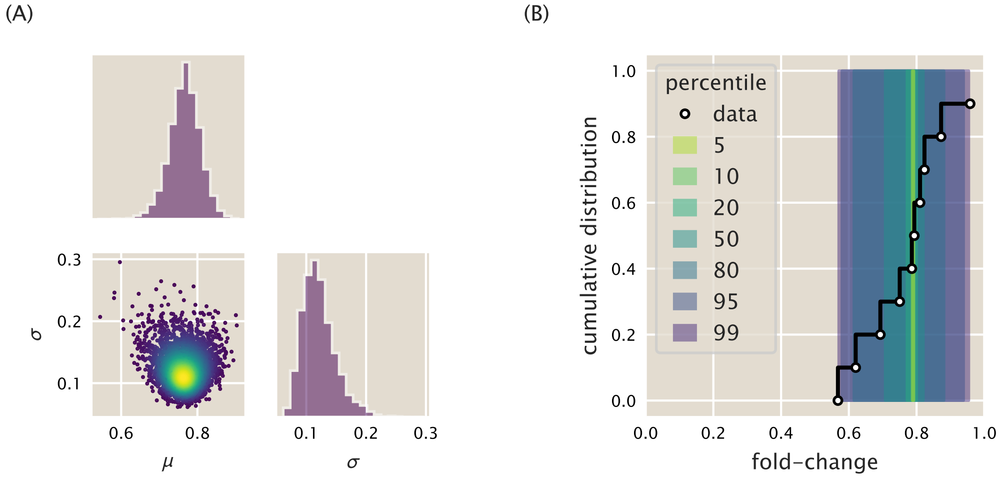

Fig. S10. MCMC sampling output and posterior predictive checks of the statistical model for the mean fold-change μ and standard deviation σ. (A) Corner plot of sampling output. The joint distribution between σ and μ is shown in the lower left hand corner. Each point is an individual sample. Points are colored by the value of the log posterior with increasing probability corresponding to transitions from blue to yellow. Marginal distributions for each parameter are shown adjacent to the joint distribution. (B) Percentiles of the cumulative distributions from the posterior predictive checks are shown as shaded bars. Data on which the posterior was conditioned are shown as white circles connected by black lines.

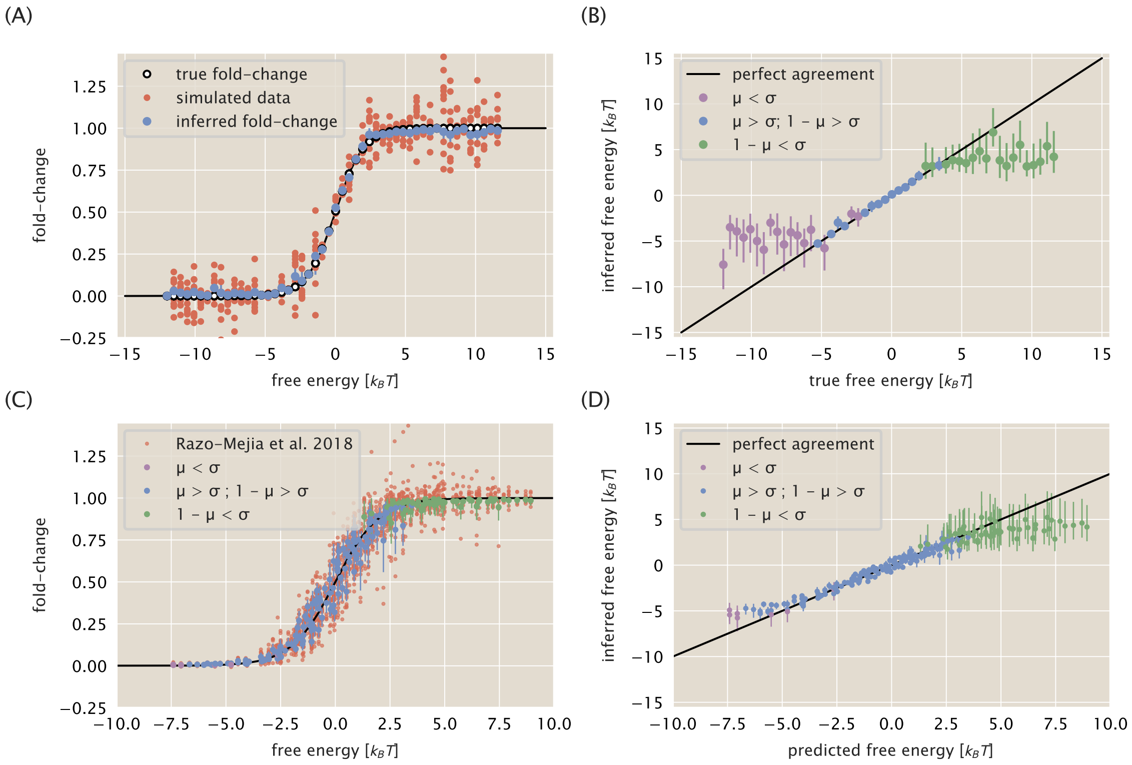

Fig. S11. Identification of systematic error in simulated and real data when considering the free energy. (A) The true fold-change (black open circles), simulated fold-change distribution (red points), and inferred mean fold-change (blue) is plotted as a function of the true free energy. Error bars on inferred fold-change correspond to the 95% credible region of the mean fold-change μ. (B) Inferred free energy plotted as a function of the true free energy. Black line indicates perfect agreement between the ground truth free energy and inferred free energy. Blue points correspond to the inferred free energy where the median values of the parameters satisfy the condition $\mu > \sigma$ and $1 - \mu > \sigma$. Purple points correspond to the inferred mean fold-change $\mu < \sigma$. Green points correspond to those where the inferred mean fold-change $1 - \mu < \sigma$. Error bars correspond to the bounds of the 95% credible region. (C) Biological replicate data from Razo-Mejia et al. 2018 (2) (red points) plotted as a function of the theoretical free energy. Inferred mean fold-change μ and the 95% credible region are shown as blue points. Purple and green points are colored by the same conditions as in (B). (D) Inferred free energy as a function of the predicted free energy colored by the satisfied condition. Error bars are the bounds of the 95% credible region. All inferred values in (A - D) are the median values of the posterior distribution.

5. Additional Characterization of DNA Binding Mutants

In the main text, we estimated the DNA binding energy o f each mutant using the mutant strains that had approximately 260 repressors per cell. In this section, we examine the effect of the choice of fit strain on the predictions of both the induction profiles and ΔF for each DNA binding domain mutant.

We applied the statistical model derived in Section 3 for each unique strain of the DNA binding mutants and estimated the DNA binding energy. The median of the posterior distribution along with the upper and lower bounds of the 95% credible region are reported in Table S1. We found that the choice of fitting strain did not strongly influence the estimate of the DNA binding energy. The largest deviations appear for the weakest binding mutants paired with the lowest repressor copy number. In these cases, such as for Q18A, the difference in binding energy between the repressor copy numbers is $\approx 1\, k_BT$ which is small compared to the overall DNA binding energy. Using these energies, we computed the predicted induction profiles of each mutant with different repressor copy numbers, shown in Fig. S12. In this plot, the rows correspond to the repressor copy number of the strain used to estimate the DNA binding energy. The columns correspond to the repressor copy number of the predicted strains. The diagonals, shaded in grey, show the induction profile of the fit strain along with the corresponding data. In all cases, we find that the predicted profiles are relatively accurate with the largest deviations resulting from using the lowest repressor copy number as the fit strain.

The predicted change in free energy ΔF using each fit strain can be seen in Fig. S13. In this figure, the rows represent the repressor copy number of the strain to which the DNA binding energy was fit whereas the columns correspond to each mutant. In each plot, we have shown the data for all repressor copy numbers with the fit strain represented by white filled circles. Much as for the induction profiles, we see little difference in the predicted ΔF for each strain, all of which accurately describe the inferred free energies. The ability to accurately predict the majority of the induction profiles of each mutant with repressor copy numbers ranging over two orders of magnitude strengthens our assessment that for these DNA binding domain mutations, only the DNA binding energy is modified.

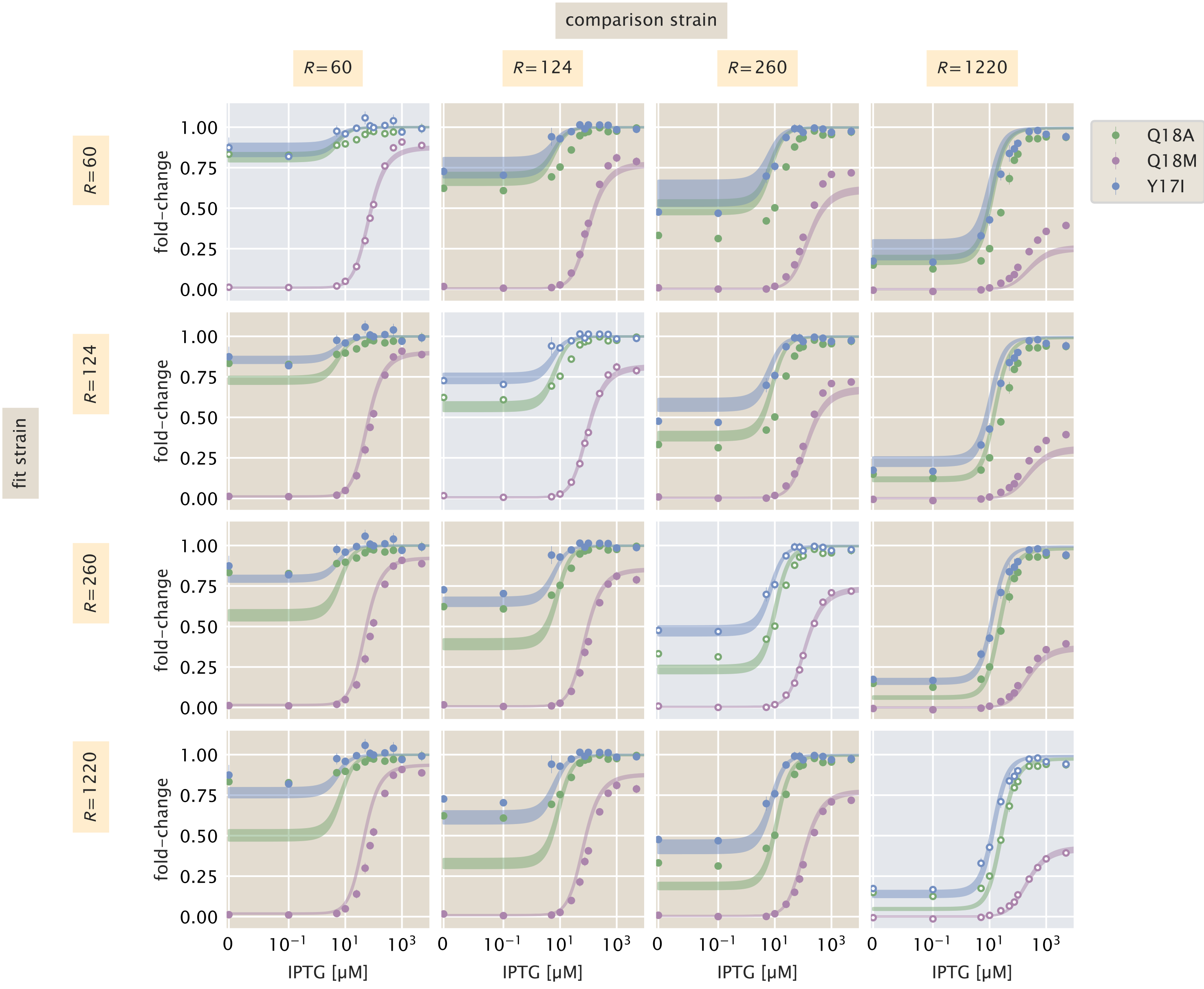

Fig. S12. Pairwise comparisons of DNA binding mutant induction profiles. Rows correspond to the repressor copy number of the strain used to estimate the DNA binding energy for each mutant. Columns correspond to the repressor copy number of the strains that are predicted. Diagonals in which the data used to estimate the DNA binding energy are shown with a gray background.

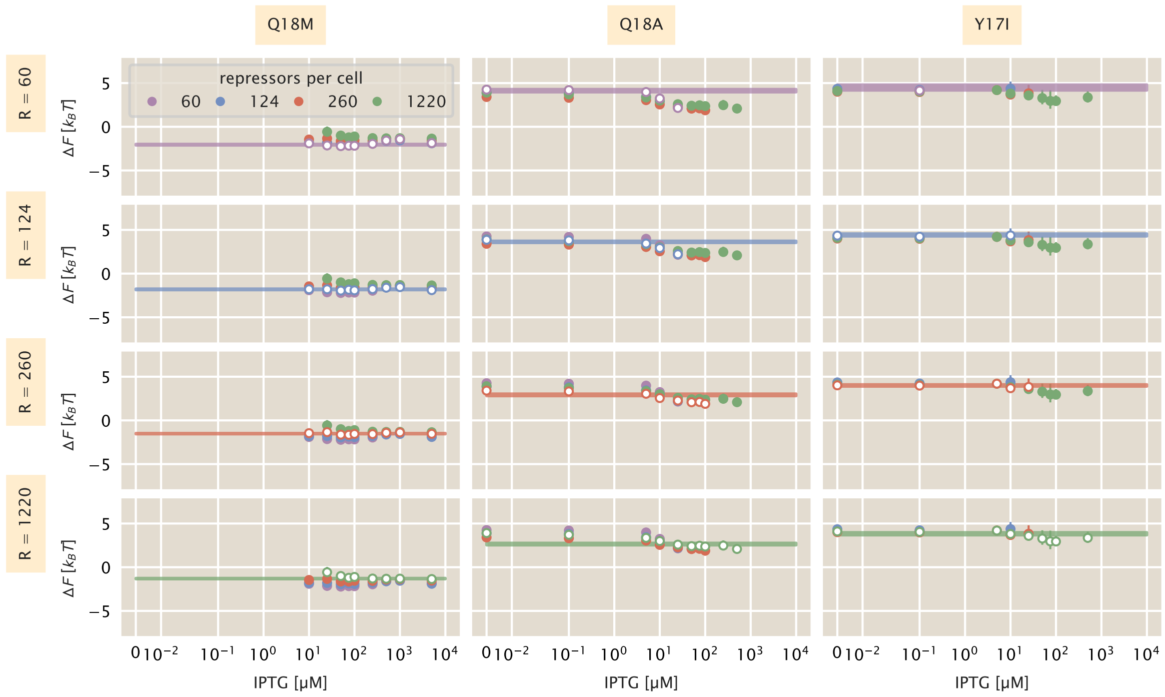

Fig. S13. Dependence of fitting strain on ΔF predictions of DNA binding domain mutants. Rows correspond to the repressor copy number used to estimate the DNA binding energy. Columns correspond to the particular mutant. Colored lines are the bounds of the 95% credible region of the predicted ΔF. Open face points indicate the strain to which the DNA binding energy was fit.

Table S1. Estimated DNA binding energy for DNA binding domain mutants with different repressor copy numbers. Median of the posterior distribution with the upper and lower bounds of the 95% credible region are reported.

Mutant	Repressors	DNA Binding Energy $[k_BT]$
Q18A	60	$-9.8^{+0.2}_{-0.2}$
	124	$-10.3^{+0.1}_{-0.1}$
	260	$-11.0^{+0.1}_{-0.1}$
	1220	$-11.3^{+0.1}_{-0.1}$
Q18M	60	$-15.83^{+0.08}_{-0.08}$
	124	$-15.7^{+0.1}_{-0.1}$
	260	$-15.43^{+0.07}_{-0.06}$
	1220	$-15.27^{+0.07}_{-0.07}$
Y17I	60	$-9.4^{+0.3}_{-0.3}$
	124	$-9.5^{+0.1}_{-0.1}$
	260	$-9.9^{+0.1}_{-0.1}$
	1220	$-10.1^{+0.2}_{-0.2}$

6. Bayesian Parameter Estimation for Inducer Binding Domain Mutants

In the main text, we put forward two naïve hypotheses for which parameters of Eq. (7) are affected by mutations in the inducer binding domain of the repressor. The first hypothesis was that only the inducer dissociation constants, K_A and K_I, were perturbed from their wild-type values. Another hypothesis was that the inducer dissociation constants were affected in addition to the energetic difference between the active and inactive states of the repressor, $\Delta\varepsilon_{AI}$.

In this section, we first derive the statistical model for each hypothesis and then perform a series of diagnostic tests that expose the inferential limitations of each model. With well calibrated statistical models, we then apply each to an induction profile of the inducer binding mutant Q291K and assess the validity of each hypothesis. To understand the statistical models for each hypothesis, only the subsection *Building A Generative Statistical Model* is necessary.

Building a Generative Statistical Model. For both hypotheses, we assume that the underlying physical model defined in Eq. (7) is the same while a subset of the parameters are modified. As the fold-change measurements for each biological replicate are statistically independent, we can assume that they are normally distributed about the theoretical fold-change value. Thus, for each model, we must include a parameter σ which is the standard deviation of the distribution of fold-change measurements. For the first hypothesis, in which only K_A and K_I are changed, we are interested in sampling the posterior distribution

$$g(K_A, K_I, \sigma \,|\, y) \propto f(y \,|\, K_A, K_I, \sigma) g(K_A) g(K_I) g(\sigma), \quad [32]$$

where y corresponds to the set of fold-change measurements. In the above model, we have assumed that the priors for K_A and K_I are independent. It is possible that it is more appropriate to assume that they are dependent and that a single prior distribution captures both parameters, $g(K_A, K_I)$. However, assigning this prior is more difficult and requires strong knowledge *a priori* about the relationship between them. Therefore, we continue under the assumption that the priors are independent.

The generic posterior given in Eq. (32) can be extended to evaluate the second hypothesis in which $\Delta\varepsilon_{AI}$ is also modified,

$$g(K_A, K_I, \Delta\varepsilon_{AI}, \sigma \,|\, y) \propto f(y \,|\, K_A, K_I, \Delta\varepsilon_{AI}, \sigma) g(K_A) g(K_I) g(\Delta\varepsilon_{AI}) g(\sigma) \quad [33]$$

where we have included $\Delta\varepsilon_{AI}$ as an estimated parameter and assigned a prior distribution.

As we have assumed that the fold-change measurements across replicates are independent and normally distributed, the likelihoods for each hypothesis can be written as

$$f(y \,|\, K_A, K_I, \sigma) \sim \text{Normal}\{\mu(K_A, K_I), \sigma\}, \quad [34]$$

for the first hypothesis and

$$f(y \,|\, K_A, K_I, \Delta\varepsilon_{AI}, \sigma) \sim \text{Normal}\{\mu(K_A, K_I, \Delta\varepsilon_{AI}), \sigma\}, \quad [35]$$

for the second. Here, we have assigned $\mu(\ldots)$ as the mean of the normal distribution as a function of the parameters defined by our fold-change equation, Eq. (7).

With a likelihood distribution in hand, we now turn toward assigning functional forms to each prior distribution. As we have used in the previous sections [Sec. 3 and Sec. 4], we can assign a half-normal prior for σ with a standard deviation of 0.1, namely,

$$g(\sigma) \sim \text{HalfNormal}\{0, 0.1\}. \quad [36]$$

It is important to note that the inducer dissociation constants K_A and K_I are scale invariant, meaning that a change from 0.1 μM to 1 μM yields a decrease in affinity equal to a change from 10 μM to 100 μM. As such, it is better to sample the dissociation constants on a logarithmic scale. We can assign a log normal prior for each dissociation constant as

$$g(K_A) = \frac{1}{K_A\sqrt{2\pi\phi^2}} \exp\left[-\frac{(\log\frac{K_A}{1\,\mu\text{M}} - \mu_{K_A})^2}{2\phi^2}\right], \quad [37]$$

or with the short-hand notion of

$$g(K_A) \sim \text{LogNormal}\{\mu_{K_A}, \phi\} \quad [38]$$

For K_A, we assigned a mean $\mu_{K_A} = 2$ and a standard deviation $\phi = 2$. For K_I, we chose a mean of $\mu_{K_I} = 0$ and $\phi = 2$, capturing our prior knowledge that $K_A > K_I$ for the wild-type LacI. While the prior distributions are centered differently, they both show extensive overlap, permitting mutations in which $K_A < K_I$. For $\Delta\varepsilon_{AI}$, we assign a normal distribution of the prior centered at 0 with a standard deviation of $5\,k_BT$,

$$g(\Delta\varepsilon_{AI}) \sim \text{Normal}\{0, 5\}. \quad [39]$$

This permits values of $\Delta\varepsilon_{AI}$ that are above or below zero, meaning that the inactive state of the repressor can be either more or less energetically favorable to the active state. A standard deviation of $5\,k_BT$ permits a wide range of energies with $+5\,k_BT$ and $-5\,k_BT$ corresponding to $\approx$ 99.5% and $\approx$ 0.5% of the repressors being active in the absence of inducer, respectively.

Prior predictive checks. To ensure that these choices of prior distributions are appropriate, we performed prior predictive checks for each hypothesis as previously described in Section 3. We drew 1000 values from the prior distributions shown in Fig. S14(A) for K_A, K_I, and $\Delta\varepsilon_{AI}$. Using the draws from the K_A, and K_I priors alone, we generated datasets of ≈ 70 measurements. The percentiles of the fold-change values drawn for the 1000 simulations is shown in the top panel of Fig. S14(B).

It can be seen that in the absence of inducer, the fold-change values are close to zero and are with distributed about the leakiness value due to σ. This is in contrast to the data sets generated when $\Delta\varepsilon_{AI}$ is permitted to vary along with K_A and K_I. In the bottom panel of Fig. S14(B), the fold-change when $c = 0$ can extend above 1.0 which is possible only when $\Delta\varepsilon_{AI}$ is included, which sets what fraction of the repressors is active. Under both hypotheses, the 99th percentile of the fold-change extends to just above 1 or just below 0, which matches our intuition of how the data should behave. Given these results, we are satisfied with these choices of priors and continue onto the next level of calibration of our model.

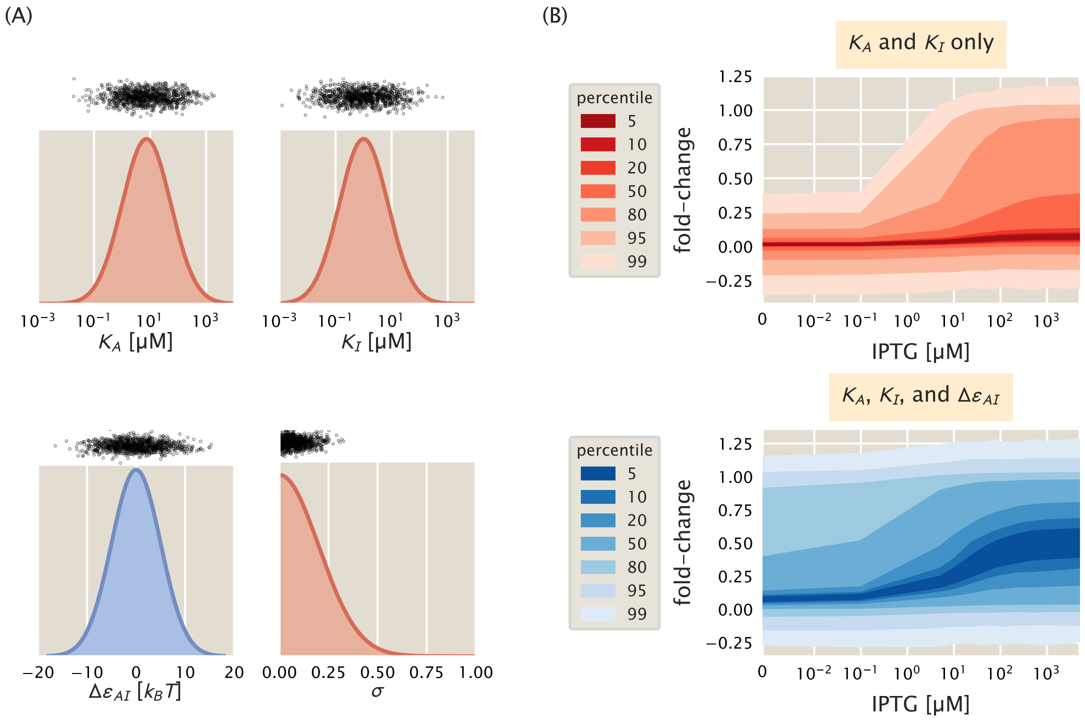

Fig. S14. Prior predictive checks for two hypotheses of inducer binding domain mutants. (A) Probability density functions for K_A, K_I, $\Delta\varepsilon_{AI}$, and σ. Black points correspond to draws from the distributions used for prior predictive checks. (B) Percentiles of the simulated data sets using draws from the K_A and K_I distributions only (top, red bands) and using draws from K_A, K_I, and $\Delta\varepsilon_{AI}$ (bottom, blue bands).

Simulation Based Calibration. With an appropriate choice of priors, we turn to simulation based calibration to root out any pathologies lurking in the model itself or the implementation through MCMC. For each parameter under each model, we compute the z-score and shrinkage of each inference, shown in Fig. S15. Under the first hypothesis in which K_A and K_I are the only perturbed parameters [Fig. S15(A)], we see all parameters have $z-$scores clustered around 0, indicating that the value of the ground-truth is being accurately estimated through the inference. While the shrinkage for σ is close to 1 (indicating the prior is being informed by the data), the shrinkage for K_A and K_I is heavily tailed with some values approaching zero. This is true for both statistical models, indicating that for some values of K_A and K_I, the parameters are difficult to pin down with high certainty. In the application of these models to data, this will be revealed as large credible regions in the reported parameters. Under the second hypothesis in which all allosteric parameters are allowed to change, we see moderate shrinkage for $\Delta\varepsilon_{AI}$ [purple points in Fig. S15(B)] with the minimum shrinkage being around 0.5. The samples resulting in low shrinkage correspond to values of $\Delta\varepsilon_{AI}$ that are highly positive or highly negative, in which small changes in the active fraction of repressors cannot be accurately measured through our model. However, the median shrinkage for $\Delta\varepsilon_{AI}$ is approximately 0.92, meaning that the the data highly informed the prior distributions for the majority of the inferences. The rank distributions for all parameters under each model appear to be highly uniform, indicating that both statistical models are computationally tractable.

With knowledge of the caveats of estimating K_A and K_I for both models, we proceed with our analysis and examine how accurately these models can capture the phenomenology of the data.

Posterior Predictive Checks. With a properly calibrated statistical model for each hypothesis, we now apply it to a representative dataset. While each model was applied to each inducer binding domain mutant, we only show the application to the mutant Q291K with 260 repressors per cell paired with the native *lac* operator O2.

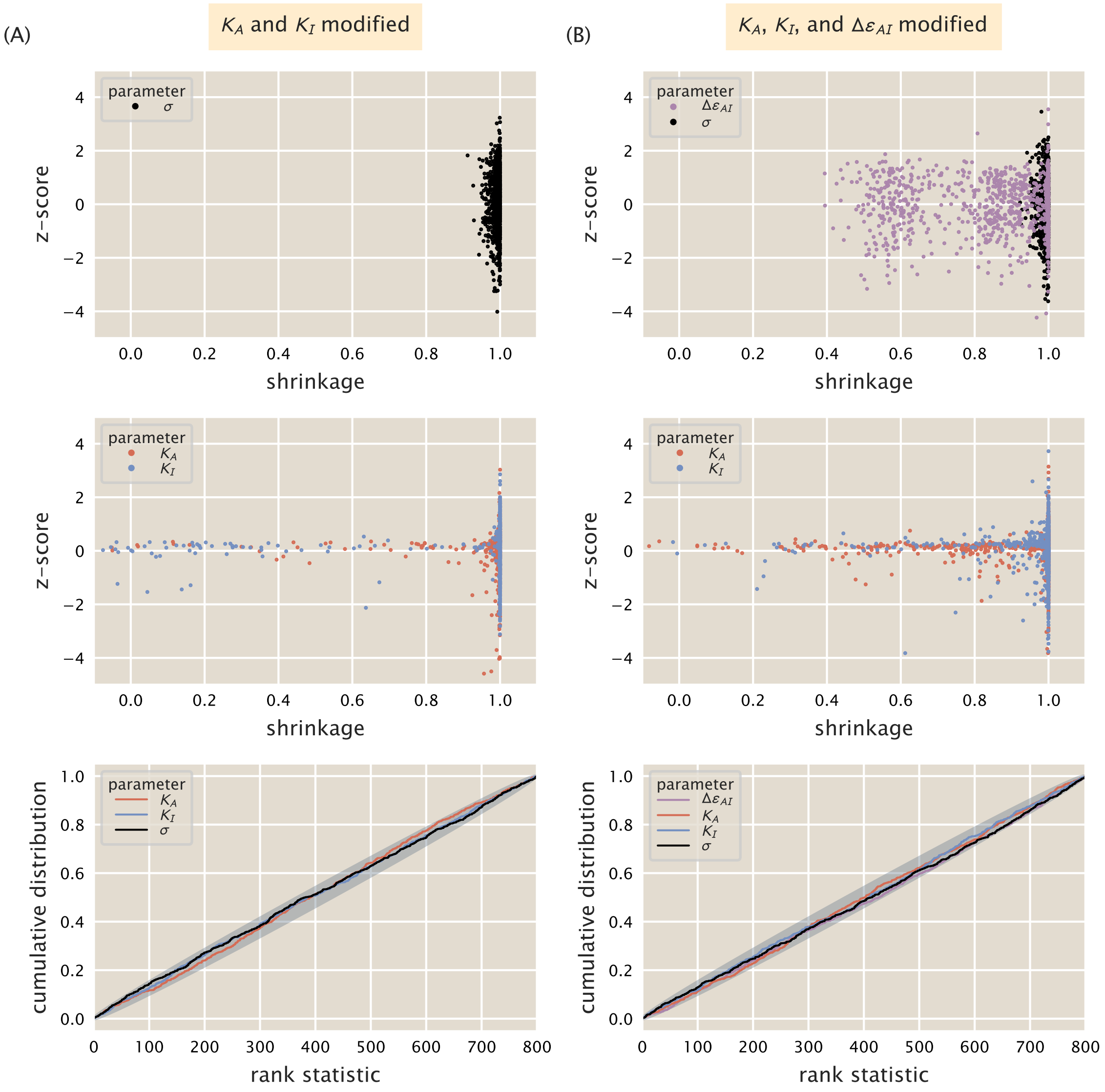

Fig. S15. Simulation based calibration of statistical models for inducer binding domain mutants. (A) Sensitivity statistics and rank distribution for a statistical model in which K_A and K_I are the only parameters permitted to vary. (B) Sensitivity statistics and rank distribution for a model in which all allosteric parameters K_A, K_I, and $\Delta\varepsilon_{AI}$ are allowed to be modified by the mutation. Gray envelope in the bottom plots correspond to the 99th percentile of variation expected from a true uniform distribution.

The results from applying the statistical model in which only K_A and K_I can change is shown in Fig. S16. The joint and marginal distributions for each parameter [Fig. S16(A)] reveal a strong correlation between K_A and K_I whereas all other parameters are symmetric and independent. While the joint and marginal distributions look well behaved, the percentiles of the posterior predictive checks [Fig. S16(B)] are more suspect. While all data falls within the 95th percentile, the overall trend of the data is not well predicted. Furthermore, the percentiles expand far below zero, indicating that the sampling of σ is compensating for the leakiness in the data being larger than it should be if only K_A and K_I were the changing parameters.

We see significant improvement when $\Delta\varepsilon_{AI}$ is permitted to vary in addition to K_A and K_I. Fig. S17(A) shows the joint and marginal distributions between all parameters from the MCMC sampling. We still see correlation between K_A and K_I, although it is not as strong as in the case where they are the only parameters allowed to change due to the mutation. We also see that the marginal distribution for σ has shrunk significantly compared to the marginal distribution in Fig. S16(A). The percentiles of the posterior predictive checks, shown in Fig. S17(B) are much more in line with the experimental measurements, with the 5th percentile following the data for the entire induction profile.

In this section we have presented two hypotheses for the minimal parameter set needed to describe the inducer binding mutations, derived a statistical model for each, thoroughly calibrated its behavior, and applied it to a representative data set. The posterior predictive checks [Fig. S16 and Fig. S17] help us understand which hypothesis is more appropriate for that particular mutant. The incredibly wide percentiles and significant change in the leakiness that result from a model in which only K_A and K_I are perturbed suggests that more than those two parameters should be changing. We see significant improvement in the description of the data when $\Delta\varepsilon_{AI}$ is altered, indicating that it is the more appropriate hypothesis of the two.

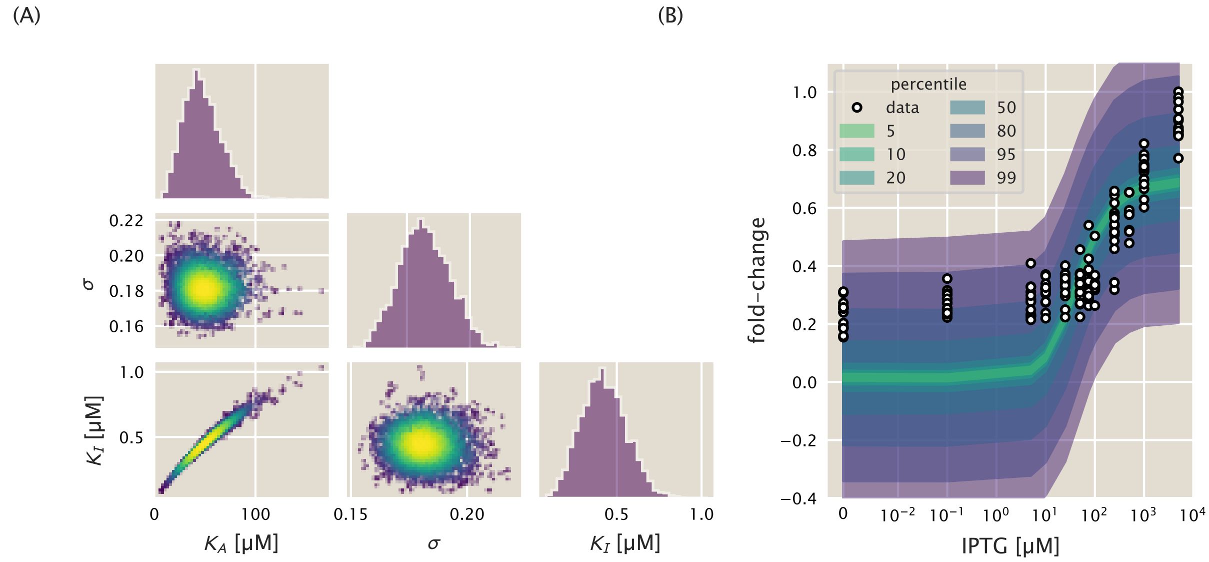

Fig. S16. Posterior predictive checks for inducer binding domain mutants where only K_A and K_I are changed. (A) MCMC sampling output for each parameter. Joint distributions are colored by the value of the log posterior with increasing probability corresponding to transition from blue to yellow. (B) Percentiles of the data generated from the likelihood distribution for each sample of K_A, K_I, and σ. Overlaid points are the experimentally observed measurements.

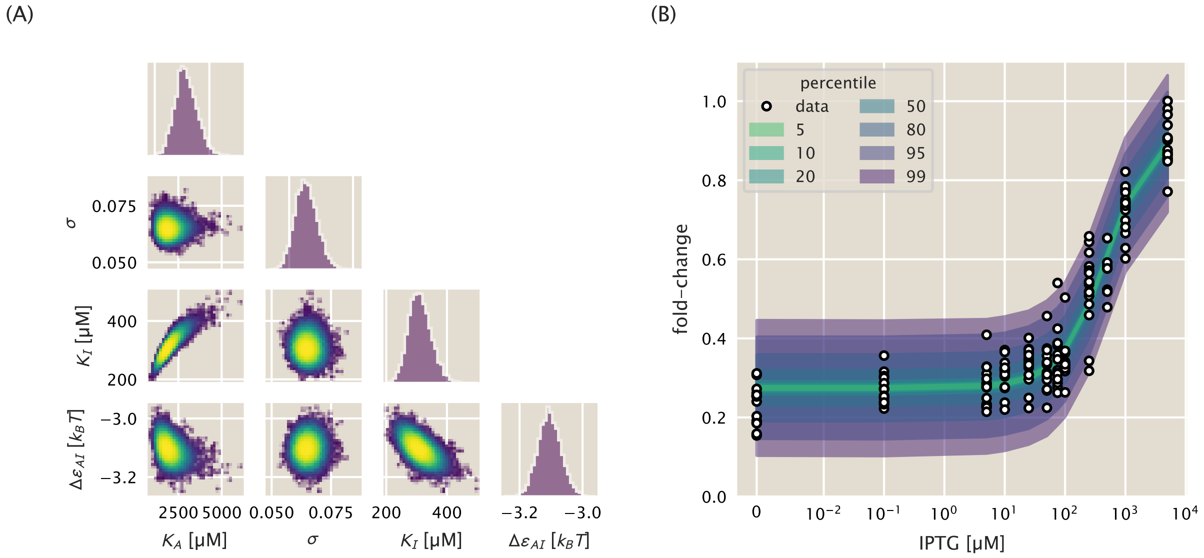

Fig. S17. Posterior predictive checks for inducer binding domain mutants where all allosteric parameters can change. (A) MCMC sampling output for all parameters. Joint distributions are colored by the value of the log posterior with increasing probability corresponding to the transition from blue to yellow. Marginal distributions are shown adjacent to each joint distribution. (B) Percentiles of the data generated from the likelihood for each sample of K_A, K_I, $\Delta\varepsilon_{AI}$, and σ. The corresponding experimental data for Q291K are shown as black open-faced circles.

7. Additional Characterization of Inducer Binding Domain Mutants

To predict the induction profiles of the inducer binding mutants, we used only the induction profile of each mutant paired with the native O2 *lac* operator to infer the parameters. Here, we examine the influence the choice of fit strain has on the predictions of the induction profiles and ΔF for each mutant.

In the main text, we dismissed the hypothesis that only K_A and K_I were changing due to the mutation and based the fit to a single induction profile. In Fig. S18, the fits and predictions for each mutant paired with each operator sequence queried. Here, the rows correspond to the operator sequence of the fit strain while the columns correspond to the operator sequence of the predicted strain. The diagonals, colored in gray, show the fit induction profiles and the corresponding data. Regardless of the choice of fit strain, the predicted induction profiles of the repressor paired with the O3 operator are poor, with the leakiness in each case being significantly underestimated. We also see that fitting to O3 results in poor predictions with incredibly wide credible regions for the other two operators. In Razo-Mejia et al. 2018 (2), we also found that fitting K_A and K_I to the induction profile of O3 generally resulted in poor predictions of the other strains with comparably wide credible regions.

When $\Delta\varepsilon_{AI}$ is included as a parameter, however, the predictive power is improved for all three operators, as can be seen in Fig. S19. While the credible regions are still wide when fit to the O3 operator, they are much narrower than under the first hypothesis. We emphasize that we are able to accurately predict the leakiness of nearly every strain by redetermining $\Delta\varepsilon_{AI}$ whereas the leakiness was not predicted when only K_A and K_I were considered. Thus, we conclude that all three allosteric parameters K_A, K_I, and $\Delta\varepsilon_{AI}$ are modified for these four inducer binding domain mutations. The values of the inferred parameters are reported in Table S2.

We also examined the effect the choice of fit strain has on the predicted ΔF, shown in Fig. S20. We find that the predictions agree with the data regardless of the choice of fit strain. One exception is the prediction of the Q291K ΔF when the parameters fit to the O3 induction profile are used. As the induction profile for Q291K paired with O3 is effectively flat at a fold-change of 1, it is difficult to properly estimate the parameters of our sigmoidal function. We note all measurements of ΔF for Q291K are described by using either the parameters fit to either O1 or O3 induction profiles, suggesting that the choice of fit strain makes little difference.

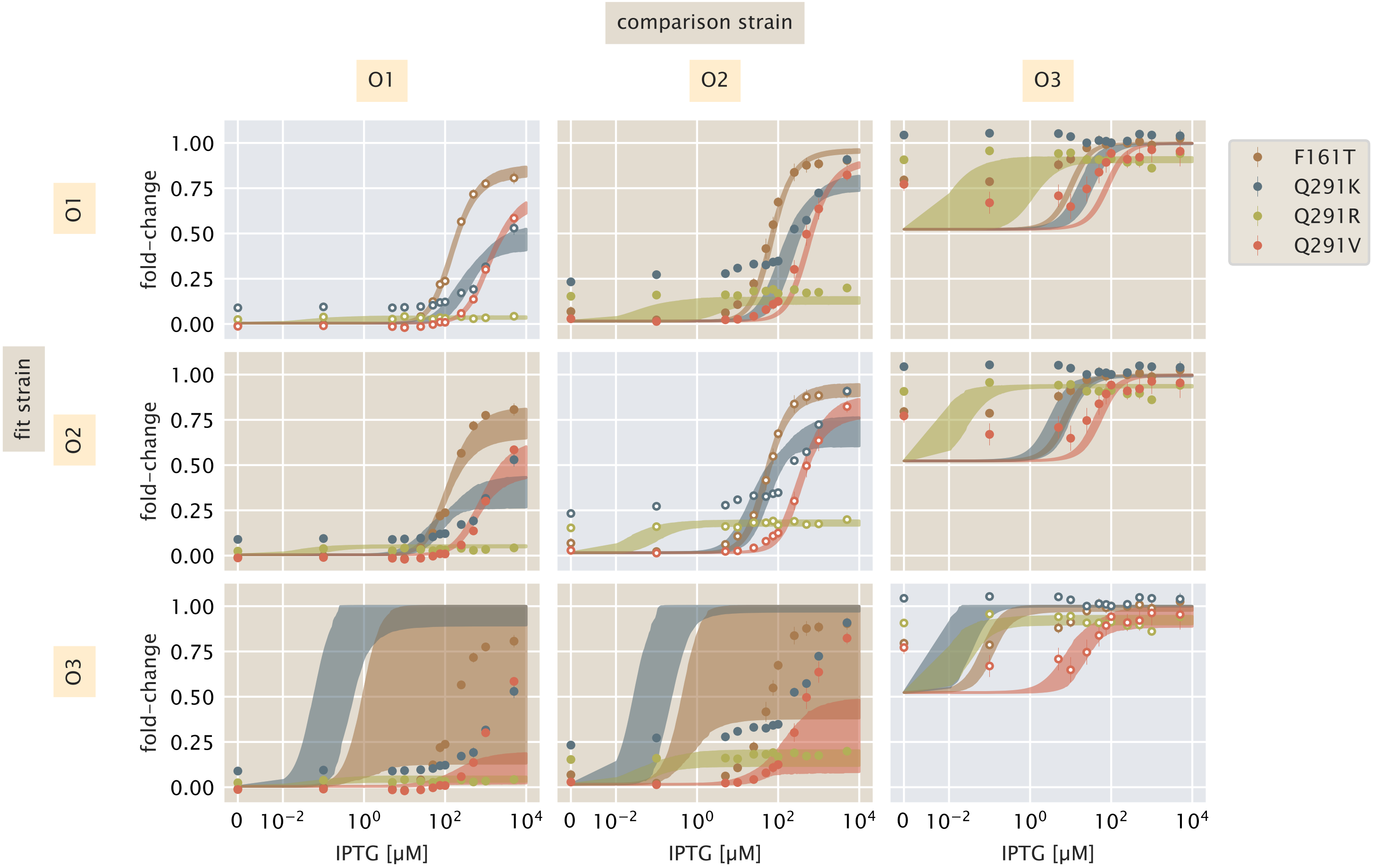

Fig. S18. Pairwise comparison of fit strain versus predictions assuming only K_A and K_I are influenced by the mutation. Rows correspond to the operator sequence of the strain used for the parameter inference. Columns correspond to the operator sequence of the predicted strain. Colors identify the mutation. Diagonal positions (gray background) show the induction fit strain and profiles.

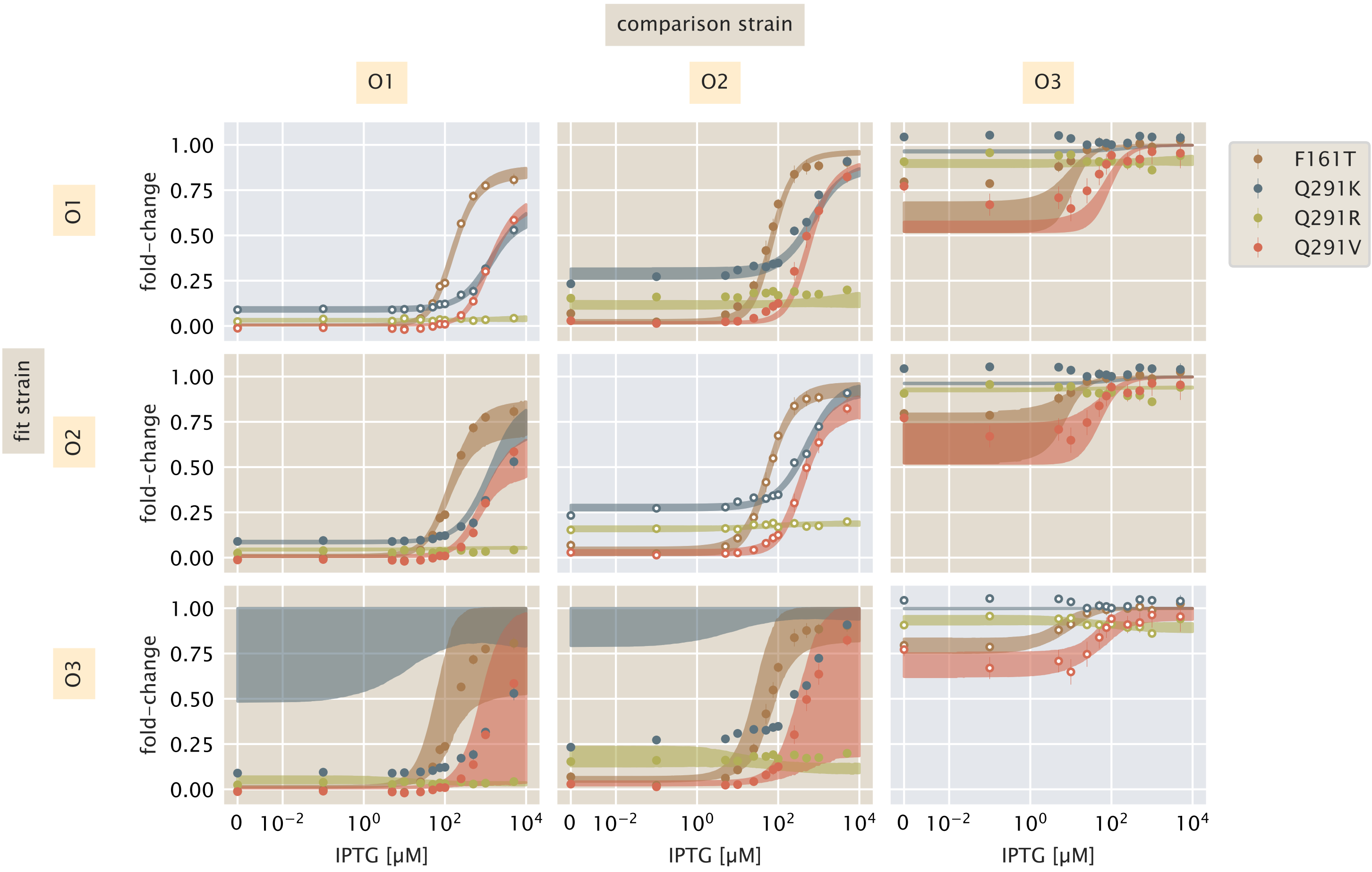

Fig. S19. Pairwise comparison of fit strain versus predictions assuming all allosteric parameters are affected by the mutation. Rows correspond to the operator of the strain used to fit the parameters. Columns correspond to the operator of the strains whose induction profile is predicted. Mutants are identified by color. Diagonals (gray background) show the induction profiles of the strain to which the parameters were fit.

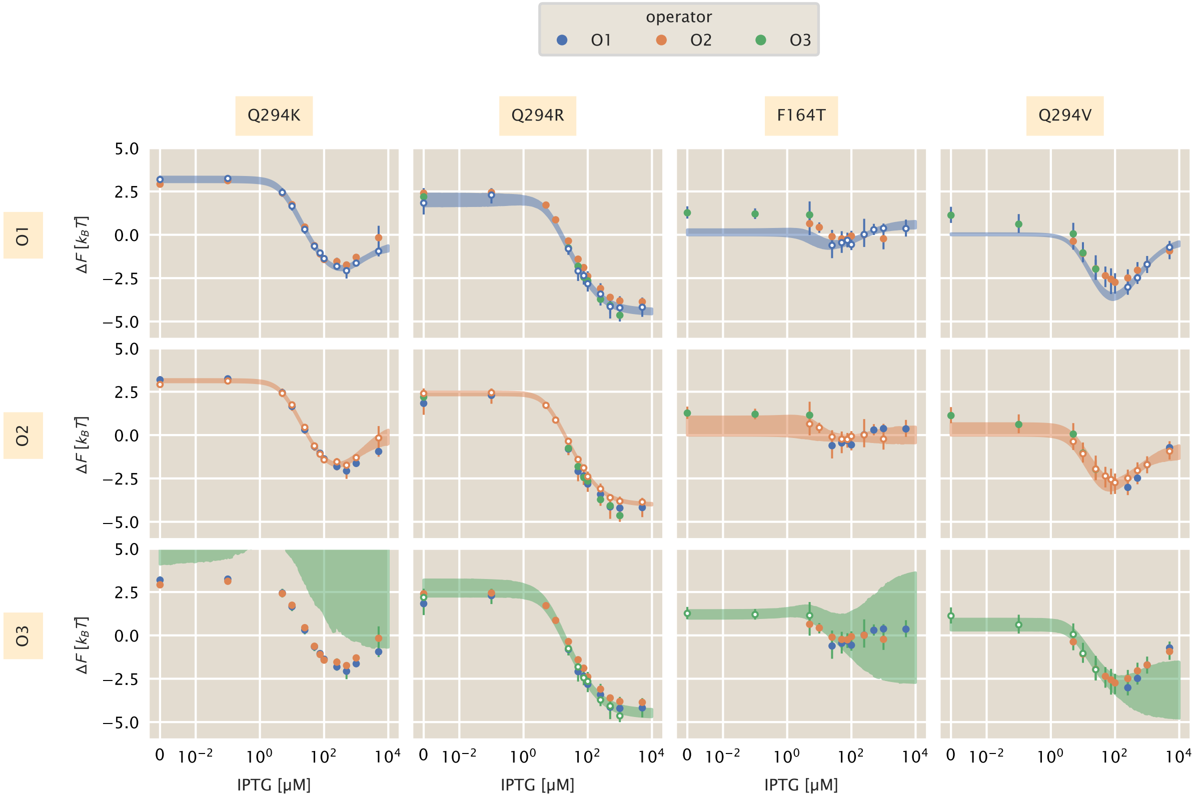

Fig. S20. Comparison of choice of fit strain on predicted ΔF profiles. Rows correspond to the operator of the strain to which the parameters were fit. Columns correspond to mutations. Points are colored by their operator sequence. The data corresponding to the operator of the fit strain are shown as white-faced points.

Table S2. Inferred values of K_A, K_I, and $\Delta\varepsilon_{AI}$ for inducer binding domain mutants. Values reported are the mean of the posterior distribution with the upper and lower bounds of the 95% credible region.

Mutant	Operator	$\mathbf{K_A}$ [μM]	$\mathbf{K_I}$ [μM]	$\Delta\varepsilon_{AI}$ [$\mathbf{k_BT}$]
F161T	O1	290^{+60}_{-56}	$1^{+4}_{-0.98}$	4^{+5}_{-3}
	O2	165^{+90}_{-65}	3^{+6}_{-3}	1^{+5}_{-2}
	O3	110^{+700}_{-105}	7^{+5}_{-4}	$-0.9^{+0.4}_{-0.3}$
Q291K	O1	> 1 mM	410^{+150}_{-100}	$-3.2^{+0.1}_{-0.1}$
	O2	> 1 mM	310^{+70}_{-60}	$-3.11^{+0.07}_{-0.07}$
	O3	10^{+200}_{-10}	1^{+9}_{-1}	-7^{+3}_{-5}
Q291R	O1	3^{+27}_{-3}	2^{+20}_{-2}	$-1.9^{+0.4}_{-0.3}$
	O2	9^{+20}_{-9}	8^{+20}_{-8}	$-2.32^{+0.01}_{-0.09}$
	O3	6^{+24}_{-6}	9^{+30}_{-9}	$-2.6^{+0.4}_{-0.5}$
Q291V	O1	> 1 mM	3^{+13}_{-3}	6^{+4}_{-4}
	O2	650^{+450}_{-250}	8^{+8}_{-8}	3^{+6}_{-3}
	O3	100^{+400}_{-90}	22^{+33}_{-18}	$0.1^{+0.8}_{-0.6}$

8. Comparing Parameter Values To The Literature

In this section, we compare and contrast the biophysical parameter values we use to characterize the wild-type Lac repressor with the rich literature that consists of *in vitro* and *in vivo* experiments. This section has an accompanying interactive figure available on the paper website which allows the reader to examine different combinations of parameter values and their agreement or disagreement with data taken from Refs. (1, 2, 9).

While the mutations used in this work and those in Ref. (8) are the same, we report significantly different values for the inducer binding, DNA binding parameters, and the relative energy difference between active and inactive states of the mutant repressors. The apparent disagreement of parameter values between the present work and those presented in Ref. (8) in part stem from different treatments of the values for the wild-type Lac repressor. Since its isolation by Gilbert and Müller-Hill in the 1960's (20), the Lac repressor has been the subject of intense biochemical and structural study. Many measurements of the inducer and DNA binding kinetics of the repressor *in vitro* [such as Ref. (21)] and their values have informed the fitting of other parameters from measurements *in vivo* [such as Refs. (8, 22)]. All of these measurements, however, do not *directly* measure the DNA- or inducer-binding kinetics nor the equilibrium constant between the active and inactive states of the repressor. To properly estimate the parameters, one must either have direct measurement of a subset of the parameters or make assumptions regarding the states of the system. Examples of the estimated allosteric parameter values of the wild-type LacI repressor from our previous work (2), that of Daber *et al.* 2011 (8), and *in vitro* measurements from O'Gorman *et al.* 1980 (21) are given in Table S3. The theoretical predictions for the fold-change in gene expression, along with values reported in (22), can be seen using the interactive figure on the paper website, where the reader can also enter their own parameter values and independently assess the agreement or lack thereof with the data.

It is notable that differences between the various references shown in Table S3 can be drastic, in some cases differing by almost an order of magnitude. Of particular note is the disagreement in the energy difference between the active and inactive states of the repressor, $\Delta\varepsilon_{AI}$. Daber *et al.* 2011 determines a negative value of $\Delta\varepsilon_{AI}$ meaning that the inactive state of the repressor is energetically favorable to the active state. In stark contrast is the value reported in our previous work of $+4.5\,k_BT$, implying that the active state is significantly more stable than the inactive state. The now seminal *in vitro* measurements reported in O'Gorman *et al.* 1980 suggest that the two states are nearly energetically equivalent.

The wide range of these reported values demonstrate that such thermodynamic models are highly degenerate, meaning that many combinations of parameter values can result in nearly equally good descriptions of the data. To illustrate this point, we estimated K_A, K_I, and DNA binding energy $\Delta\varepsilon_{RA}$ for each operator to the data reported in Refs. (1, 2, 9), using the three values of $\Delta\varepsilon_{AI}$ shown in Table S3. The resulting fits can be seen in Fig. S21. Despite the drastically different values of $\Delta\varepsilon_{AI}$ it is possible to generate adequate fits by modulating the other parameters. The parameter values of these fits, reported in Table S4 further illustrate this point as they differ significantly from one another.

The theoretically predicted fold-change in the presence of multiple promoters, shown in Fig. S21(E) is perhaps the most informative test of the parameter values. The theoretical advancements made in Weinert *et al.* 2014 (10) provide a means to mathematically grasp the intricacies of plasmid-borne expression through calculation of the chemical potential. This formalism transforms the intimidating combinatorics of R repressors and N specific binding sites (i.e. plasmid reporter genes) into a two-state system where one can compute an *effective* repressor copy number regulating a single promoter. Using this formalism, the input-output function for the fold-change in gene expression can be written as

$$\text{fold-change} = \frac{1}{1 + \lambda_R(c)e^{-\beta\Delta\varepsilon_{RA}}}, \quad [40]$$

where the effective repressor copy number (termed the fugacity) is denoted as $\lambda_R(c)$. In the supplemental material of Razo-Mejia *et al.* 2018 (2), we show that the inflection point of Eq. 40, whose curves shown in the bottom right-hand plot of Fig. S21, is located where the number of specific binding sites for the repressor is approximately equal to the number of repressors in the active state. Using this key feature, one can infer $\Delta\varepsilon_{AI}$ given prior knowledge of $\Delta\varepsilon_{RA}$ and the total number of repressors per cell. As shown in Fig. S21, using values of $\Delta\varepsilon_{AI}$ from Refs. (2, 21) approximately agree with the measurements whereas the predicted curves using $\Delta\varepsilon_{AI}$ from Daber *et al.* 2011 (8) overestimates the fold-change, even though these values accurately describe the simple-repression data shown in the other panels of Fig. S21.

Without some direct *in vivo* measurements of these parameters, one must make assumptions about the system to make any quantitative progress. We chose to use the parameter values defined in our laboratory as the repressor fugacity provides us with an independent, albeit indirect, measurement of $\Delta\varepsilon_{AI}$ which other works such as Daber *et al.* and O'Gorman *et al.* do not. Both of these works determine all of parameter values simultaneously by fitting to a single set of measurements. While these measurements accurately describe their data, their parameter values are less successful in accounting for data from Brewster *et al.* 2014, Razo-Mejia *et al.* 2018, and Garcia *et al.* 2011. In the context of this work, we emphasize that we make many of the same qualitative conclusions as in Daber *et al.* 2011 with respect to the effects of the mutations using our particular set of parameter values.

While we use different values for $\Delta\varepsilon_{AI}$, the qualitative results between this work and that of Ref. (8) are in agreement. For example, both works determine that mutations in the DNA binding domain alter only the DNA binding affinity whereas the mutations in the inducer binding pocket affect only the allosteric parameters. Because the biological variables such as repressor and reporter gene copy number are tightly controlled in our system, we are able to more precisely measure features of the induction profiles such as the leakiness in gene expression. This ability allows us to detect changes in the active/inactive equilibrium which were masked in Daber *et al.* 2011 by the experimental design.While the precise parameter values may be different between

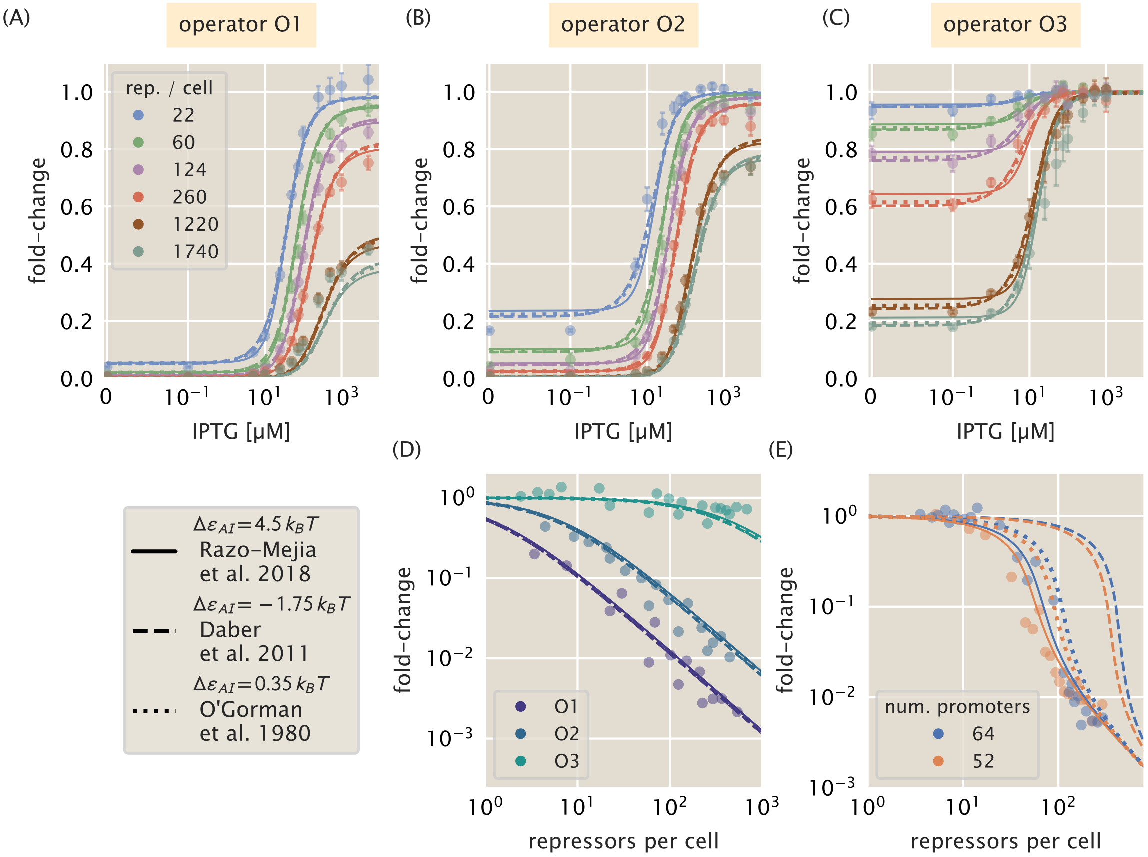

Fig. S21. Degenerate fits of data from Refs. (1, 2, 9) using different values for active/inactive state energy difference $\Delta\varepsilon_{AI}$. In all plots, the solid, dashed, and dotted lines correspond to the best-fit curves conditioned on the data using parameter values for $\Delta\varepsilon_{AI}$ of $4.5\,k_BT$, $-1.75\,k_BT$, and $0.35\,k_BT$, respectively. Induction profiles from Razo-Mejia *et al.* 2018 for operators O1 (A), O2 (B), and O3 (C) are shown as points and errors which correspond to the mean and standard error of at least 10 biological replicates. (D) Leakiness measurements of the simple repression motif with one unique regulated reporter gene. Data shown are from Refs. (1, 9). (E) The transcription factor titration effect. For gene expression measurements on plasmids, the fold-change as a function of repressor copy number exhibits strong nonlinearities. We used this effect as a way to independently infer the parameter $\Delta\varepsilon_{AI}$ and it can be seen that this breaks the degeneracy between different parameters.t

publications, the exploration of free energy differences resulting from mutations are parameter-value independent and any parameter disagreements do not change our theoretical model. Fig. 2 of the main text is presented with no knowledge of parameter values – it simply shows the mathematics of the model. While the value of ΔF will ultimately depend on the parameter values, the formalism of this work remains agnostic to the parameter values and can be a useful tool for classifying mutations and couple the sequence-level variation to the systems-level response.

Table S3. **Thermodynamic parameter values of wild-type LacI from the literature.**

Parameter	Value	Reference
$\Delta\varepsilon_{AI}$	$\geq 4.5\,k_BT$	Razo-Mejia 2018 (2)
	$-1.7\,k_BT$	Daber 2011 (8)
	$0.35\,k_BT$	O'Gorman 1980 (21)
K_A	$139^{+22}_{-20}\,\mu$M	Razo-Mejia 2018(2)
	$16\,\mu$M	Daber 2011(8)
	$133\,\mu$M	O'Gorman 1980(21)
K_I	$0.53^{+0.01}_{-0.01}\,\mu$M	Razo-Mejia 2018(2)
	$2\,\mu$M	Daber 2011(8)
	4μM	O'Gorman 1980(21)

Table S4. **Estimated parameters from global fits of data from Refs. (1, 2, 9) using $\Delta\varepsilon_{AI}$ values from Table R1**

Parameter	Value	$\Delta\varepsilon_{AI}$ Reference Value
$\Delta\varepsilon_{RA}$ (O1 operator)	$-15.1^{+0.1}_{-0.1}\, k_BT$	$4.5\, k_BT$ (2)
	$-17.1^{+0.1}_{-0.1}\, k_BT$	$-1.75\, k_BT$ (8)
	$-15.7^{+0.1}_{-0.1}\, k_BT$	$0.35\, k_BT$ (21)
$\Delta\varepsilon_{RA}$ (O2 operator)	$-13.4^{+0.1}_{-0.1}\, k_BT$	$4.5 k_BT$ (2)
	$-15.4^{+0.1}_{-0.1}\, k_BT$	$-1.75\, k_BT$ (8)
	$-14.0^{+0.1}_{-0.1}\, k_BT$	$0.35\, k_BT$ (21)
$\Delta\varepsilon_{RA}$ (O3 operator)	$-9.21^{+0.06}_{-0.06}\, k_BT$	$4.5 k_BT$ (2)
	$-11.29^{+0.06}_{-0.06}\, k_BT$	$-1.75\, k_BT$ (8)
	$-9.85^{+0.06}_{-0.05}\, k_BT$	$0.35\, k_BT$ (21)
K_A	$225^{+10}_{-10}\, \mu$M	$4.5\, k_BT$ (2)
	$290^{+20}_{-20}\, \mu$M	$-1.75\, k_BT$ (8)
	$270^{+20}_{-20}\, \mu$M	$0.35\, k_BT$ (21)
K_I	$0.81^{+0.05}_{-0.05}\, \mu$M	$4.5\, k_BT$ (2)
	$8.2^{+0.5}_{-0.5}\, \mu$M	$-1.75\, k_BT$ (8)
	$5.5^{+0.5}_{-0.3}\, \mu$M	$0.35\, k_BT$ (21)

9. Parameter Estimation Using All Induction Profiles

In the main text and Sec. 4 and 6 of this supplementary text, we have laid out our strategy for inferring the the various parameters of our model to a single induction profile and using the resulting values to predict the free energy and induction profiles of other strains. In this section, we estimate the parameters using all induction profiles of a single mutant and using the estimated values to predict the free energy profiles.

The inferred DNA binding energies considering induction profiles of all repressor copy numbers for the three DNA binding mutants are reported in Tab. S5. These parameters are close to those reported in Tab. S1 for each repressor copy number with Q18A showing the largest differences. The resulting induction profiles and predicted change in free energy for these mutants can be seen in Fig. S22. Overall, the induction profiles match the data to an appreciable agree. We acknowledge that even when using *all* repressor copy numbers, the fit to Q18A remains imperfect. However we contend that this disagreement is comparable to that observed in (2) which described the induction profile of the wild-type repressor. We find that the predicted change in free energy [bottom row in Fig. S22(B)] narrows compared to that in Fig. S13 and Fig. 3 of the main text, confirming that considering all induction profiles improves our inference of the most-likely DNA binding energy. There appears to be a very slight trend in the ΔF for Q18A at higher inducer concentrations, though the overall change in free energy from 0 to 5000 μM IPTG is small.

We also estimated the allosteric parameters (K_A, K_I, and $\Delta\varepsilon_{AI}$) for all inducer binding domain mutations using the induction profiles of all three operator sequences. The values, reported in Tab. S6 are very similar to those estimated from a single induction profile (Tab. S2). We note that for Q291R, it is difficult to properly estimate the values for K_A and K_I as the observed induction profile is approximately flat. The induction profiles and predicted change in free energy for each inducer binding mutant is shown in Fig. S23. We see notable improvement in the agreement between the induction profiles and the observed data, indicating that considering all data significantly shrinks the uncertainty of each parameter. The predicted change in free energy is also improved compared to that shown in Fig. S20. We emphasize that the observed free energy difference for each point assumes no knowledge of the underlying parameters and comes directly from measurements. The remarkable agreement between the predicted free energy and the observations illustrates that redetermining the allosteric parameters is sufficient to describe how the free energy changes as a result of the mutation.

Table S5. Estimated DNA binding energies for each DNA binding domain mutant using all repressor copy numbers

Mutant	$\Delta\varepsilon_{RA}$ $[k_BT]$
Y17I	$-9.81^{+0.04}_{-0.08}$
Q18A	$-10.60^{+0.07}_{-0.07}$
Q18M	$-15.61^{+0.05}_{-0.05}$

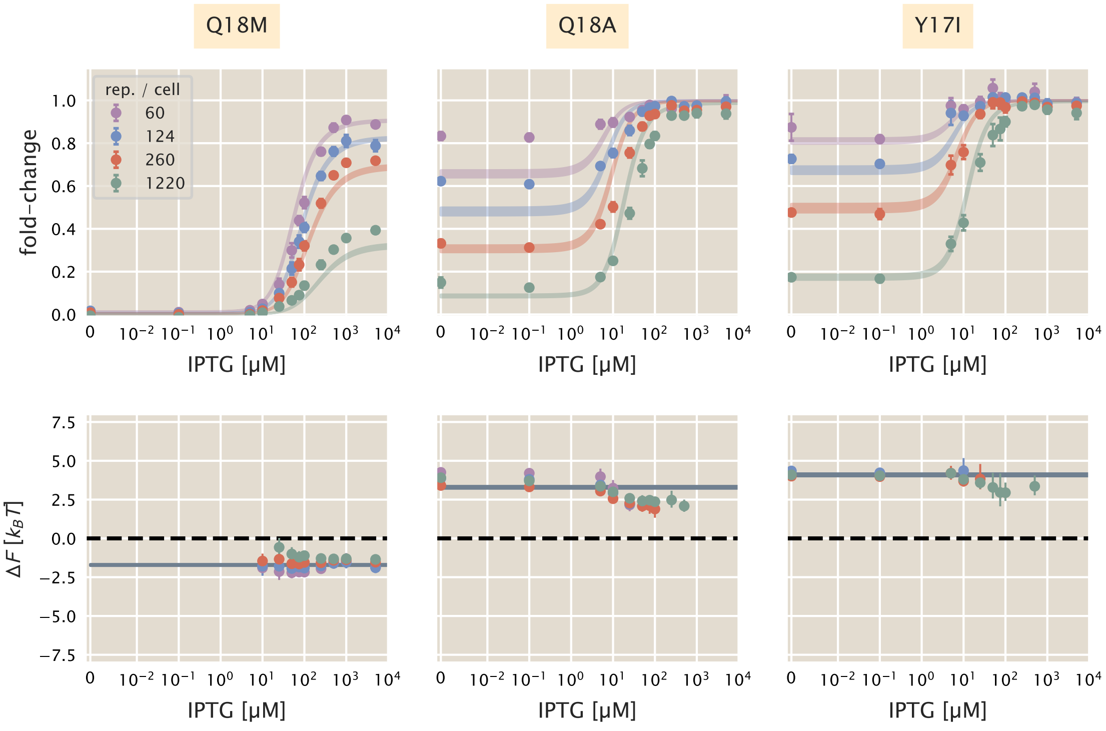

Fig. S22. Induction profiles and predicted change in free energy using parameters estimated from the complete data sets. Top row shows fold-change measurements (points) as mean and standard error with ten to fifteen biological replicates. Shaded lines correspond to the 95% credible regions of the induction profiles using the estimated values of the DNA binding energies reported in Tab. S5. Bottom row shows the 95% credible regions of the predicted change in free energy (shaded lines) along with the inferred free energy of data shown in the top row. In all plots, the inducer concentration is shown on a symmetric log scale with linear scaling between 0 and 10^{-2} μM and log scaling elsewhere.

Table S6. Estimated values for K_A, K_I, and $\Delta\varepsilon_{AI}$ for inducer binding domain mutations using induction profiles of all operator sequences.

**Mutant**	K_A [μM]	K_I [μM]	$\Delta\varepsilon_{AI}$ [k_BT]
F161T	300^{+60}_{-60}	$12.7^{+0.1}_{-0.1}$	$-0.9^{+0.3}_{-0.3}$
Q291K	> 1 mM	330^{+60}_{-70}	$-3.17^{+0.07}_{-0.07}$
Q291R	> 1 mM	> 1 mM	$-2.4^{+0.2}_{-0.2}$
Q291V	> 1 mM	53^{+17}_{-13}	$0^{+0.3}_{-0.3}$

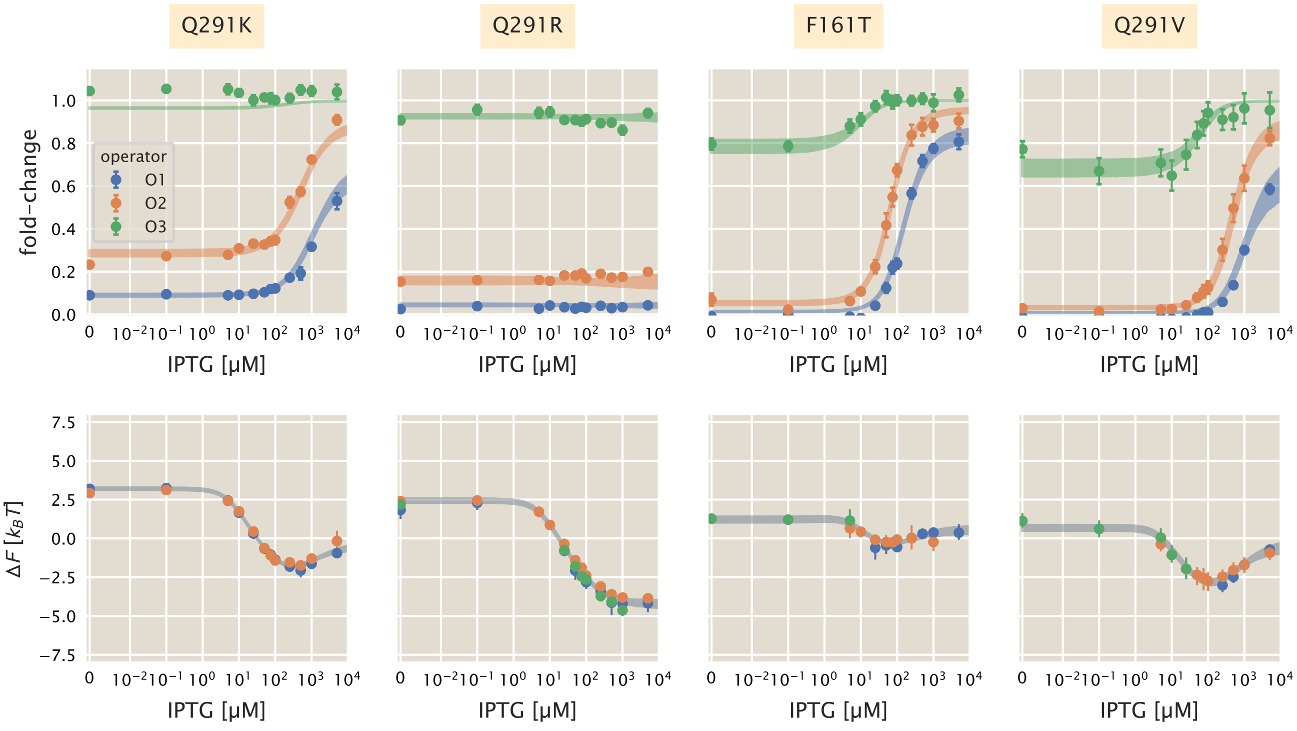

Fig. S23. Induction profiles and predicted change in free energy using parameters estimated from the complete data sets for inducer binding domain mutnats. Top row shows fold-change measurements (points) as mean and standard error with ten to fifteen biological replicates. Shaded lines correspond to the 95% credible regions of the induction profiles using the estimated values of the allosteric parameters reported in Tab. S6. Bottom row shows the 95% credible regions of the predicted change in free energy (shaded lines) along with the inferred free energy of data shown in the top row. In all plots, the inducer concentration is shown on a symmetric log scale with linear scaling between 0 and 10^{-2} μM and log scaling elsewhere.

10. Generalizability of Data Collapse To Other Regulatory Architectures

In Razo-Mejia *et al.* 2018 (2) and in the main text we have stated that the input-output function for the fold-change in gene expression can be rewritten in the form of a Fermi function. However, this result can be derived directly by coarse graining the transcription factor bound and unbound states of the systems' partition function. In this section, we show how coarse graining of promoter occupancy states results in a general Fermi function so long as the transcription factor bound states and transcriptionally active states do not overlap.

As shown in Sec. 1, the partition function $\mathcal{Z}$ for the simple repression motif (ignoring allosteric control) can be enumerated as

$$\mathcal{Z} = \overbrace{1 + \frac{P}{N_{NS}} e^{-\beta \Delta \varepsilon_P}}^{\text{repressor unbound states}} + \underbrace{\frac{R}{N_{NS}} e^{-\beta \Delta \varepsilon_R}}_{\text{repressor bound state}} = \mathcal{Z}_{\neg r} + \mathcal{Z}_r, \quad [41]$$

where R is the number of repressors, P is the number of polymerases, N_{NS} is the number of nonspecific binding sites and $\Delta\varepsilon_P$ and $\Delta\varepsilon_R$ are the binding energies of the polymerase and repressor to the DNA, respectively. The states can be grouped into either repressor bound states (right-hand terms) or repressor unbound states (left hand terms), denoted as $\mathcal{Z}_r$ and $\mathcal{Z}_{\neg r}$, respectively. The probability of the repressor *not* being bound to the promoter can be computed as

$$P(\neg r) = \frac{\mathcal{Z}_{\neg r}}{\mathcal{Z}_{\neg r} + \mathcal{Z}_r}. \quad [42]$$

In this coarse-grained description, the transcriptionally active states are separate from the repressor bound states. Thus, to calculate the fold-change in gene expression, we can compute the ratio of $P(\neg r | R > 0)$ when repressor is present to $P(\neg r \mid R = 0)$ when repressor is absent. As the latter term is equal to 1, the fold-change in gene expression is equivalent to Eq. 42. We can compute an *effective* free energy $\tilde{F}$ of each coarse-grained state as

$$\tilde{F}_{\neg r} = -k_B T \log \mathcal{Z}_{\neg r}\, ; \, \tilde{F}_r = -k_B T \log \mathcal{Z}_r. \quad [43]$$

With an effective energy in hand, we can write Eq. 42 (which is equivalent to the fold-change) in terms of the Boltzmann weights of these two coarse-grained states as

$$\text{fold-change} = \frac{e^{-\beta \tilde{F}_{\neg r}}}{e^{-\beta \tilde{F}_{\neg r}} + e^{-\beta \tilde{F}_r}}. \quad [44]$$

A simple rearrangement of this result produces a Fermi function

$$\text{fold-change} = \frac{1}{1 + e^{-\beta \tilde{F}}}, \quad [45]$$

where $\tilde{F}$ is the effective free energy of the system defined as

$$\tilde{F} = -k_B T \left(\log \mathcal{Z}_r - \log \mathcal{Z}_{\neg r}\right). \quad [46]$$

In the case of the simple repression motif, we apply the weak promoter approximation which is based on the fact that $\frac{P}{N_{NS}} e^{-\beta \Delta \varepsilon_P} \ll 1$, reducing $\mathcal{Z}_{\neg r} = 1$. With this approximation, the effective free energy $\tilde{F}$ defined in Eq. 46 can be interpreted as the free energy between the repressed and transcriptionally active states. In general, this coarse-graining approach can be applied to any regulatory architecture in which the transcription factor bound states and transcriptionally active states share no overlapping substates. Fig. S24 shows various repression based architectures along with the coarse graining of states and the effective free energy.

This approach cannot be applied to architectures in which the transcription factor bound and transcriptionally active states cannot be separated. One such example is the case of simple activation in which the binding of an activator increases gene expression. For this architecture, the total partition function [as derived in Ref. (23)] is

$$\mathcal{Z} = \overbrace{1 + \frac{P}{N_{NS}} e^{-\beta \Delta \varepsilon_P}}^{\text{activator unbound states}} + \underbrace{\frac{A}{N_{NS}} e^{-\beta \Delta \varepsilon_A} + \frac{A}{N_{NS}} \frac{P}{N_{NS}} e^{-\beta(\Delta \varepsilon_A + \Delta \varepsilon_P + \varepsilon_{\text{interaction}})}}_{\text{activator bound states}} = \mathcal{Z}_{\neg a} + \mathcal{Z}_a. \quad [47]$$

Here we've used A to denote the number of activators, $\Delta\varepsilon_A$ is the binding energy of the activator to its specific binding site, and $\varepsilon_{\text{interaction}}$ is the interaction energy between the activator and polymerase which makes the activator-polymerase-DNA state more energetically favorable to the polymerase-DNA state. Unlike in the case of repression, the transcriptionally active states are present in the $\mathcal{Z}_a$. While we can compute the probability of the activator not being bound $P(\neg a)$, this is not equivalent to the fold-change in gene expression as was the case for simple repression. Rather, the fold-change in gene expression can be written as

$$\text{fold-change} = \frac{\mathcal{Z}_a - 1 + \mathcal{Z}_{\neg a} - \frac{A}{N_{NS}} e^{-\beta \Delta \varepsilon_A}}{\mathcal{Z}_a + \mathcal{Z}_{\neg a}} \frac{\mathcal{Z}_a}{\mathcal{Z}_a - 1}. \quad [48]$$

This is a more cumbersome expression than in the case for simple repression, and cannot be massaged into a one-parameter description of the fold-change. Thus, the analysis presented here is only applicable to cases in which the occupancy of the promoter by either the polymerase or the transcription factor are mutually exclusive.

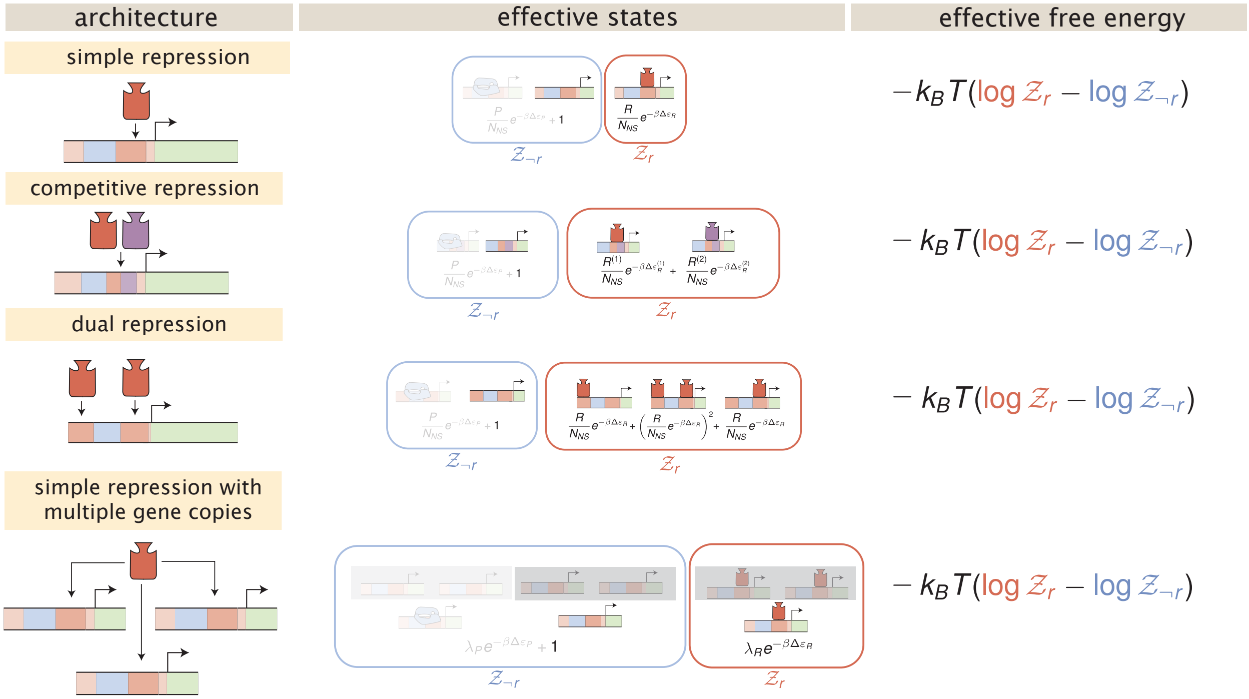

Fig. S24. Various repression-based regulatory architectures and their coarse-grained states. The left column shows a schematic of the regulatory architecture. The middle column shows the equilibrium states of the system coarse grained into repressor bound (red boxes) and repressor unbound (blue boxes) states. States in which the polymerase is bound is shaded in white and are neglected by the weak promoter approximation. The right column illustrates that the formulation of the effective free energy is the same for all architectures, although the formulation of $\mathcal{Z}_{\neg r}$ and $\mathcal{Z}_r$ are different between the examples. The bottom row illustrates the coarse graining of a simple repression motif in which the same repressor regulates multiple copies of the same gene. Rather than considering all states, the gene expression can calculated by considering one promoter and an effective copy number, described by the repressor fugacity λ_r and is derived in (10). Grayed-out states illustrate this further level of coarse graining.

Strain and Oligonucleotide Information

Table S7. *Escherichia coli* strains used in this work

Class	LacI Mutant	Operator	Rep. per Cell	Genotype	Plasmid
–	–	–	22	MG1655::Δ*lacZYA*	pZS4*1-mCherry
–	–	O1	0	MG1655::Δ*lacIZYA*;*galK*<>25O1+11-YFP	pZS4*1-mCherry
–	–	O2	0	MG1655::Δ*lacIZYA*;*galK*<>25O2+11-YFP	pZS4*1-mCherry
–	–	O3	0	MG1655::Δ*lacIZYA*;*galK*<>25O3+11-YFP	pZS4*1-mCherry
WT	WT	O2	260	MG1655::Δ*lacIZYA*;*galK*<>25O2+11-YFP;*ybcN*<>3*1-RBS1027LacI	pZS4*1-mCherry
DNA	Y17I	O2	60	MG1655::Δ*lacIZYA*;*galK*<>25O2+11-YFP;*ybcN*<>3*1-RBS1147LacI(Y17I)	pZS4*1-mCherry
DNA	Y17I	O2	124	MG1655::Δ*lacIZYA*;*galK*<>25O2+11-YFP;*ybcN*<>3*1-RBS446LacI(Y17I)	pZS4*1-mCherry
DNA	Y17I	O2	260	MG1655::Δ*lacIZYA*;*galK*<>25O2+11-YFP;*ybcN*<>3*1-RBS1027LacI(Y17I)	pZS4*1-mCherry
DNA	Y17I	O2	1220	MG1655::Δ*lacIZYA*;*galK*<>25O2+11-YFP;*ybcN*<>3*1-RBS1LacI(Y17I)	pZS4*1-mCherry
DNA	Q18A	O2	60	MG1655::Δ*lacIZYA*;*galK*<>25O2+11-YFP;*ybcN*<>3*1-RBS1147LacI(Q18A)	pZS4*1-mCherry
DNA	Q18A	O2	124	MG1655::Δ*lacIZYA*;*galK*<>25O2+11-YFP;*ybcN*<>3*1-RBS446LacI(Q18A)	pZS4*1-mCherry
DNA	Q18A	O2	260	MG1655::Δ*lacIZYA*;*galK*<>25O2+11-YFP;*ybcN*<>3*1-RBS1027LacI(Q18A)	pZS4*1-mCherry
DNA	Q18A	O2	1220	MG1655::Δ*lacIZYA*;*galK*<>25O2+11-YFP;*ybcN*<>3*1-RBS1LacI(Q18A)	pZS4*1-mCherry
DNA	Q18M	O2	60	MG1655::Δ*lacIZYA*;*galK*<>25O2+11-YFP;*ybcN*<>3*1-RBS1147LacI(Q18M)	pZS4*1-mCherry
DNA	Q18M	O2	124	MG1655::Δ*lacIZYA*;*galK*<>25O2+11-YFP;*ybcN*<>3*1-RBS446LacI(Q18M)	pZS4*1-mCherry
DNA	Q18M	O2	260	MG1655::Δ*lacIZYA*;*galK*<>25O2+11-YFP;*ybcN*<>3*1-RBS1027LacI(Q18M)	pZS4*1-mCherry
DNA	Q18M	O2	1220	MG1655::Δ*lacIZYA*;*galK*<>25O2+11-YFP;*ybcN*<>3*1-RBS1LacI(Q18M)	pZS4*1-mCherry
IND	F161T	O1	260	MG1655::Δ*lacIZYA*;*galK*<>25O1+11-YFP;*ybcN*<>3*1-RBS1027LacI(F161T)	pZS4*1-mCherry
IND	F161T	O2	260	MG1655::Δ*lacIZYA*;*galK*<>25O2+11-YFP;*ybcN*<>3*1-RBS1027LacI(F161T)	pZS4*1-mCherry
IND	F161T	O3	260	MG1655::Δ*lacIZYA*;*galK*<>25O3+11-YFP;*ybcN*<>3*1-RBS1027LacI(F161T)	pZS4*1-mCherry
IND	Q291V	O1	260	MG1655::Δ*lacIZYA*;*galK*<>25O1+11-YFP;*ybcN*<>3*1-RBS1027LacI(Q291V)	pZS4*1-mCherry
IND	Q291V	O2	260	MG1655::Δ*lacIZYA*;*galK*<>25O2+11-YFP;*ybcN*<>3*1-RBS1027LacI(Q291V)	pZS4*1-mCherry
IND	Q291V	O3	260	MG1655::Δ*lacIZYA*;*galK*<>25O3+11-YFP;*ybcN*<>3*1-RBS1027LacI(Q291V)	pZS4*1-mCherry
IND	Q291K	O1	260	MG1655::Δ*lacIZYA*;*galK*<>25O1+11-YFP;*ybcN*<>3*1-RBS1027LacI(Q291K)	pZS4*1-mCherry
IND	Q291K	O2	260	MG1655::Δ*lacIZYA*;*galK*<>25O2+11-YFP;*ybcN*<>3*1-RBS1027LacI(Q291K)	pZS4*1-mCherry
IND	Q291K	O3	260	MG1655::Δ*lacIZYA*;*galK*<>25O3+11-YFP;*ybcN*<>3*1-RBS1027LacI(Q291K)	pZS4*1-mCherry
IND	Q291R	O1	260	MG1655::Δ*lacIZYA*;*galK*<>25O1+11-YFP;*ybcN*<>3*1-RBS1027LacI(Q291R)	pZS4*1-mCherry
IND	Q291R	O2	260	MG1655::Δ*lacIZYA*;*galK*<>25O2+11-YFP;*ybcN*<>3*1-RBS1027LacI(Q291R)	pZS4*1-mCherry
IND	Q291R	O3	260	MG1655::Δ*lacIZYA*;*galK*<>25O3+11-YFP;*ybcN*<>3*1-RBS1027LacI(Q291R)	pZS4*1-mCherry
DBL	Y17I-F161T	O2	260	MG1655::Δ*lacIZYA*;*galK*<>25O2+11-YFP;*ybcN*<>3*1-RBS1027LacI(Y17IF161T)	pZS4*1-mCherry
DBL	Y17I-Q291V	O2	260	MG1655::Δ*lacIZYA*;*galK*<>25O2+11-YFP;*ybcN*<>3*1-RBS1027LacI(Y17IQ291V)	pZS4*1-mCherry
DBL	Y17I-Q291K	O2	260	MG1655::Δ*lacIZYA*;*galK*<>25O2+11-YFP;*ybcN*<>3*1-RBS1027LacI(Y17IQ291K)	pZS4*1-mCherry
DBL	Q18A-F161T	O2	260	MG1655::Δ*lacIZYA*;*galK*<>25O2+11-YFP;*ybcN*<>3*1-RBS1027LacI(Q18AF161T)	pZS4*1-mCherry
DBL	Q18A-Q291V	O2	260	MG1655::Δ*lacIZYA*;*galK*<>25O2+11-YFP;*ybcN*<>3*1-RBS1027LacI(Q18AQ291V)	pZS4*1-mCherry
DBL	Q18A-Q291K	O2	260	MG1655::Δ*lacIZYA*;*galK*<>25O2+11-YFP;*ybcN*<>3*1-RBS1027LacI(Q18AQ291K)	pZS4*1-mCherry
DBL	Q18M-F161T	O2	260	MG1655::Δ*lacIZYA*;*galK*<>25O2+11-YFP;*ybcN*<>3*1-RBS1027LacI(Q18MF161T)	pZS4*1-mCherry
DBL	Q18M-Q291V	O2	260	MG1655::Δ*lacIZYA*;*galK*<>25O2+11-YFP;*ybcN*<>3*1-RBS1027LacI(Q18MQ291V)	pZS4*1-mCherry
DBL	Q18M-Q291K	O2	260	MG1655::Δ*lacIZYA*;*galK*<>25O2+11-YFP;*ybcN*<>3*1-RBS1027LacI(Q18MQ291K)	pZS4*1-mCherry

Table S8. Oligonucleotides used for mutant generation.

Primer Name	Sequence (5'$\rightarrow$3')	Description	Method
10.1	`acctctgcggaggggaagcgtgaacctctcacaagacggcatcaaattacactagcaacaccagaacagc`	Integration into *ybcN* locus	λ-Red Recombineering
10.3	`ctgtagatgtgtccgttcatgacacgaataagcggtgtagccattacgccggctaatgcacccagtaagg`	Integration into *ybcN* locus	λ-Red Recombineering
GCMWC-001	`ccggcatactctgcgaca`	Amplification of plasmid	QuickChange Mutagenesis
GCMWC-002	`gtgtctcttatATGaccgtttcccgc`	Q18M Mutation (`CAG`$\rightarrow$`ATG`)	QuickChange Mutagenesis
GCMWC-003	`tgtctcttatGCGaccgtttcccgc`	Q18A Mutation (`CAG`$\rightarrow$`GCG`)	QuickChange Mutagenesis
GCMWC-004	`gttaacggcgggatataac`	Amplification of plasmid	QuickChange Mutagenesis
GCMWC-005	`caccatcaaaGTGgattttcgcctgc`	Q291V Mutation (`CAG` $\rightarrow$ `GTG`)	QuickChange Mutagenesis
GCMWC-006	`caccatcaaaAAGgattttcgcc`	Q291K Mutation (`CAG`$\rightarrow$`AAG`)	QuickChange Mutagenesis
GCMWC-007	`cagtattattACCtcccatgaagacgg`	F161T Mutation (`TTC`$\rightarrow$`ACC`)	QuickChange Mutagenesis
GCMWC-008	`ttgatgggtgtctggtcag`	Amplification of plasmid	QuickChange Mutagenesis
GCMWC-009	`gcatactctgcgacatcgtataa`	Amplification of plasmid	QuickChange Mutagenesis
GCMWC-010	`cggtgtctctATTcagaccgtttc`	Y17I Mutation (`TAT`$\rightarrow$`ATT`)	QuickChange Mutagenesis
GCMWC-017	`ccatcaaaAGGgattttcgcctgctggggcaaaccag`	Q291R Mutation (`CAG`$\rightarrow$`AGG`)	Gibson Assembly
GCMWC-018	`ggcgaaaatcCCTtttgatggtggttaacggcggg`	Q291R Mutation (`CTG`$\rightarrow$`CCT`)	Gibson Assembly

References

1. Garcia HG, Phillips R (2011) Quantitative dissection of the simple repression input-output function. *Proceedings of the National Academy of Sciences of the United States of America* 108(29):12173–8.
2. Razo-Mejia M, et al. (2018) Tuning Transcriptional Regulation through Signaling: A Predictive Theory of Allosteric Induction. *Cell Systems* 6(4):456–469.
3. Ackers GK, Johnson AD, Shea MA (1982) Quantitative model for gene regulation by lambda phage repressor. *Proceedings of the National Academy of Sciences of the United States of America* 79(4):1129–33.
4. Buchler NE, Gerland U, Hwa T (2003) On schemes of combinatorial transcription logic. *Proceedings of the National Academy of Sciences of the United States of America* 100(9):5136–41.
5. Vilar JM, Leibler S (2003) DNA Looping and Physical Constraints on Transcription Regulation. *Journal of Molecular Biology* 331(5):981–989.
6. Bintu L, et al. (2005) Transcriptional regulation by the numbers: Applications. *Current Opinion in Genetics and Development* 15(2):125–135.
7. Kuhlman T, Zhang Z, Saier MH, Hwa T (2007) Combinatorial transcriptional control of the lactose operon of *Escherichia coli*. *Proceedings of the National Academy of Sciences of the United States of America* 104(14):6043–6048.
8. Daber R, Sochor MA, Lewis M (2011) Thermodynamic analysis of mutant lac repressors. *Journal of Molecular Biology* 409(1):76–87.
9. Brewster RC, et al. (2014) The transcription factor titration effect dictates level of gene expression. *Cell* 156(6):1312–1323.
10. Weinert FM, Brewster RC, Rydenfelt M, Phillips R, Kegel WK (2014) Scaling of Gene Expression with Transcription-Factor Fugacity. *Physical Review Letters* 113(25):1–5.
11. Klumpp S, Hwa T (2008) Growth-rate-dependent partitioning of RNA polymerases in bacteria. *Proceedings of the National Academy of Sciences of the United States of America* 105(51):20245–20250.
12. Brewster RC, Jones DL, Phillips R (2012) Tuning Promoter Strength through RNA Polymerase Binding Site Design in *Escherichia coli*. *PLoS Computational Biology* 8(12).
13. Schmidt A, et al. (2015) The quantitative and condition-dependent *Escherichia coli* proteome. *Nature Biotechnology* 34(1):104–110.
14. Monod J, Wyman J, Changeux JP (1965) On the Nature of Allosteric Transitions: A Plausible Model. *Journal of molecular biology* 12:88–118.
15. Phillips R, Milo R (2015) Rates and duration in *Cell Biology by the Numbers*.
16. Milo R, Jorgensen P, Moran U, Weber G, Springer M (2010) BioNumbers—the database of key numbers in molecular and cell biology. *Nucleic Acids Research* 38(suppl_1):D750–D753.
17. Good IJ, 1950 (1950) *Probability and the Weighing of Evidence* . (New York City).
18. Talts S, Betancourt M, Simpson D, Vehtari A, Gelman A (2018) Validating Bayesian Inference Algorithms with Simulation-Based Calibration. *arXiv:1804.06788 [stat]*.
19. Carpenter B, et al. (2017) Stan: A probabilistic programming language. *Journal of Statistical Software, Articles* 76(1):1–32.
20. Gilbert W, Müller-Hill B (1966) Isolation of the lac repressor. *Proceedings of the National Academy of Sciences of the United States of America* 56(6):1891–8.
21. O'Gorman RB, et al. (1980) Equilibrium binding of inducer to lac repressor.operator DNA complex. *Journal of Biological Chemistry* 255(21):10107–10114.
22. Daber R, Sharp K, Lewis M (2009) One Is Not Enough. *Journal of Molecular Biology* 392(5):1133–1144.
23. Bintu L, et al. (2005) Transcriptional regulation by the numbers: Models. *Current Opinion in Genetics and Development* 15(2):116–124.
